# Pervasive Hitchhiking at Coding and Regulatory Sites in Humans

**DOI:** 10.1371/journal.pgen.1000336

**Published:** 2009-01-16

**Authors:** James J. Cai, J. Michael Macpherson, Guy Sella, Dmitri A. Petrov

**Affiliations:** 1 Department of Biology, Stanford University, Stanford, California, United States of America; 2 Department of Evolution, Systematics, and Ecology, The Hebrew University of Jerusalem, Givat Ram, Jerusalem, Israel; University of Oxford, United Kingdom

## Abstract

Much effort and interest have focused on assessing the importance of natural selection, particularly positive natural selection, in shaping the human genome. Although scans for positive selection have identified candidate loci that may be associated with positive selection in humans, such scans do not indicate whether adaptation is frequent in general in humans. Studies based on the reasoning of the MacDonald–Kreitman test, which, in principle, can be used to evaluate the extent of positive selection, suggested that adaptation is detectable in the human genome but that it is less common than in Drosophila or *Escherichia coli*. Both positive and purifying natural selection at functional sites should affect levels and patterns of polymorphism at linked nonfunctional sites. Here, we search for these effects by analyzing patterns of neutral polymorphism in humans in relation to the rates of recombination, functional density, and functional divergence with chimpanzees. We find that the levels of neutral polymorphism are lower in the regions of lower recombination and in the regions of higher functional density or divergence. These correlations persist after controlling for the variation in GC content, density of simple repeats, selective constraint, mutation rate, and depth of sequencing coverage. We argue that these results are most plausibly explained by the effects of natural selection at functional sites—either recurrent selective sweeps or background selection—on the levels of linked neutral polymorphism. Natural selection at both coding and regulatory sites appears to affect linked neutral polymorphism, reducing neutral polymorphism by 6% genome-wide and by 11% in the gene-rich half of the human genome. These findings suggest that the effects of natural selection at linked sites cannot be ignored in the study of neutral human polymorphism.

## Author Summary

There is much reported evidence for positive selection at specific loci in the human genome. Additional papers based on comparisons between the genomes of humans and chimpanzees have also suggested that adaptive evolution may be quite common. At the same time, it has been surprisingly hard to find unambiguous evidence that either positive or negative (background) selection is affecting genome-wide patterns of variation at neutral sites. Here, we evaluate the prevalence of positive or background selection by using two genome-wide datasets of human polymorphism. We document that levels of neutral polymorphism are substantially lower in the regions of (i) higher density of genes and/or regulatory regions, (ii) higher protein or regulatory divergence, and (iii) lower recombination. These patterns are robust to a number of possible confounding factors and suggest that effects of selection at linked sites cannot be ignored in the study of the human genome.

## Introduction

The neutral theory of molecular evolution [1] postulates that adaptive substitutions occur so rarely that they can be safely ignored in most studies in population genetics or molecular evolution. This view has dominated the field of molecular evolution for the past 40 years. However, the past 4–6 years have seen a strong challenge to this view. This challenge comes not only from numerous studies detailing specific cases of molecular adaptation in a number of organisms (for example, see [2]–[8]) but also, and most compellingly, from a number of studies that indicate that adaptation might be common on the genomic scale [9]–[17].

High rates of adaptation on the genomic scale have been inferred from the excess of substitutions in functional regions relative to neutral expectations. The neutral expectations are derived from the polymorphism data at functional and putatively neutral sites and the divergence at the neutral sites using the reasoning of the McDonald-Kreitman (MK) test [18]. The excess in the number of substitutions at functional sites over this expectation can be used to estimate the number of adaptive substitutions [10],[19]. McDonald-Kreitman approaches can be modified to account for the presence of deleterious polymorphisms in the sample and the effects of demographic processes on polymorphism [10],[20],[21]. The approach can also be extended to estimate rates of adaptation in regulatory regions [12],[22].

McDonald-Kreitman analysis indicates that adaptive evolution in functional regions might be common in a range of organisms. In Drosophila, it has been estimated that from 30 to 60% of amino acid substitutions and ∼20% of substitutions in non-coding regions are adaptive [10], [11], [16], [23]–[25]. The rate appears similarly high in *E. coli* (>56% of amino acid substitutions are adaptive) [26] but not in Arabidopsis (0–5% of amino acid substitutions are adaptive) [27] and yeast [28].

In humans, McDonald-Kreitman-based estimates have varied from zero to ∼35% of all amino acid substitutions being adaptive [15], [29]–[33]. A recent estimate by Boyko et al [21] used information from the allele spectra of nonsynonymous and synonymous SNPs in human genes and the divergence with chimpanzee orthologs to estimate that ∼10% of amino acid substitutions between humans and chimpanzees have been fixed by positive selection. Thus, some of these studies suggest that adaptation might be fairly common in humans, although probably substantially less common than in Drosophila or *E. coli*.

McDonald-Kreitman approaches are very powerful at detecting positive selection, however, they can be misleading for a variety of reasons [15],[34],[35]. For example, if the strength of purifying selection over the evolutionary period separating two species has been different than it is in the present, McDonald-Kreitman-based approaches can either over- or underestimate the rate of adaptive evolution. As these estimates do not provide consistent answers about the prevalence of adaptation in humans and because they can be misleading under plausible demographic scenarios, reaching more reliable conclusions about the importance of adaptations in humans requires the investigation of other signatures of positive selection.

An adaptive substitution reduces the level of polymorphism at neutral sites in its vicinity in a phenomenon known as a selective sweep [36]. The width of the region in which the polymorphism is reduced is inversely proportional to the local recombination rate and directly proportional to the selection coefficient associated with the adaptive substitution [37]–[39]. The reduction of polymorphism is transient and the levels of polymorphism are expected to recover within roughly *N_e_* generations [40]. In addition to the reduction of the level of polymorphism, recurrent selective sweeps may also generate other signatures such as (i) an overabundance of low-frequency alleles [41],[42], (ii) a greater proportion of high-frequency derived alleles [43],[44], (iii) unusual haplotype structures [45],[46].

A number of these expectations have been used to define signatures of positive selection for genome-wide scans for recent adaptation in humans: i.e., the detection of candidate regions that are likely to be experiencing a selective sweep at present or that have experienced one recently. For example, Nielsen et al. [30] and Kelley et al [47] used the deviation of the allele frequency spectrum from its background characteristics to detect candidate regions that may have experienced a sweep; several other methods have used summaries of haplotype structure and their deviation from the background to detect candidate regions that are undergoing a selective sweep [45],[46].

Genomic scans for positive selection are primarily used to choose candidate regions for future investigation, but their application to the quantification of positive selection or even the establishment of its prevalence is problematic. To quantify the extent of positive selection based on the deviations of these signatures from the background requires a prior expectation about the likelihood of observing them under neutrality. These expectations, however, may be sensitive to the effects of non-equilibrium demography [44], [48]–[50]. As a result, it is difficult to generate robust *a priori* expectations for these statistics under neutrality. Therefore, scans for positive selection do not, by themselves, provide reliable quantification of the extent of positive selection in humans or establish that positive selection is prevalent in humans.

To evaluate whether selective sweeps are common in the human genome, we require signatures that are unlikely to be generated by demography alone. The effects of recurrent selective sweeps (RSS) should be stronger in the regions of lower recombination and in regions of more frequent and selectively consequential adaptation. In Drosophila, for example, the level of neutral polymorphism is positively correlated with the recombination rate [51]–[53] and negatively with the rate and number of nonsynonymous substitutions in a region [9],[13],[14]. These correlations, which are expected under models of RSS but should not be generated by demography alone, support the notion of high rates of adaptation in these taxa.

Despite several compelling examples of adaptations, clear genome-wide signatures of RSS have been difficult to detect in humans. A relationship of diversity and recombination has been reported, but was attributed primarily to an association between recombination and mutation processes rather than to the effects of selected at linked sites [54]–[57], with the possible exception of telomeric and centromeric regions [58]. In turn, the relationships between levels of polymorphism and functional divergence have not yet been examined.

If the recent MK estimates of the rate of adaptive evolution are correct and approximately 10% of amino acid substitutions are adaptive [21], we should expect to see a substantial number of recent selective sweeps in the polymorphism data. Indeed, ∼7×10^4^ amino acid differences between human and chimpanzee proteins [31] have accumulated over the past ∼14 million years. If 10% of these have been adaptive, then we can estimate that ∼7×10^3^ adaptive amino acid substitutions have taken place over ∼14 million years. Assuming a constant rate of adaptation, this translates into ∼100 adaptive amino acid substitutions that occurred during the past *N_e_* generations (*N_e_* = ∼2×10^5^ years) [59]. Moreover, if regulatory adaptations are common as well, then hundreds of recent selective sweeps should be detectable in the human polymorphism data.

With these considerations in mind, we analyze genomic patterns of nucleotide polymorphism, recombination, functional density and functional divergence in humans using two independent, genome-wide SNP datasets. Consistent with the expectations of positive selection, we detect a positive correlation between levels of neutral polymorphism and recombination rate and a negative correlation between levels of nucleotide polymorphism and both functional density and functional divergence. These correlations remain intact after controlling for a number of possible covariates. The evidence is consistent with positive selection in both regulatory and protein-coding regions. We consider alternative explanations for these findings and argue that, in addition to recurrent selective sweep, only background selection (BS) (loss of neutral variants due to hitchhiking with linked deleterious mutations) can possibly generate most of these patterns. Hitchhiking of neutral polymorphisms with linked selected variants—either due to recurrent positive selection or background selection or possibly both—appears to be a substantial force determining levels of neutral polymorphism in the human genome.

## Results

### Neutral Variation in the Human Genome

To study the effects of RSS, we separate the genomic sequences into two mutually exclusive sets of sequences: “functional” (genic and regulatory) and “nonfunctional”. Both sets of sequences are taken only from the internal parts of autosomes; specifically, we remove all sequences located within 10 Mbp of a telomere or a centromere. We further remove all sequences that cannot be aligned with the chimpanzee genome [31]. The functional set is composed of several types of sequences (see Material and Methods). First, it contains all the genic regions, specifically those that (i) encode exons or are located within 1 kb of any predicted exon and (ii) are located within 5 kb from the starting and ending position of transcripts of protein-coding genes. Because many functional, noncoding sequences are located far from genes in the human genome [60]–[62], we also take all the sequences that can be aligned between primates and zebrafish; sequences that can be aligned over such large evolutionary distances are very unlikely to be unconstrained [63] (see Materials and Methods). The nonfunctional set contains all other sequences except for the repetitive sequences that are filtered out using RepeatMasker [64]. We remove repetitive regions because both alignment and SNP discovery are more problematic in such regions [65]. Hereafter, we will refer to the sequences in the primarily nonfunctional set (totaling ∼1,080 Mbp) as “neutral” sequences for brevity.

We use two SNP datasets: (i) ∼1.2 million Perlegen [66] “A” SNPs discovered using Perlegen chip technology [67] in a panel of 71 individuals of mixed ancestry [68] and (ii) ∼2.0 million SNPs discovered in the diploid sequence of James Watson [69] (see Materials and Methods). In the remainder of the paper, we show the results derived from the analysis of the Perlegen dataset. The results derived from the analysis of the Watson SNPs are shown in the Supplementary Materials. All of the conclusions in the paper are supported by the analysis of either dataset.

We measure the level of neutral nucleotide variation in a genomic window using the number of SNPs within the neutral regions divided by the total number of neutral sites (*θ_neu_*) in a window (see Materials and Methods). This measure is proportional to the conventional Watterson's *θ* [70]. In the remainder of the paper, all measurements are carried out over 400 kb windows. We have also carried out all of the analyses with two other window sizes, 200 and 600 kb; none of the conclusions change depending on the window size (Table S1, S2 and S3, Figures S7, S8, S9, and S10).

The level of neutral polymorphism (*θ_neu_*) depends both on the average time to coalescence within a particular genomic region and on the local constraint and mutation rate. For the purposes of detecting signatures of RSS, variation in constraint and mutation rate generates noise. We assess variability in constraint and mutation rate by measuring divergence per neutral site (*d_neu_*) within the neutral regions between the human and chimpanzee genomes (see Materials and Methods). We detect a positive correlation between *d_neu_* and *θ_neu_* (Table 1), confirming that, as expected, constraint and/or mutation rate vary across the human genome. We control for the variation in neutral mutation rate either by carrying out partial correlations with *d_neu_* or by using a normalized measure of neutral variation, 
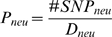
, where #SNP_neu_ stands for the number of SNPs found in the neutral regions and *D_neu_* stands for the number of divergent sites within neutral regions between humans and chimpanzee genomes. *P_neu_* and *θ_neu_* also correlate significantly with repeat density (RD) and GC content (GC) (Table 1, Table S1). Finally, in the case of the Watson data, we further carry out controls for the depth of sequence coverage (Table S4).

**Table 1:** Correlation coefficients among the studied variables: the level of neutral polymorphism (*θ_neu_*), the level of normalized neutral polymorphism (*P_neu_* = *θ_neu_*/*d_neu_*), recombination rate (RR), GC content (GC), the density of simple repeats (RD), the divergence at coding sites (*D_n_*), the divergence at conserved noncoding region (*D_x_*), the number of codons (*FD_n_*), the number of conserved noncoding sites (*FD_x_*), and the level of neutral divergence (*d_neu_*).

	*θ_neu_*	*P_neu_*	RR	GC	RD	*D_n_*	*D_x_*	*FD_n_*	*FD_x_*	*d_neu_*
*θ_neu_*	—	0.9364**	0.2187**	−0.2747**	−0.1046**	−0.2939**	−0.1655**	−0.3210**	−0.3094**	0.2868**
*P_neu_*	0.7880**	—	0.1309**	−0.2460**	−0.1306**	−0.2467**	−0.1552**	−0.2363**	−0.2161**	−0.0166^NS^
										^(1.27e-2)^
RR	0.1486**	0.0886**	—	0.3535**	−0.2769**	0.0480*	−0.0454*	0.0267*	−0.0243**	0.2934**
						^(5.36e-13)^	^(8.58e-12)^	^(6.04e-5)^	^(2.57e-4)^	
GC	−0.1837**	−0.1630**	0.2421**	—	−0.0617**	0.5694**	0.1899**	0.6100**	0.5096**	−0.1322**
RD	−0.0703**	−0.0878**	−0.1876**	−0.0412*	—	0.0226*	0.0617**	−0.0248*	−0.0356*	0.0539*
				^(1.63e-20)^		^(6.81e-4)^		^(1.93e-4)^	^(8.86e-8)^	^(5.55e-16)^
D_n_	−0.2079**	−0.1733**	0.0337*	0.4141**	0.0166*	—	0.3027**	0.8941**	0.6772**	−0.1727**
			^(2.81e-13)^		^(3.17e-4)^					
D_x_	−0.1150**	−0.1080**	−0.0313*	0.1296**	0.0425*	0.2204**	—	0.3008**	0.4965**	−0.0444*
			^(8.27e-12)^		^(1.81e-20)^					^(2.53e-11)^
FD_n_	−0.2213**	−0.1606**	0.0188*	0.4397**	−0.0163*	0.7379**	0.2119**	—	0.8260**	−0.3022**
			^(3.03e-5)^		^(3.08e-4)^					
*FD_x_*	−0.2096**	−0.1446**	−0.0157*	0.3535**	−0.0238*	0.5045**	0.3524**	0.6493**	—	−0.3242**
			^(4.00e-4)^		^(7.89e-8)^					
d_neu_	0.2011**	−0.0109^NS^	0.2011**	−0.0917**	0.0365*	−0.1264**	−0.0320*	−0.2115**	−0.2226**	—
		^(1.38e-2)^			^(2.12e-16)^		^(2.96e-12)^			

**: *P*<1e-20.

*: 1e-20≤*P*<1e-3.

NS *P*> = 1e-3.

Spearman's ρ and Kendall's τ are given at the upper and lower diagonal parts of the table, respectively. *P*-values are given in parentheses for marginally significant (1e-20≤*P*<1e-3) and nonsignificant (NS, *P*> = 1e-3) values.

### Positive Correlation between Levels of Neutral Polymorphism and Recombination Rate

The overall effect of RSS on the regional levels of neutral polymorphism should depend on (i) the regional rate of recombination, (ii) the number of recent sweeps (the rate of RSS), and (iii) the strength of positive natural selection associated with a typical adaptive substitution (the strength of RSS). The levels of neutral polymorphism across the genome should correlate positively with the rate of recombination and negatively with the rate and the strength of RSS.

**Figure 1:**
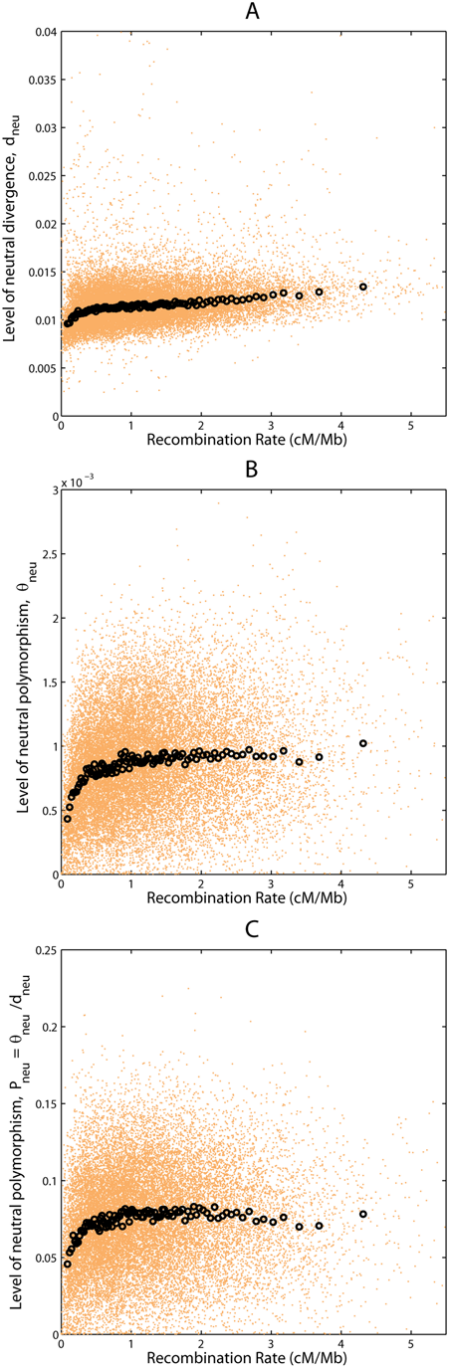
Correlations between recombination rate and neutral divergence rate and neutral polymorphism. Scatter plots display values of two variables in orange dots for (A) recombination rate and the level of neutral divergence rate (*d_neu_*), (B) recombination rate and the level of neutral polymorphism (*θ_neu_*), and (C) recombination rate and the level of normalized neutral polymorphism (*P_neu_* = *θ_neu_*/*d_neu_*). Black circles are average values for orange dots pooled in 100 bins each containing 1% of the data points.

We take estimates of recombination rate from Myers et al. [71], who used a statistical approach to infer recombination rates from linkage disequilibrium data in humans; these rates have been shown to be highly reliable by comparison to pedigree data [72]. The levels of neutral polymorphism measured by both *θ_neu_* and *P_neu_* increase with the recombination rate (Figures 1, S1, and S2). The correlation remains when we control for possible confounders such GC content (GC), repeat density (RD), and divergence at neutral sites (*d_neu_*) separately (Table 2S) or together (Pearson r (*θ_neu_*, RR|GC, RD, *d_neu_*) = 0.254, Pearson r (*P_neu_*, RR|GC, RD) = 0.209, *P*<0.001 in both cases).

### Lower Levels of Neutral Polymorphism in the Functionally Dense Regions

Under a model of RSS regions experiencing more frequent or stronger selective sweeps should show lower levels of neutral polymorphism. Because positive selection should be more prevalent in regions of greater functional density, RSS is expected to generate a negative correlation between the degree of functional density and the level of neutral polymorphism. We measure functional density in two complementary ways. First, in each 400 kb window, we count the number of protein-coding codons (*FD_n_*) as a proxy of protein-coding density. In addition, we count the number of nongenic sites that can be aligned between primates and zebrafish (*FD_x_*) as a proxy of the number of conserved noncoding sites (CNRs) (see Materials and Methods for details).

**Figure 2:**
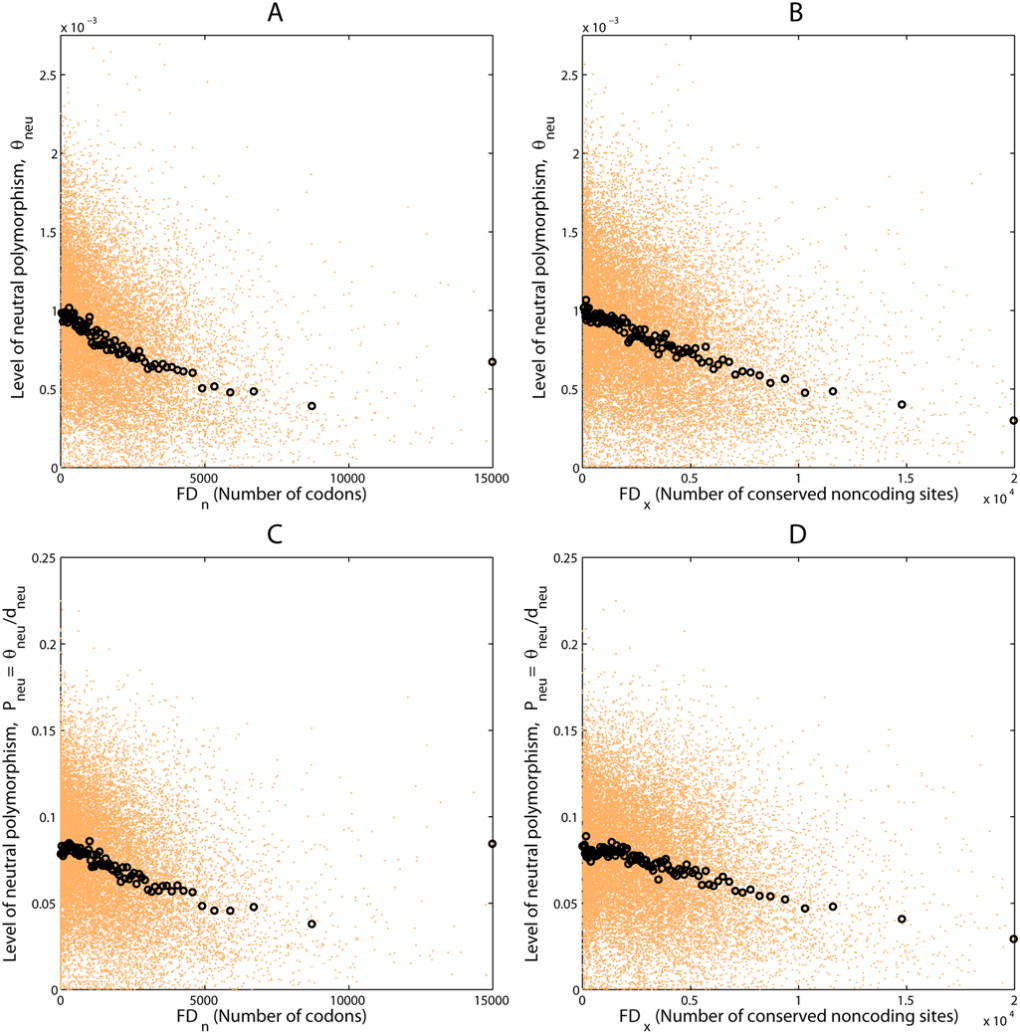
Relationships among the levels of functional density and neutral polymorphism. Scatter plots display values of two variables in orange dots for (A) the number of codons (*FD_n_*) and the level of neutral polymorphism (*θ_neu_*), (B) the number of conserved noncoding sites (*FD_x_*) and the level of neutral polymorphism (*θ_neu_*), (C) the number of codons (*FD_n_*) and the level of normalized neutral polymorphism (*P_neu_* = *θ_neu_*/*d_neu_*), and (D) the number of conserved noncoding sites (*FD_x_*) and the level of normalized neutral polymorphism (*P_neu_* = *θ_neu_*/*d_neu_*). Black circles are average values for orange dots pooled in 100 bins each containing 1% of the data points.

Consistent with the predictions under RSS, there are strongly negative correlations between either measure of functional density (*FD_n_*,*FD_x_*) and measures of neutral variability (Figures 2, S3, S4, and Tables 2, S3). After controlling for GC content (GC), recombination rate (RR), repeat density (RD), and divergence at putatively neutral sites (*d_neu_*) (in the case of *θ_neu_*) the correlations become substantially weaker but do remain statistically significant (Tables 2, 3S). The correlations between *FD_n_* and both *θ_neu_* and *P_neu_* remain significant after we control for *FD_x_*; and similarly, the correlations between *FD_x_* and both *θ_neu_* and *P_neu_* are still significant when we control for *FD_n_* (Tables 2, 3S).

**Table 2:** Spearman rank correlation and partials correlation coefficients between the number of codons (*FD_n_*) and the levels of neutral polymorphism (*θ_neu_*) or the normalized neutral polymorphism (*P_neu_* = *θ_neu_*/*d_neu_*), and between the number of conserved noncoding sites (*FD_x_*) and the levels of neutral polymorphism (*θ_neu_*) or normalized neutral polymorphism (*P_neu_* = *θ_neu_*/*d_neu_*).

*FD_n_ vs θ_neu_*	*FD_n_ vs P_neu_*	*FD_x_ vs θ_neu_*	*FD_x_ vs P_neu_*	RR, GC, RD	*D_n_, D_x_*	*FD_n_ (FD_x_§)*	*d_neu_*
**−0.321** **	**−0.236** **	**−0.309** **	**−0.216** **	○	○	○	○
**−0.126** **	**−0.060** **	**−0.130** **	**−0.059** **	•	○	○	○
**−0.096** **	—†	**−0.099** **	—†	•	○	○	•
−0.036*	−0.025* †	−0.042*	−0.021† *	•	○	•§	•(○)†
^(6.67e-8)^	^(1.94e-4)^	^(2.34e-10)^	^(1.35e-3)^				
0.010^NS^	0.027† *	−0.025*	0.007† NS	•	•	•§	•(○)†
^(1.51e-1)^	^(4.80e-5)^	^(1.42e-4)^	^(3.16e-1)^				

**§:** Correlation coefficients for *FD_n_* versus *θ_neu_* or *P_neu_* was calculated here controlling for *FD_x_* and the correlation coefficients for *FD_x_* versus *θ_neu_* or *P_neu_* was calculated here controlling for *FD_n._*

**†:** Correlation coefficients for *FD_n_* or *FD_x_* versus *P_neu_* were not calculated or were calculated without controlling for *d_neu_*, as *P_neu_* ( = *θ_neu_* /*d_neu_*) is not independent from *d_neu_*.

**: *P*<1e-10.

*: 1e-10≤*P*<1e-3.

NS *P*> = 1e-3.

Closed circles (•) indicate the controlled variables. Highly significant values (P<1e-10) are in bold. *P*-values are given in parentheses for marginally significant (1e-10≤*P*<1e-3) and nonsignificant (NS, *P*> = 1e-3) values.

### Lower Levels of Neutral Polymorphism in the Regions of Higher Functional Divergence

**Figure 3:**
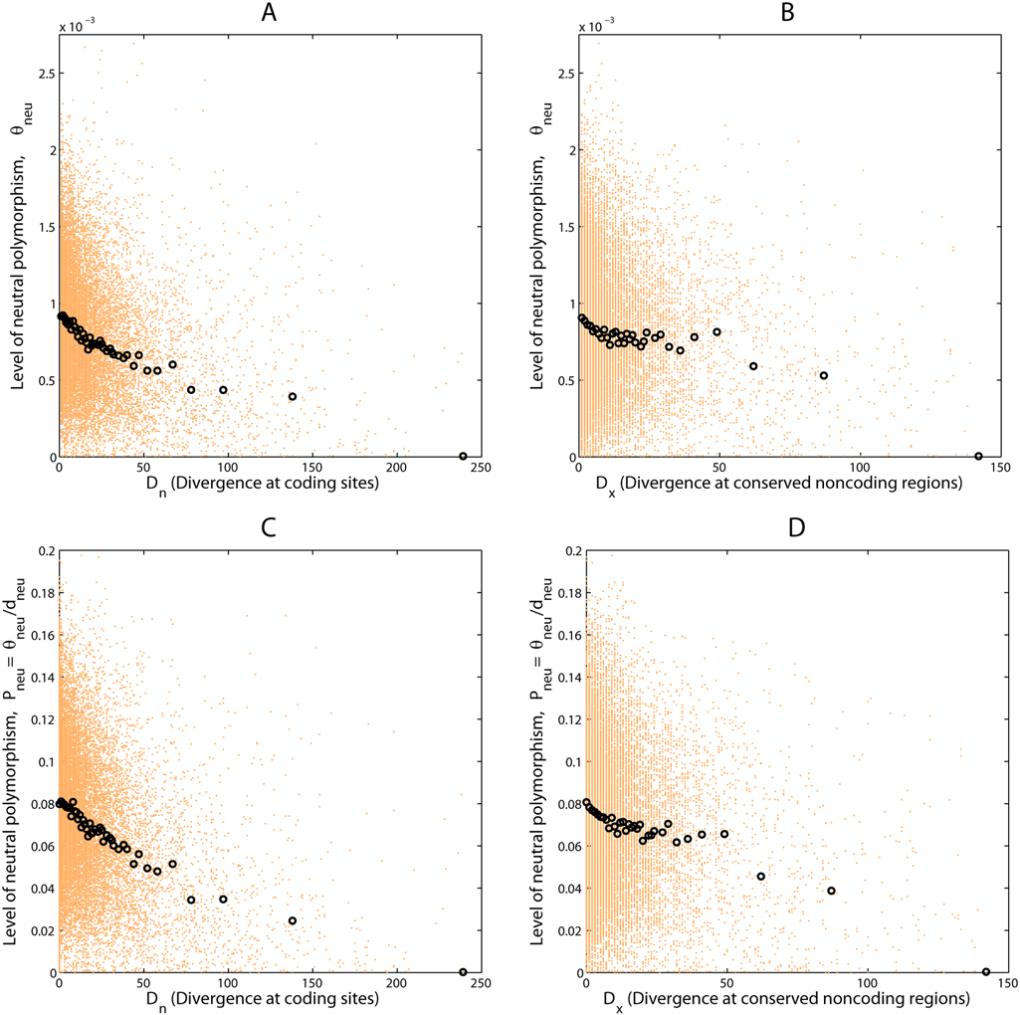
Relationships among the levels of functional divergence and neutral polymorphism. Scatter plots display values of two variables in orange dots for (A) the divergence at coding sites (*D_n_*) and the level of neutral polymorphism (*θ_neu_*), (B) the divergence at conserved noncoding region (*D_x_*) and the level of neutral polymorphism (*θ_neu_*), (C) the divergence at coding sites (*D_n_*) and the level of normalized neutral polymorphism (*P_neu_* = *θ_neu_*/*d_neu_*), and (D) the divergence at conserved noncoding region (*D_x_*) and the level of normalized neutral polymorphism (*P_neu_* = *θ_neu_*/*d_neu_*). Black circles are average values for orange dots pooled in 100 bins each containing 1% of the data points.

The number of differences between humans and chimpanzee genomes at functional regions is likely to be a more direct proxy of the rate of positive selection than the functional density. Consistent with the expectations of RSS, we detect lower levels of *θ_neu_* (*P_neu_*) in regions of higher *D_n_* (the count of divergent amino acid coding sites) or *D_x_* (the count of divergent sites within conserved noncoding regions) (Tables 3, S3, Figures 3, S5, S6). These correlations remain significant when we control for GC content (GC), recombination rate (RR), repeat density (RD), and functional density (*FD_n_*, *FD_x_*, or both) (Tables 3, S4). The correlations between either *D_n_* or *D_x_* and either of the two measures of neutral variation (*θ_neu_* or *P_neu_*) remain statistically significant when we control/correct for the other measure of functional divergence (i.e. control for *D_n_* in the case of correlations of neutral diversity with *D_x_* and, similarly, control for *D_x_* in the case of correlations of neutral diversity with *D_n_*) (Tables 3, S4).

**Table 3:** Spearman rank correlation and partials correlation coefficients between the divergence at coding sites (*D_n_*) and the levels of neutral polymorphism (*θ_neu_*) or normalized neutral polymorphism (*P_neu_* = *θ_neu_*/*d_neu_*) and between the divergence at conserved noncoding region (*D_x_*) and the levels of neutral polymorphism (*θ_neu_*) or normalized neutral polymorphism (*P_neu_* = *θ_neu_*/*d_neu_*).

*D_n_ vs θ_neu_*	*D_n_ vs P_neu_*	*D_x_ vs θ_neu_*	*D_x_ vs P_neu_*	RR, GC, RD	*FD_n_, FD_x_*	*D_n_ (D_x_§)*	*d_neu_*
**−0.294** **	**−0.247** **	**−0.165** **	**−0.155** **	○	○	○	○
**−0.113** **	**−0.089** **	**−0.082** **	**−0.084** **	•	○	○	○
**−0.105** **	—†	**−0.160** **	—†	•	○	○	•
**−0.089** **	**−0.073** ** †	**−0.064** **	**−0.066** ** †	•	○	•§	•(○)†
**−0.047** **	**−0.065** ** †	**−**0.042*	**−0.056** ** †	•	•	•§	•(○)†
		^(2.06e-10)^					

**§:** Correlation coefficients for *D_n_* versus *θ_neu_* or *P_neu_* were calculated controlling for *D_x_* and the correlation coefficients for *D_x_* versus *θ_neu_* or *P_neu_* were calculated controlling for *D_n_*.

**†:** Correlation coefficients for *D_n_* or *D_x_* versus *P_neu_* were not calculated or were calculated without controlling for *d_neu_*, as *P_neu_* ( = *θ_neu_* /*d_neu_*) is not independent of *d_neu_*.

**: *P*<1e-10.

*: 1e-10≤*P*<1e-3.

NS *P*> = 1e-3.

Closed circles (•) indicate the controlled variables. Highly significant values (P<1e-10) are in bold. *P*-values are given in parentheses for marginally significant (1e-10≤*P*<1e-3) value.

## Discussion

The genome-wide patterns of nucleotide polymorphism in the human genome contain much information about the historical patterns of mutation, recombination, natural selection and population histories of modern humans. Here we search for traces of recurrent positive selection in the patterns of diversity at (mostly) neutral sites across the human genome. A number of studies argued that positive selection is reasonably common in humans [15],[30],[31],[33], although substantially less common than in Drosophila [10], [11], [16], [23]–[25],[29] and *E. coli* [26]. A recent study estimated that ∼10% of all amino acid substitutions between humans and chimpanzees have been driven by positive selection [21]. If true, then signatures of hundreds of recent selective sweeps should still be detectable in the pattern of neutral variation in the human genome.

Because recurrent adaptive substitutions leave local (on the order of 0.1 s/*ρ*) and transient (on the order of *N_e_* generations) dips in neutral polymorphism, persistent adaptation should lead to lower levels of neutral polymorphism in regions of lower recombination and regions where selective sweeps are more frequent and/or stronger on average. Here we have confirmed these predictions by showing that levels of SNP density are lower in the regions of lower recombination and in the regions of higher functional density and functional divergence.

In addition to RSS, a number of other evolutionary forces can generate heterogeneous patterns of polymorphism: (i) variation in mutation rates and selective constraint, (ii) demographic events such as population structure, bottlenecks, and fast recent population growth, and (iii) hitchhiking of neutral variants with recurrent deleterious mutations (background selection (BS)). In addition, uneven ascertainment of SNPs across the genome could generate spurious variability in SNP density. Below we discuss the evidence in relation to these alternative possibilities and argue that hitchhiking—due to selective sweeps or background selection—needs to be invoked to explain the detected patterns.

### Ascertainment Biases

All SNP datasets suffer from ascertainment biases during the SNP discovery phase that can systematically under- or overestimate numbers of SNPs in particular genomic regions or at particular types of sites. We address this concern by using two very different SNP datasets that are likely to have different ascertainment biases: (i) the high quality (type A) SNPs from the Perlegen dataset [66] and (ii) SNPs discovered in the sequenced diploid genome of James Watson [69]. The type A SNPs were discovered using Perlegen oligo hybridization chip technology in a panel of 71 individuals of mixed ancestry [66]. This set is biased against SNPs located in repetitive regions, given that it is difficult to design uniquely hybridizing oligonucleotides in such repetitive regions [66]. The diploid genome of James Watson was sequenced using the 454 technology and does not suffer from the same technological problems as the Perlegen oligonucleotide chip hybridization technology.

We obtain very similar results using both datasets, which argues that it is unlikely that specific ascertainment biases are responsible for the observed patterns. In addition, we also used the density of the repeats, GC content and functional density as variables in our statistical analyses and showed that all of the signatures of genetic hitchhiking in our data are robust to statistical controls for these variables. The depth of coverage in the Watson sequencing data also does not noticeably affect any of the detected correlations (Table S4).

### Noise in Polymorphism due to Demographic Phenomena

The demographic history of human populations in general, and specifically of the populations that have been used for SNP discovery and SNP typing in the Perlegen data, is very complex. Bottlenecks, quick population growth and complex patterns of admixture (for example in the African–American population) are expected to perturb levels of neutral polymorphism across the genome. Collectively, we will denote these forces as “demography”.

The effects of recent demography undoubtedly generate much variation in neutral polymorphism; however, the correlations that we observe are likely to be weakened and unlikely to be generated by the demographic processes alone. For instance, the lower levels of neutral polymorphism in the regions that have large numbers of the protein-coding (*D_n_*) and functional noncoding (*D_x_*) differences are hard to explain by demography; demographic events cannot easily affect the longer-term rates of functional divergence that have been accumulating for ∼10–14 million years between chimpanzees and humans [73]. On the other hand, it is clear that demography needs to be taken into account in order to use the detected signatures to evaluate the strength of hitchhiking in the human genome.

### Variation in the Rate of Mutation and Selective Constraint

Some of the variation in levels of polymorphism in the sequences that we use to measure levels of neutral polymorphism could be due to the variability in the rates of mutation and levels of selective constraint. We measure levels of neutral variation in the sequences that are less likely to be under selective constraint: they are noncoding, located far from exons, and cannot be aligned with distantly related species such as zebrafish. Nevertheless, some residual variation in constraint is likely to remain. Indeed, the positive correlation between our measures of the levels of neutral polymorphism (*θ_neu_*) and divergence (*d_neu_)* (Table 1 and 1S) suggests that mutation rates and/or levels of constraint vary systematically in these regions. It is therefore important to control for the variation in the levels of selective constraint and mutation rate; we do so by using the levels of divergence (*d_neu_*) as a variable in partial correlation analyses or by using the measure *P_neu_* (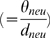).The levels of neutral variation correlate strongly with recombination rate, functional density and functional divergence after controlling for neutral divergence suggesting that these correlations are not due to the variation in mutation rate or constraint

Partial correlations may not remove all of the effects of the variation in mutation rate and constraint, however. The variation in selective constraint among neutral regions should have a stronger effect on the levels of neutral divergence (*d_neu_*) than on the levels of neutral polymorphism (*θ_neu_*) because deleterious mutations have a greater chance of segregating in the population than to become fixed. This implies that if the negative correlation between *θ_neu_* and levels of functional density were entirely due to the variation in selective constraint (specifically higher remaining constraint in regions of higher functional density), then controlling for divergence (*d_neu_*) should make the partial correlation between neutral polymorphism (*θ_neu_*) and functional density positive. Yet we see the opposite: the correlations between *P_neu_* and functional density and the partial correlation between *θ_neu_* and functional density with respect to *d_neu_* both remain strongly negative. This suggests that the variation in selective constraint is unlikely to generate the correlations between levels of neutral variation and recombination rate, functional density and functional divergence that we see in this study.

On the other hand, variability in mutation rates might contribute to some of the observed patterns. Specifically, the positive correlation between neutral diversity and rates of recombination could be due to the mutagenic effects of recombination. Because rates of recombination at local scales (although not necessarily at the 200–600 kb scales relevant to this study) evolve fast [55], [56], [74]–[77], mutagenic effects of recombination should have more pronounced effects on the levels of polymorphism than on the levels of divergence. If so, controlling for neutral divergence (*d_neu_*) may not entirely account for the higher mutation rates produced by recent recombination [55].

Mutagenic effects of recombination are expected to affect levels of polymorphism proportionately to the rate of recombination in the area, whereas hitchhiking (RSS or BS) is expected to affect levels polymorphism in regions of very low recombination much more substantially [78]. We observe a mostly linear effect of recombination on divergence (*d_neu_*) suggestive of the mutagenic effect of recombination and further arguing that the regional recombination rates at the level of our analysis (200 to 600 kb) do not evolve as fast as the location of recombination hotspots. In contrast, the effect of recombination on the levels of polymorphism (*θ_neu_* and *P_neu_*) is curvilinear, with most of the effect limited to the regions of the lowest recombination rates (Figure 1 and S1). Indeed, when we split the data by the median value of recombination rate (RR = 1.040 cM/MBp), the correlation between the levels of neutral divergence (*d_neu_*) and recombination rate (RR) for the two halves of the data are of similar strength (r (*d_neu_*, RR|RR<1.040) = 0.197 and r (*d_neu_*, RR|RR>1.040) = 0.220). However, the correlations between recombination rate and levels of polymorphism (*θ_neu_* or *P_neu_*) are much stronger in the low recombination regions than in the high recombination regions ((r (*θ_neu_*, RR|RR<1.040) = 0.249 versus r (*θ_neu_*, RR|RR>1.040) = 0.045; r (*P_neu_*, RR|RR<1.040) = 0.194 versus r (*P_neu_*, RR|RR>1.040) = −0.0241). These considerations suggest that most of the positive correlation between recombination rates and levels of neutral polymorphism, and especially the reduction at lower recombination rates, is caused by some form of hitchhiking. These results are consistent with the findings of Hellman et al [58] who detected lower levels of polymorphism in the areas of low recombination close to centromeres and telomeres. Note that in our study we explicitly excluded telomeric and centromeric regions (see Materials and Methods), making our findings complementary to those of Hellman et al [58].

### Effects due to Background Selection

Background selection (BS) is the process of hitchhiking of neutral or weakly deleterious polymorphism with linked strongly deleterious polymorphisms [79]–[82]. BS should be more efficacious and lead to lower levels of neutral polymorphism in regions of lower recombination. It is thus quite possible that the positive correlation between neutral polymorphism and recombination rate is due in part to BS. In addition, BS should be stronger in the more constrained genomic regions because such regions should experience higher rates of deleterious mutation (e.g. [58]). Therefore BS is likely to contribute to the negative correlation between levels of neutral polymorphism and functional density as well. Because regions of higher functional density also exhibit higher rates of functional divergence (Tables 1 and S1), BS could contribute to the negative correlation between levels of neutral polymorphism and functional divergence as well.

It is less clear whether BS could generate the negative correlation between the levels of neutral polymorphism and functional divergence after controlling for levels of functional density (Tables 3, S4, Figure S6). Two regions of equal functional density can differ in the strength of BS if they differ in the rate of deleterious mutations in the functional sequences. The higher level of deleterious mutations should lead to stronger BS and therefore lower levels of polymorphism in the linked neutral sequences. At the same time, the higher rate of deleterious mutations is likely to come at the expense of neutral mutations at functional sites and thus should lead to lower levels of protein and regulatory divergence. The reduction of neutral mutation rate in the regions of higher deleterious mutation should lead to a positive correlation between levels of neutral polymorphism and functional divergence after controlling for functional density—the opposite of what is seen. On the other hand, the increase in the rate of fixation of weakly deleterious mutations, also expected in the regions of stronger BS, counteracts the reduction of the rate of functional divergence due to the reduction of neutral mutation rate. The combined effect is difficult to estimate given that we do not have information about the distribution of the rates of mutations of different selective effects along the genome.

There is another pattern we observed that is not naturally predicted by BS. The correlations between functional density (*FD_n_* or *FD_x_*) and neutral polymorphism weaken very substantially and in some cases become nonsignificant when we control for functional divergence at replacement (*D_n_*) and conserved noncoding sites (*D_x_*) (Table 2). Functional density is likely to be a better of proxy of regional constraint than functional divergence. If BS is indeed the dominant force in the generation of the observed patterns, we might have expected correlations between neutral polymorphisms with *FD_n_* and/or *FD_x_* to be the most robust.

Without a better understanding of the distribution of selective effects and rates of new mutations, we cannot reject the possibility that BS contributes substantially to all of the detected patterns. It appears, however, that only specific distributions of selective effects of new mutations would generate all of the observed patterns. Whether such a distribution exists in principle and whether the distribution of selective effects of human mutation satisfies these requirements in fact remains to be determined.

### The Nature and the Effect of Natural Selection at Linked Sites

The arguments above suggest strongly that some form of hitchhiking, either BS or RSS, needs to be invoked to explain the results presented in this paper. These results also suggest that natural selection at both coding and regulatory sites affect linked neutral polymorphism. This is because the measures of the rate of functional evolution at coding and regulatory sites appear to influence levels of neutral polymorphism independently of each other. Specifically, divergence at coding sites and divergence at regulatory sites correlate negatively with the levels of neutral polymorphism after controlling for each other and for the variation in levels of functional divergence (Table 3, S3). To the extent that this is due to recurrent adaptation selection at both coding and regulatory sites, this would echo results of McDonald-Kreitman analyses of adaptation in Drosophila [12].

Levels of neutral polymorphism correlate stronger with divergence at coding than at non-coding regions, possibly implying that either a higher proportion of nonsynonymous changes are adaptive compared to changes in regulatory regions or that the nonsynonymous adaptations have higher selective coefficients. It is also possible and even likely that *D_x_* is a noisier measure than *D_n_* due to greater difficulties in identification of regulatory regions and the noise in estimating *D_x_* due to misalignments. This pattern may also be due to different rates or distributions of the selective effects of deleterious mutations located in coding and regulatory regions, leading to varying effects of BS on linked neutral polymorphism and functional divergence.

These results can also be used to assess the importance of hitchhiking (either RSS or BS) in affecting patterns of neutral polymorphism. The levels of neutral polymorphism appear to be ∼50% lower in the regions of high *D_n_* or *D_x_* (Figures 3, S5) relative to the regions of zero functional divergence (*D_n_* or *D_x_* = 0). If we assume that this effect is entirely due to hitchhiking, then by using the observed correlation between *θ_neu_* and *D_n_*, we estimate that the levels of polymorphism genome-wide are reduced by 6% genome-wide (Materials and Methods). This reduction is much more pronounced in the more gene-rich regions. For instance, in the 50% of the most gene-rich regions (regions that have greater than the median density of codons (FD_n_)), the neutral polymorphism is reduced by 11%, while in the regions that contain 50% of the genes (regions that have greater than the mean density of codons (FD_n_)), the neutral polymorphism is reduced by 13%.

It is clear that hitchhiking has left a significant imprint on the patterns and levels of neutral variability in the human genome and that the effects of natural selection at linked sites cannot be ignored in the analysis of polymorphism data in humans. The challenge for the future is to use these signatures to answer a number of outstanding questions. What are the selective effects and genomic distributions of adaptive and deleterious changes responsible for RSS and BS? What is the biological nature of these changes? What is the relative importance of RSS and BS? Can we estimate parameters of adaptive evolution in the presence of BS? The availability of whole genome sequences in a large number of humans may provide the necessary data to answer these questions. What is needed now are the models and tools to harness these data to provide a cogent picture of the effects of natural selection on human genome and human evolution.

## Materials and Methods

### SNP Datasets

All analyses have been carried out using two SNP data sets—Perlegen data [66] and Watson data [69]. Perlegen data were downloaded from http://genome.perlegen.com. These data were annotated based on the NCBI build 35 of the human genome sequence. We updated all the genomic positions of the SNPs to match the latest NCBI build 36, according to the rs number of SNPs in the dbSNP build 127. During the processing, 1,361 SNPs were discarded because they could not be uniquely mapped to the human genome. Perlegen data contain three classes of SNPs: (A) array-based genomic resequencing, (B) reliable external SNP collections, and (C) unvalidated, lower confidence sources (see Supplementary text of [66]). We excluded class B and C SNPs and retained 1,235,057 class A SNPs located on autosomes for our analysis. The Watson data were downloaded from http://jimwatsonsequence.cshl.edu/. The genome of James Watson was sequenced at 6× coverage using 454 Life Sciences Technology [69] and matched to the human genome project's published reference sequence [83]. In the Watson DNA sequence, heterozygous sites, in which each site was sequenced multiple times and both forms of the base were found in the diploid genome, were ascertained as SNPs. Homozygous sites of Watson's DNA sequence that have been sequenced multiple times and that differ from the reference sequence of the human genome were also ascertained as SNPs. In total our Watson dataset consisted of 2,020,767 SNPs.

### Neutral Genomic Regions

Whole-genome alignments of human (H), chimpanzee (C), and zebrafish (Z) sequences were obtained from the Ensembl compara database [84] through the Ensembl Application Program Interfaces (APIs). We defined the “neutral” genomic regions of the human genome if the regions were: (1) H-C aligned, (2) not H-C-Z aligned, (3) located at least 5 kb away from the starting and ending position of transcripts of protein-coding genes and at least 1 kb away from any exons, (4) located on autosomes at least 10 Mbp away from the boundaries of centromeres and the ends of telomeres, (5) not located in the simple repetitive regions of the human genome. The chromosomal coordinates of exons, transcripts and simple repeats were obtained from the finished and annotated human chromosome sequence from the Ensembl database (build 36).

### Neutral Divergence and Polymorphism

Neutral divergence was assessed from H-C alignments. The accuracy of estimation of neutral divergence may be influenced by the misaligned sequences. Indeed, we discovered some short (2 kb on average) neutral genomic regions having extremely high levels of divergent sites, which may result from misalignments (data not shown). To minimize the possible influence of misalignments, we only counted “isolated” substitutions that are flanked by two monomorphic positions on each side (*i*.*e*. no substitutions or SNPs were mapped to these sites). We denoted the number of isolated substitutions between human and chimpanzee sequences as *D_neu_*, and the number of isolated substitutions per neutral site, *d_neu_*. To measure neutral polymorphism, we counted the number of SNPs in neutral regions and denoted the number of SNPs per site as *θ_neu_*. Alternatively, we measured neutral polymorphism with 
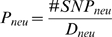
. Data manipulation was done using Matlab functions based on PGEToolbox [85] and MBEToolbox [86].

### Proxies of the Rate of Adaptive Evolution

We used four metrics as proxies of the rate of adaptive evolution for a given region in the human genome. Functional density was measured using *FD_n_*, the number of codons, and *FD_x_*, the number of aligned bases in the H-C-Z three-way alignments. Functional divergence was measured using *D_n_*, the number of codons involved in nonsynonymous substitutions between H-C orthologous gene pairs, and *D_x_*, the number of H-C substitutions in H-C-Z alignments that are located in noncoding human genomic regions. For each pair of genes, the amino-acid sequences were extracted and aligned using CLUSTALW [87] with the default parameters. The corresponding nucleotide sequence alignments were derived by substituting the respective coding sequences from the protein sequences. The synonymous substitution rate (Ks) was then estimated by the maximum-likelihood method implemented in the CODEML program of PAML [88]. Insertions and deletions within alignments were discarded. Poorly aligned orthologous pairs, as indicated by Ks>5, were excluded. The codons containing nonsynonymous substitutions were mapped back onto the human genome and positions were recorded. For simplicity we counted the numbers of codons causing amino-acid changes instead of the numbers of single nucleotide replacement substitutions. In calculation of *D_x_*, we excluded “tri-allelic” sites where the bases of H, C and Z all differ from each other.

### Correlation Analysis

We used 400 kb (as well as 200 and 600 kb) sliding window with a step of 100 kb to scan along the human genome. For each window, two measures of neutral polymorphism (*θ_neu_* and *P_neu_*) and four proxies of the rate adaptive evolution (*FD_n_*, *FD_x_*, *D_n_*, and *D_x_*) were estimated. To reduce noise arising from small sample size, we also discarded the windows with *D_neu_*<500 and the ones with the total amount of “neutral” sequence less than 2 kb. 22,553 400 kb windows have been used for the correlation analysis. Spearman rank correlation or Kendall's correlation coefficients have been calculated in all cases. To visualize correlations between variables, we used scatter plots with regression lines superimposed. We also pooled the data points of neutral polymorphism by the values of the proxy of adaptation under consideration (*e*.*g*. *D_n_*). To do this, we ranked all the data points of the neutral polymorphism by the values of the proxy and then pooled them into 100 bins such that each bin had equal size (*i*.*e*., 1%) of the data points. We then computed average values of the proxies of adaptation and the average value of neutral polymorphism for each bin, and superimposed them onto the scatter plots.

To control for confounding variables, we calculated Spearman partial correlation coefficients between variables X and Y controlling for Z, using the function partialcorr in the Matlab statistic toolbox. Recombination rate estimated by using the coalescent method of [89] were downloaded from http://hapmap.org/downloads/recombination/. The density of simple repeats was computed as the proportion of bases of simple repeats in the given region. Chromosomal coordinates of simple repeats in the human genome, identified by RepeatMasker [64], were obtained from the UCSC genome browser [90].

We also calculated the partial correlation coefficients between variables X and Y by calculating the correlation between the two sets of residuals formed by two linear models X∼Z and Y∼Z (see also [91]) where Z stands for either one or a series of variables. The distribution of *D_n_*, *D_x_*, *FD_n_*, and *FD_x_* values is approximately exponential, which is a problem in a least squares linear model framework in controlling for a third variable, Z. The linear model used to regress out Z is sensitive to the highly non-normal distribution of variables, and the residuals will be highly non-normal, making the results difficult to interpret. Therefore, we quantile-normalized values, replacing the original estimates with their theoretical quantiles based on a normal distribution. Then, we fitted linear models, using as the response variable quantile-normalized *D_n_*, *D_x_*, *FD_n_*, or *FD_x_*, and using as the predictor variables various combinations of recombination rate, GC content, and the density of simple repeats.

Estimation of the effect of hitchhiking on the level of neutral polymorphism was calculated using the regression between *θ_neu_* on *D_n_*, using the formula
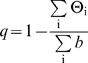
where *q* is the reduction of polymorphism due to hitchhiking, *i* is a window count for the subsets of windows used in the analysis (e.g. *FD_n_*>median (*FD_n_*)), b is the intercept of the regression of *θ_neu_* on *D_n_*.

## Supporting Information

Figure S1 Correlations between recombination rate and neutral divergence rate and neutral polymorphism.
Scatter plots display values of two variables in gray dots for (A) recombination rate (RR) and the level of neutral divergence rate (*d_neu_*), (B) recombination rate (RR) and the level of neutral polymorphism (*θ_neu_*), and (c) recombination rate (RR) and the level of normalized neutral polymorphism (*P_neu_* = *θ_neu_*/*d_neu_*). Red circles are average values for the pooled gray dots in 100 bins each containing 1% of the data points. The solid green line shows the fit of a linear model. Spearman's correlation coefficients for (A) to (C) are 0.302, 0.316, and 0.210, respectively. These coefficients are significantly different from zero (*P*<0.001). The values of *θ_neu_* and *P_neu_* here are based on the Watson data. The results derived from the Perlegen data are given in Figure 1.
(0.1 MB PDF)

Figure S2 Residual-residual plot between recombination rate (RR) and neutral polymorphism [i.e., the level of neutral polymorphism (*θ_neu_*) or the level of normalized neutral polymorphism (*P_neu_* = *θ_neu_*/*d_neu_*)] after statistically removing the effects of GC content (GC), repeat density (RD), functional divergences [i.e., the divergence at coding sites (*D_n_*) and the divergence at conserved noncoding region (*D_x_*)], and functional constraints [i.e., the number of codons (*FD_n_*) and the number of conserved noncoding sites (*FD_x_*)].
*e*(Y|X) is the difference between the observed value of the response variable, Y, and the value suggested by the regression model of Y on several predictor variables X = {GC, RD, *D_n_*, *D_x_*, *FD_n_*, *FD_x_*}. The values of *θ_neu_* and *P_neu_* here are based on the Perlegen data are in (A) and based on the Watson data are in (B).
(0.08 MB PDF)

Figure S3 Relationships among the levels of functional density [i.e., the number of codons (*FD_n_*) or the number of conserved noncoding sites (*FD_x_*)] and neutral polymorphism [i.e., the level of neutral polymorphism (*θ_neu_*) or the level of normalized neutral polymorphism (*P_neu_* = *θ_neu_*/*d_neu_*)].
Scatter plots display values of two variables in gray dots for (A) *FD_n_* and *θ_neu_*, (B) *FD_x_* and *θ_neu_*, (C) *FD_n_* and *P_neu_*, and (D) *FD_x_* and *P_neu_*. Red circles are average values for the pooled gray dots in 100 bins each containing 1% of the data points. The solid, green line shows the fit of a linear model. The values of *θ_neu_* and *P_neu_* here are based on the Watson data. The results derived from the Perlegen data are given in Figure 2.
(0.1 MB PDF)

Figure S4 Residual-residual plots between functional density [i.e., the number of codons (*FD_n_*) or the number of conserved noncoding sites (*FD_x_*)] and neutral polymorphism [i.e., the level of neutral polymorphism (*θ_neu_*) or the level of normalized neutral polymorphism (*P_neu_* = *θ_neu_*/*d_neu_*)], after both have been adjusted for effects of GC content (GC), repeat density (RD), functional divergences [i.e., the divergence at coding sites (*D_n_*) and the divergence at conserved noncoding region (*D_x_*)], and functional density (*FD_n_* or *FD_x_*, excluding the response variable under test).
*e*(Y|X) is the difference between the observed value of the response variable, Y, and the value suggested by the regression model of Y on several predictor variables X = {GC, RD, *D_n_*, *D_x_*, *FD_n_*, *FD_x_*}. The values of *θ_neu_* and *P_neu_* here are based on the Perlegen data are in (A) and based on the Watson data are in (B).
(0.08 MB PDF)

Figure S5 Relationships among the levels of functional divergence [i.e., the divergence at coding sites (*D_n_*) or the divergence at conserved noncoding region (*D_x_*)] and neutral polymorphism [i.e., the level of neutral polymorphism (*θ_neu_*) or the level of normalized neutral polymorphism (*P_neu_* = *θ_neu_*/*d_neu_*)].
Scatter plots display values of two variables in gray dots for (A) *D_n_* and *θ_neu_*, (B) *D_x_* and *θ_neu_*, (C) *D_n_* and *P_neu_*, and (D) *D_x_* and *P_neu_*. Red circles are average values for the pooled gray dots in 100 bins each containing 1% of the data points. The solid, green line shows the fit of a linear model. The values of *θ_neu_* and *P_neu_* here are based on the Watson data. The results derived from the Perlegen data are given in Figure 3.
(0.1 MB PDF)

Figure S6 Residual-residual plots between functional divergence [i.e., the divergence at coding sites (*D_n_*) or the divergence at conserved noncoding region (*D_x_*)] and neutral polymorphism [i.e., the level of neutral polymorphism (*θ_neu_*) and the level of normalized neutral polymorphism (*P_neu_* = *θ_neu_*/*d_neu_*)], after both have been adjusted for effects of GC content (GC), repeat density (RD), functional constraints [i.e., the number of codons (*FD_n_*) and the number of conserved noncoding sites (*FD_x_*)], and functional divergence (*D_n_* or *D_x_*, excluding the response variable under test).
*e*(Y|X) is the difference between the observed value of the response variable, Y, and the value suggested by the regression model of Y on several predictor variables X = {GC, RD, *D_n_*, *D_x_*, *FD_n_*, *FD_x_*}. The values of *θ_neu_* and *P_neu_* here are based on the Perlegen data are in (A) and based on the Watson data are in (B).
(0.08 MB PDF)

Figure S7 Results derived from the sliding windows of 200 kb. Correlations between functional density [i.e., the number of codons (*FD_n_*) or the number of conserved noncoding sites (*FD_x_*)], and divergence [i.e., the divergence at coding sites (*D_n_*) or the divergence at conserved noncoding region (*D_x_*)] and neutral polymorphism [i.e., the level of neutral polymorphism (*θ_neu_*) and the level of normalized neutral polymorphism (*P_neu_* = *θ_neu_*/*d_neu_*)] are given.
The results are based on the Perlegen data.
(0.1 MB PDF)

Figure S8 Results derived from the sliding windows of 200 kb.
Correlations between functional density [i.e., the number of codons (*FD_n_*) or the number of conserved noncoding sites (*FD_x_*)], and divergence [i.e., the divergence at coding sites (*D_n_*) or the divergence at conserved noncoding region (*D_x_*)] and neutral polymorphism [i.e. the level of neutral polymorphism (*θ_neu_*) or the level of normalized neutral polymorphism (*P_neu_* = *θ_neu_*/*d_neu_*)] are given. The results are based on the Watson data.
(0.1 MB PDF)

Figure S9 Results derived from the sliding windows of 600 kb.
Correlations between functional density [i.e. the number of codons (*FD_n_*) or the number of conserved noncoding sites (*FD_x_*)], and divergence [i.e. the divergence at coding sites (*D_n_*) or the divergence at conserved noncoding region (*D_x_*)] and neutral polymorphism [i.e., the level of neutral polymorphism (*θ_neu_*) or the level of normalized neutral polymorphism (*P_neu_* = *θ_neu_*/*d_neu_*)] are given. The results are based on the Perlegen data.
(0.1 MB PDF)

Figure S10 Results derived from the sliding windows of 600 kb.
Correlations between functional density [i.e., the number of codons (*FD_n_*) or the number of conserved noncoding sites (*FD_x_*)], and divergence [i.e., the divergence at coding sites (*D_n_*) or the divergence at conserved noncoding region (*D_x_*)] and neutral polymorphism [i.e., the level of neutral polymorphism (*θ_neu_*) and the level of normalized neutral polymorphism (*P_neu_* = *θ_neu_*/*d_neu_*)] are given. The results are based on the Watson data.
(0.1 MB PDF)

Table S1 Correlation coefficients among the studied variables: the level of neutral polymorphism (*θ_neu_*), he level of normalized neutral polymorphism (*P_neu_* = *θ_neu_*/*d_neu_*), recombination rate (RR), GC content (GC), the density of simple repeats (RD), the depth of sequencing coverage (SC), the divergence at coding sites (*D_n_*), the divergence at conserved noncoding region (*D_x_*), the number of codons (*FD_n_*), the number of conserved noncoding sites (*FD_x_*), and the level of neutral divergence (*d_neu_*).
Spearman's ρ and Kendall's τ are given at the upper and lower diagonal parts of the table, respectively. The values of *θ_neu_* and *P_neu_* are based on the Watson data.
(0.07 MB PDF)

Table S2 Correlation coefficients and partials correlation coefficients between recombination rate (RR) and levels of neutral polymorphism [i.e., the level of neutral polymorphism (*θ_neu_*) and the level of normalized neutral polymorphism (*P_neu_* = *θ_neu_*/*d_neu_*)].
Spearman's partial correlation coefficients were calculated by controlling for all possible combinations of potentially confounding variables. The results of representative combinations are given here. Closed circles (•) indicate the controlled variables. These variables are GC content (GC), the density of simple repeats (RD), the divergence at coding sites (*D_n_*), the divergence at conserved noncoding region (*D_x_*), the number of codons (*FD_n_*), the number of conserved noncoding sites (*FD_x_*), and the level of neutral divergence rate (*d_neu_*). Open circles (○) indicate the variables that were not controlled in a particular analysis. (P) indicates the results are based on the Perlegen data, (W) indicates the results are based on the Watson data. *P*-values are given in parentheses.
(0.1 MB PDF)

Table S3 Spearman rank correlation coefficients and partials correlation coefficients between functional divergence [i.e., the divergence at coding sites (*D_n_*) or the divergence at conserved noncoding region (*D_x_*)] and neutral polymorphism [i.e., the level of neutral polymorphism (*θ_neu_*) and the level of normalized neutral polymorphism (*P_neu_* = *θ_neu_*/*d_neu_*)], and between functional constraints [i.e., the number of codons (*FD_n_*) and the number of conserved noncoding sites (*FD_x_*)] and neutral polymorphism (*θ_neu_* and *P_neu_*).
Spearman's partial correlation coefficients were calculated by controlling for all possible combinations of potentially confounding variables. The results of representative combinations are given here. Closed circles (•) indicate the controlled variables. These variables are GC content (GC), the density of simple repeats (RD), the divergence at coding sites (*D_n_*), the divergence at conserved noncoding region (*D_x_*), the number of codons (*FD_n_*), the number of conserved noncoding sites (*FD_x_*), and the level of neutral divergence rate (*d_neu_*). Open circles (○) indicate the variables that were not controlled in a particular analysis. (P) indicates the results are based on the Perlegen data, (W) indicates the results are based on the Watson data. *P*-values are given in parentheses.
(0.2 MB PDF)

Table S4 Partial Spearman rank correlation coefficients controlling for the depth of sequencing coverage (SC) of Watson data.
Partial Spearman rank correlation coefficients between functional divergence [i.e. the divergence at coding sites (*D_n_*) or the divergence at conserved noncoding region (*D_x_*)] and neutral polymorphism [i.e.,the level of neutral polymorphism (*θ_neu_*) and the level of normalized neutral polymorphism (*P_neu_* = *θ_neu_*/*d_neu_*)], and between functional constraints [i.e.,the number of codons (*FD_n_*) and the number of conserved noncoding sites (*FD_x_*)] and neutral polymorphism (*θ_neu_* and *P_neu_*) are given. Spearman's partial correlation coefficients were calculated by controlling for all possible combinations of potentially confounding variables. The results of representative combinations are given here. Closed circles (•) indicate the controlled variables. These variables are GC content (GC), the density of simple repeats (RD), the depth of sequencing coverage (SC), the divergence at coding sites (*D_n_*), the divergence at conserved noncoding region (*D_x_*), the number of codons (*FD_n_*), the number of conserved noncoding sites (*FD_x_*), and the level of neutral divergence rate (*d_neu_*). Open circles (○) indicate the variables that were not controlled in a particular analysis. (W) indicates the results are based on the Watson data. *P*-values are given in parentheses.
(0.2 MB PDF)
