# Supplementary material for: Pervasive Hitchhiking at Coding and Regulatory Sites in Humans
Source: PLoS Genet. 2009 Jan 16;5(1):e1000336. doi: 10.1371/journal.pgen.1000336 (PMC2613029; doi:10.1371/journal.pgen.1000336)
Supplement: Figure S1 — Correlations between recombination rate and neutral divergence rate and neutral polymorphism. Scatter plots display values of two variables in gray dots for (A) recombination rate (RR) and the level of neutral divergence rate (dneu), (B) recombination rate (RR) and the level of neutral polymorphism (θneu), and (c) recombination rate (RR) and the level of normalized neutral polymorphism (Pneu = θneu/dneu). Red circles are average values for the pooled gray dots in 100 bins each containing 1% of the data points. The solid green line shows the fit of a linear model. Spearman's correlation coefficients for (A) to (C) are 0.302, 0.316, and 0.210, respectively. These coefficients are significantly different from zero (P<0.001). The values of θneu and Pneu here are based on the Watson data. The results derived from the Perlegen data are given in Figure 1. (0.1 MB PDF) [file pgen.1000336.s001.pdf]

Figure S1. Correlations between recombination rate and neutral divergence rate and neutral polymorphism. Scatter plots display values of two variables in gray dots for **(a)** recombination rate (RR) and the level of neutral divergence rate (d_{neu}), **(b)** recombination rate (RR) and the level of neutral polymorphism (θ_{neu}), and **(c)** recombination rate (RR) and the level of normalized neutral polymorphism ($P_{neu}=\theta_{neu}/d_{neu}$). Red circles are average values for the pooled gray dots in 100 bins each containing 1% of the data points. The solid green line shows the fit of a linear model. Spearman's correlation coefficients for **(a)** to **(c)** are 0.302, 0.316, and 0.210, respectively. These coefficients are significantly different from zero (P < 0.001). The values of θ_{neu} and P_{neu} here are based on the Watson data. The results derived from the Perlegen data are given in Fig. 1.

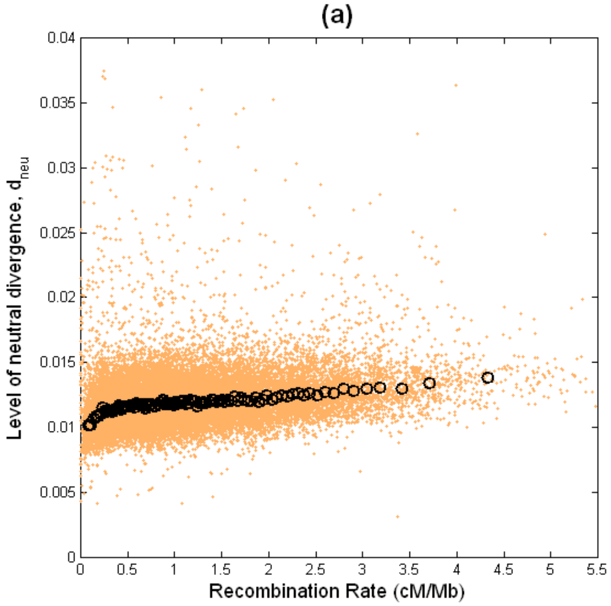

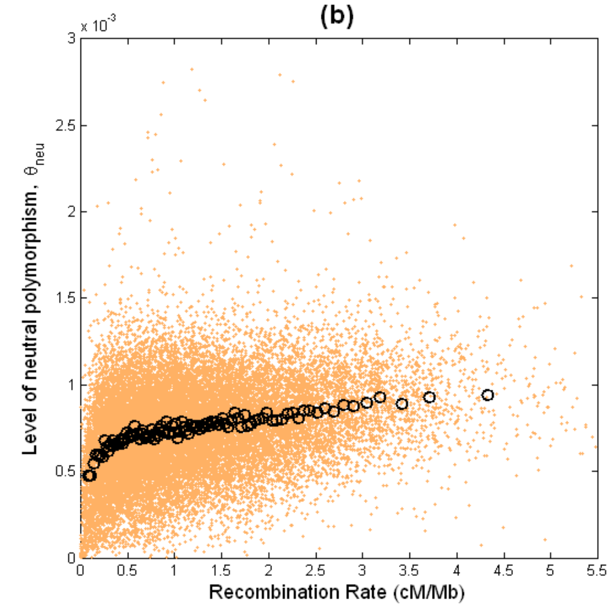

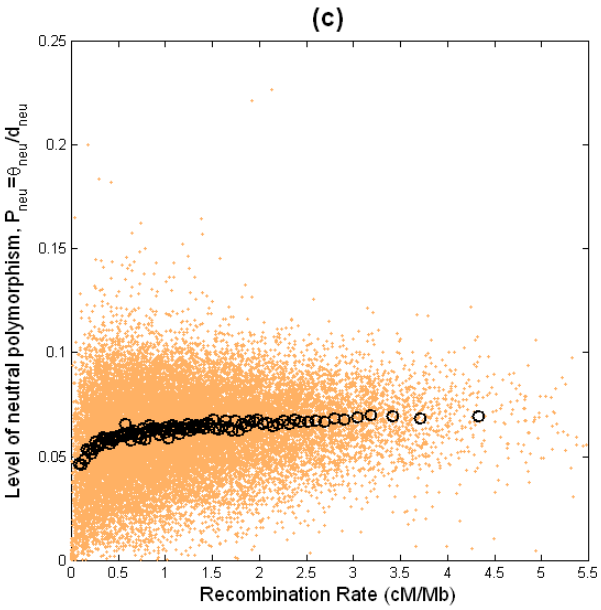
(c)
Level of neutral polymorphism, $P_{neu}=\theta_{neu}/d_{neu}$
Recombination Rate (cM/Mb)
0.25
0.2
0.15
0.1
0.05
0
0
0.5
1
1.5
2
2.5
3
3.5
4
4.5
5
5.5
