# Supplementary material for: Pervasive Hitchhiking at Coding and Regulatory Sites in Humans
Source: PLoS Genet. 2009 Jan 16;5(1):e1000336. doi: 10.1371/journal.pgen.1000336 (PMC2613029; doi:10.1371/journal.pgen.1000336)

Figure S2. Residual-residual plot between recombination rate (RR) and neutral polymorphism [i.e. the level of neutral polymorphism (θ_{neu}) or the level of normalized neutral polymorphism ($P_{neu}=\theta_{neu}/d_{neu}$)] after statistically removing the effects of GC content (GC), repeat density (RD), functional divergences [i.e. the divergence at coding sites (D_n) and the divergence at conserved noncoding region (D_x)], and functional constraints [i.e. the number of codons (FD_n) and the number of conserved noncoding sites (FD_x)]. *e*(Y|X) is the difference between the observed value of the response variable, Y, and the value suggested by the regression model of Y on several predictor variables X={GC, RD, D_n, D_x, FD_n, FD_x}. The values of θ_{neu} and P_{neu} here are based on the Perlegen data are in **(a)** and based on the Watson data are in **(b)**.

(a)

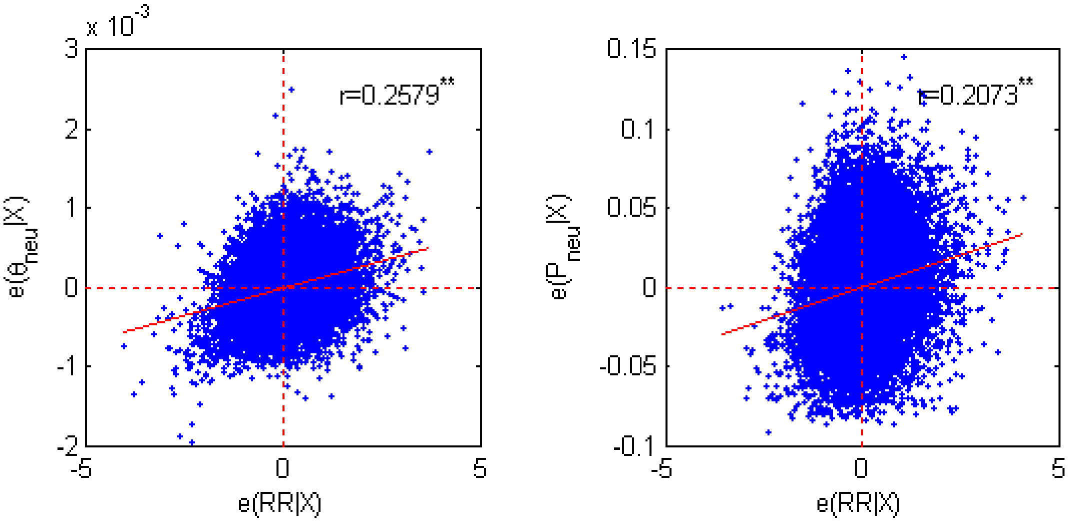

(b)

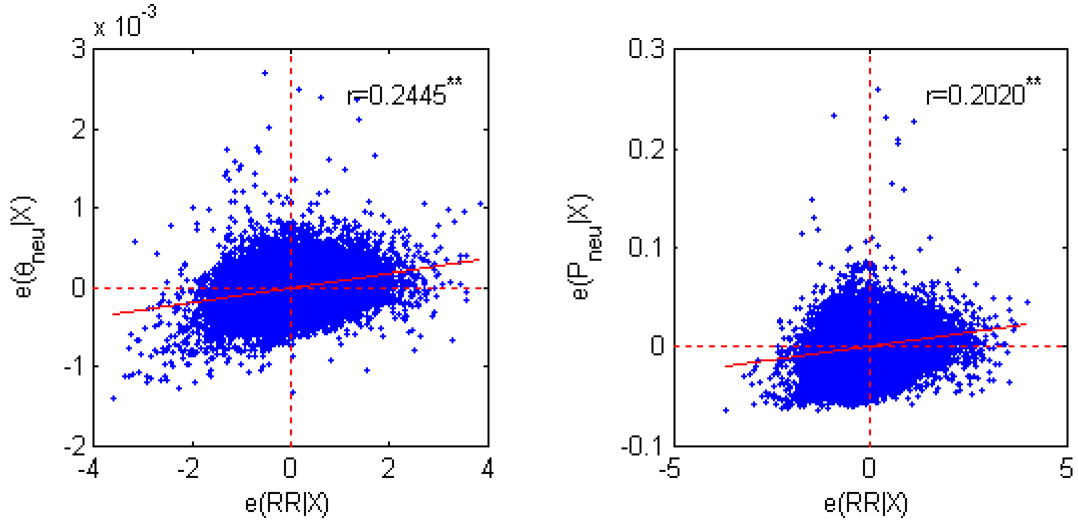

Supplement: Figure S2 — Residual-residual plot between recombination rate (RR) and neutral polymorphism [i.e., the level of neutral polymorphism (θneu) or the level of normalized neutral polymorphism (Pneu = θneu/dneu)] after statistically removing the effects of GC content (GC), repeat density (RD), functional divergences [i.e., the divergence at coding sites (Dn) and the divergence at conserved noncoding region (Dx)], and functional constraints [i.e., the number of codons (FDn) and the number of conserved noncoding sites (FDx)]. e(Y|X) is the difference between the observed value of the response variable, Y, and the value suggested by the regression model of Y on several predictor variables X = {GC, RD, Dn, Dx, FDn, FDx}. The values of θneu and Pneu here are based on the Perlegen data are in (A) and based on the Watson data are in (B). (0.08 MB PDF) [file pgen.1000336.s002.pdf]
