# Supplementary material for: Pervasive Hitchhiking at Coding and Regulatory Sites in Humans
Source: PLoS Genet. 2009 Jan 16;5(1):e1000336. doi: 10.1371/journal.pgen.1000336 (PMC2613029; doi:10.1371/journal.pgen.1000336)
Supplement: Figure S3 — Relationships among the levels of functional density [i.e., the number of codons (FDn) or the number of conserved noncoding sites (FDx)] and neutral polymorphism [i.e., the level of neutral polymorphism (θneu) or the level of normalized neutral polymorphism (Pneu = θneu/dneu)]. Scatter plots display values of two variables in gray dots for (A) FDn and θneu, (B) FDx and θneu, (C) FDn and Pneu, and (D) FDx and Pneu. Red circles are average values for the pooled gray dots in 100 bins each containing 1% of the data points. The solid, green line shows the fit of a linear model. The values of θneu and Pneu here are based on the Watson data. The results derived from the Perlegen data are given in Figure 2. (0.1 MB PDF) [file pgen.1000336.s003.pdf]

Fig S3. Relationships among the levels of functional density [i.e. the number of codons (FD_n) or the number of conserved noncoding sites (FD_x)] and neutral polymorphism [i.e. the level of neutral polymorphism (θ_{neu}) or the level of normalized neutral polymorphism ($P_{neu}=\theta_{neu}/d_{neu}$)]. Scatter plots display values of two variables in gray dots for **(a)** FD_n and θ_{neu}, **(b)** FD_x and θ_{neu}, **(c)** FD_n and P_{neu}, and **(d)** FD_x and P_{neu}. Red circles are average values for the pooled gray dots in 100 bins each containing 1% of the data points. The solid, green line shows the fit of a linear model. The values of θ_{neu} and P_{neu} here are based on the Watson data. The results derived from the Perlegen data are given in Fig. 2.

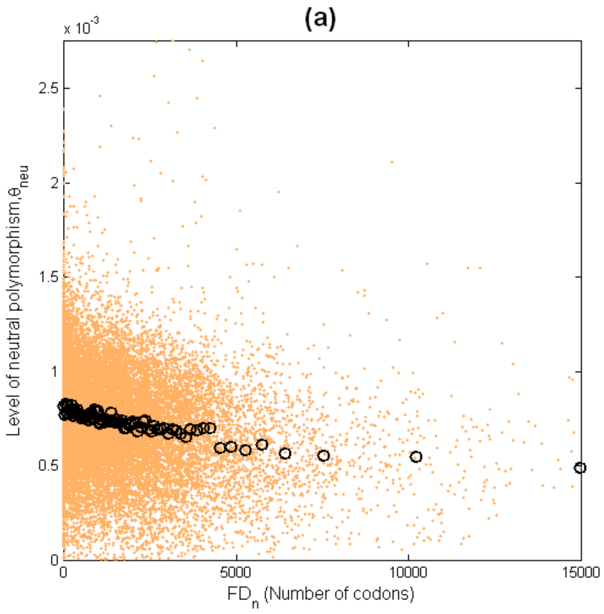

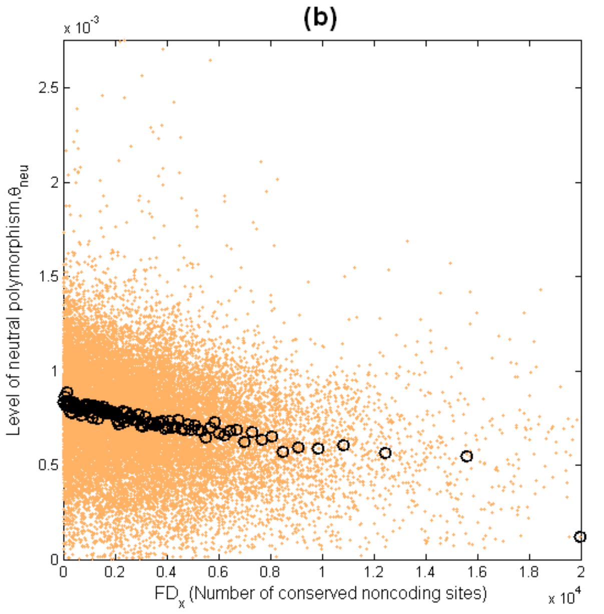

(c)

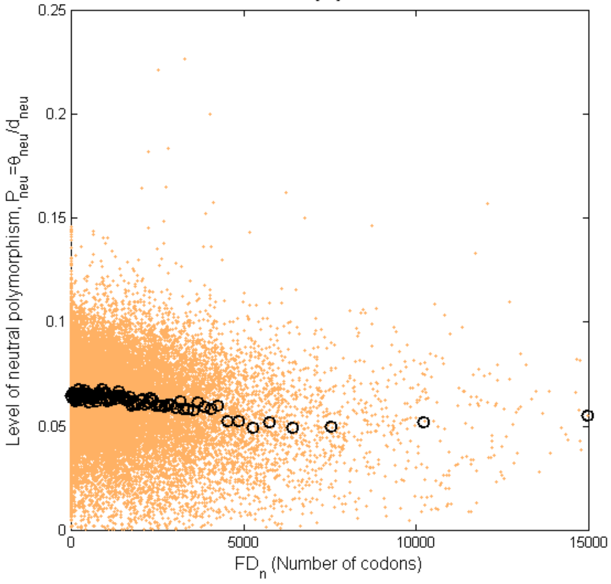
0.25
0.2
0.15
0.1
0.05
0
Level of neutral polymorphism, $P_{neu}=\theta_{neu}/d_{neu}$
0
5000
10000
15000
FD_n (Number of codons)

(d)

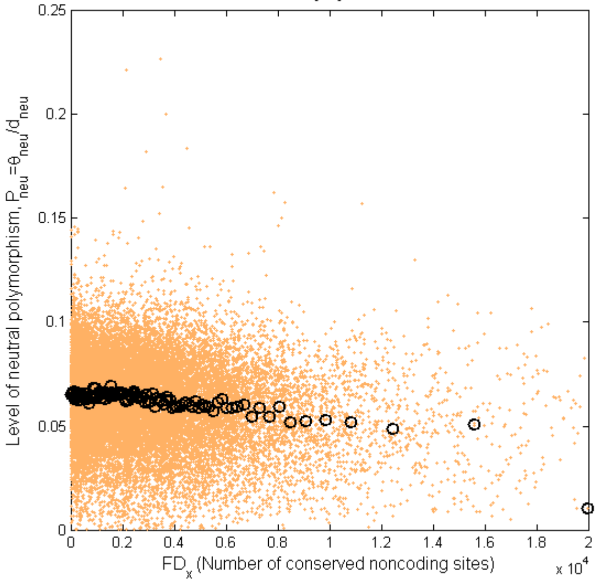
0.25
0.2
0.15
0.1
0.05
0
Level of neutral polymorphism, $P_{neu}=\theta_{neu}/d_{neu}$
0
0.2
0.4
0.6
0.8
1
1.2
1.4
1.6
1.8
2
FD_x (Number of conserved noncoding sites)
$x\ 10^4$
