# Supplementary material for: Pervasive Hitchhiking at Coding and Regulatory Sites in Humans
Source: PLoS Genet. 2009 Jan 16;5(1):e1000336. doi: 10.1371/journal.pgen.1000336 (PMC2613029; doi:10.1371/journal.pgen.1000336)
Supplement: Figure S4 — Residual-residual plots between functional density [i.e., the number of codons (FDn) or the number of conserved noncoding sites (FDx)] and neutral polymorphism [i.e., the level of neutral polymorphism (θneu) or the level of normalized neutral polymorphism (Pneu = θneu/dneu)], after both have been adjusted for effects of GC content (GC), repeat density (RD), functional divergences [i.e., the divergence at coding sites (Dn) and the divergence at conserved noncoding region (Dx)], and functional density (FDn or FDx, excluding the response variable under test). e(Y|X) is the difference between the observed value of the response variable, Y, and the value suggested by the regression model of Y on several predictor variables X = {GC, RD, Dn, Dx, FDn, FDx}. The values of θneu and Pneu here are based on the Perlegen data are in (A) and based on the Watson data are in (B). (0.08 MB PDF) [file pgen.1000336.s004.pdf]

Figure S4. Residual-residual plots between functional density [i.e. the number of codons (*FD_n*) or the number of conserved noncoding sites (*FD_x*)] and neutral polymorphism [i.e. the level of neutral polymorphism (θ_{neu}) or the level of normalized neutral polymorphism ($P_{neu}=\theta_{neu}/d_{neu}$)], after both have been adjusted for effects of GC content (GC), repeat density (RD), functional divergences [i.e. the divergence at coding sites (D_n) and the divergence at conserved noncoding region (D_x)], and functional density (FD_n or FD_x, excluding the response variable under test). *e*(Y|X) is the difference between the observed value of the response variable, Y, and the value suggested by the regression model of Y on several predictor variables X={GC, RD, D_n, D_x, FD_n, FD_x}. The values of θ_{neu} and P_{neu} here are based on the Perlegen data are in **(a)** and based on the Watson data are in **(b)**.

(a)

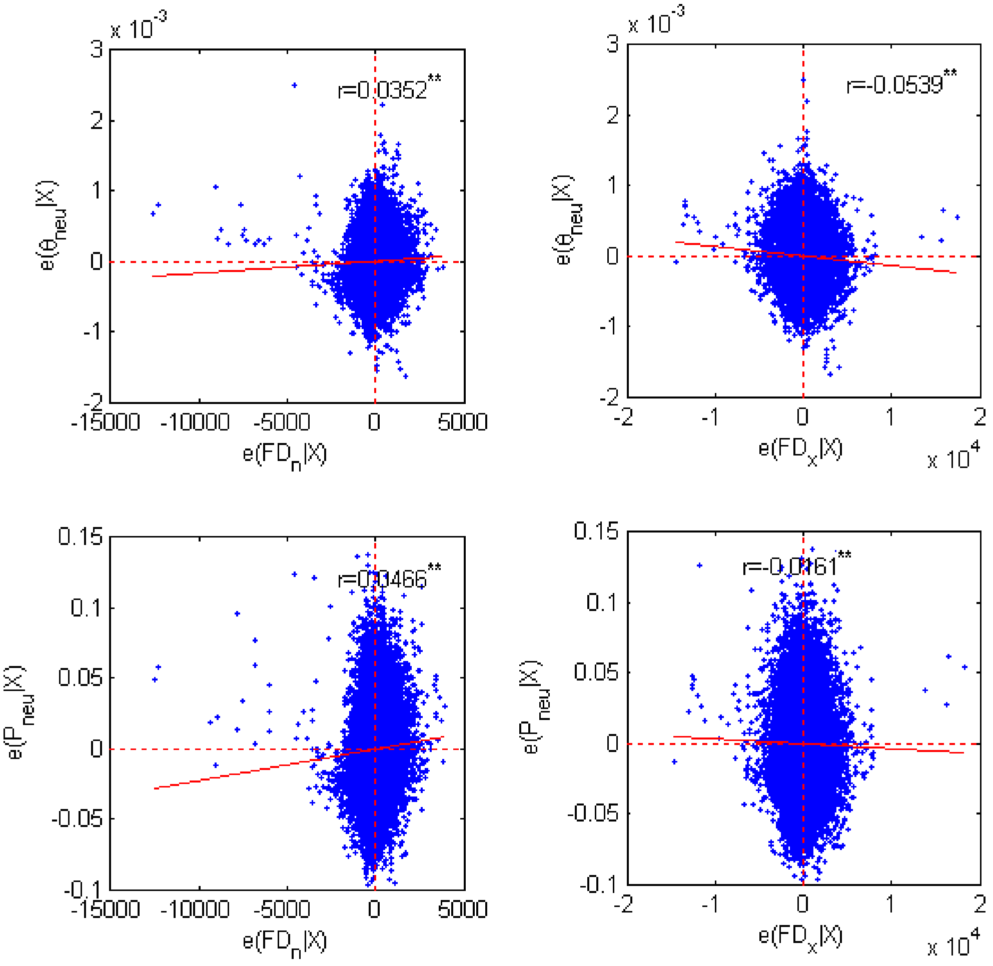

(b)

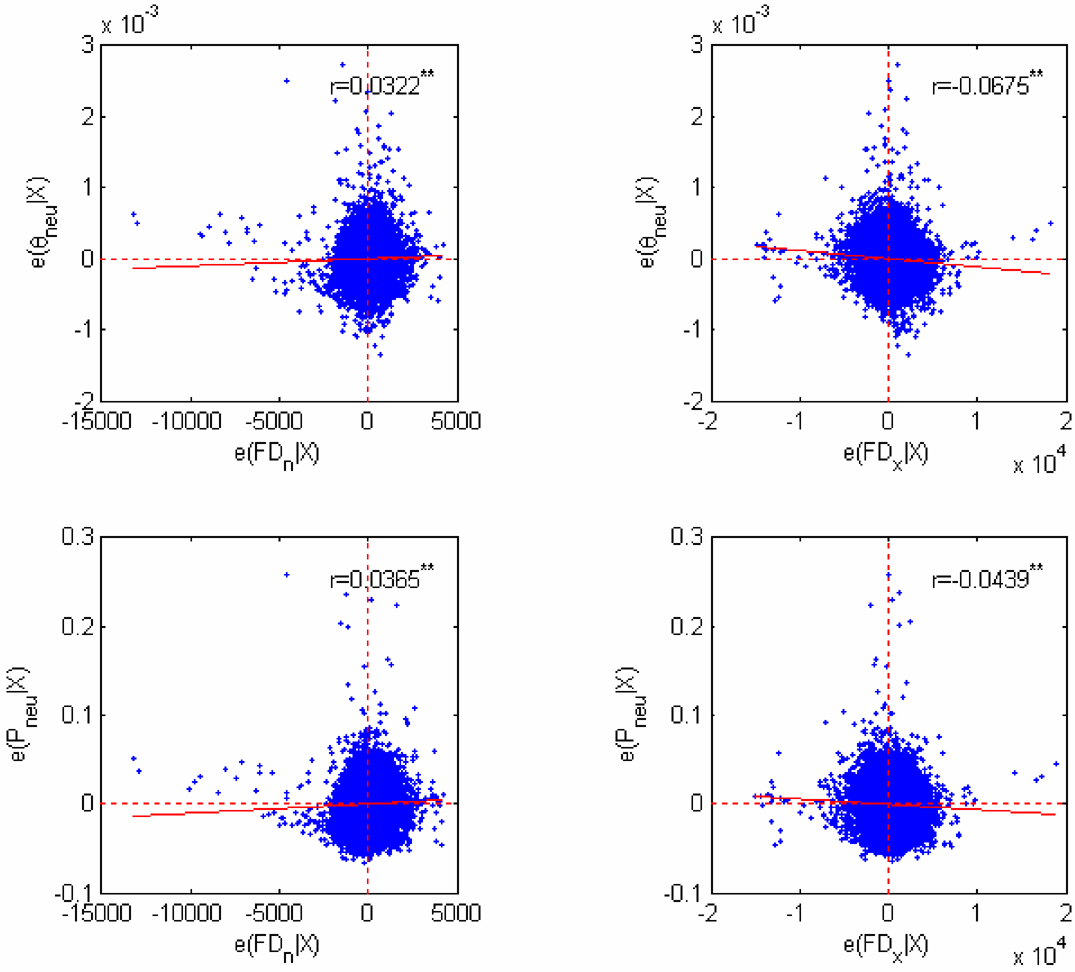

x 10^-3
r=0.0322**
e(θneu|X)
e(FDn|X)
x 10^-3
r=-0.0675**
e(θneu|X)
e(FDx|X)
x 10^4
r=0.0365**
e(Pneu|X)
e(FDn|X)
r=-0.0439**
e(Pneu|X)
e(FDx|X)
x 10^4
