# Supplementary material for: Pervasive Hitchhiking at Coding and Regulatory Sites in Humans
Source: PLoS Genet. 2009 Jan 16;5(1):e1000336. doi: 10.1371/journal.pgen.1000336 (PMC2613029; doi:10.1371/journal.pgen.1000336)
Supplement: Figure S5 — Relationships among the levels of functional divergence [i.e., the divergence at coding sites (Dn) or the divergence at conserved noncoding region (Dx)] and neutral polymorphism [i.e., the level of neutral polymorphism (θneu) or the level of normalized neutral polymorphism (Pneu = θneu/dneu)]. Scatter plots display values of two variables in gray dots for (A) Dn and θneu, (B) Dx and θneu, (C) Dn and Pneu, and (D) Dx and Pneu. Red circles are average values for the pooled gray dots in 100 bins each containing 1% of the data points. The solid, green line shows the fit of a linear model. The values of θneu and Pneu here are based on the Watson data. The results derived from the Perlegen data are given in Figure 3. (0.1 MB PDF) [file pgen.1000336.s005.pdf]

Figure S5. Relationships among the levels of functional divergence [i.e. the divergence at coding sites (D_n) or the divergence at conserved noncoding region (D_x)] and neutral polymorphism [i.e. the level of neutral polymorphism (θ_{neu}) or the level of normalized neutral polymorphism ($P_{neu}=\theta_{neu}/d_{neu}$)]. Scatter plots display values of two variables in gray dots for **(a)** D_n and θ_{neu}, **(b)** D_x and θ_{neu}, **(c)** D_n and P_{neu}, and **(d)** D_x and P_{neu}. Red circles are average values for the pooled gray dots in 100 bins each containing 1% of the data points. The solid, green line shows the fit of a linear model. The values of θ_{neu} and P_{neu} here are based on the Watson data. The results derived from the Perlegen data are given in Fig. 3.

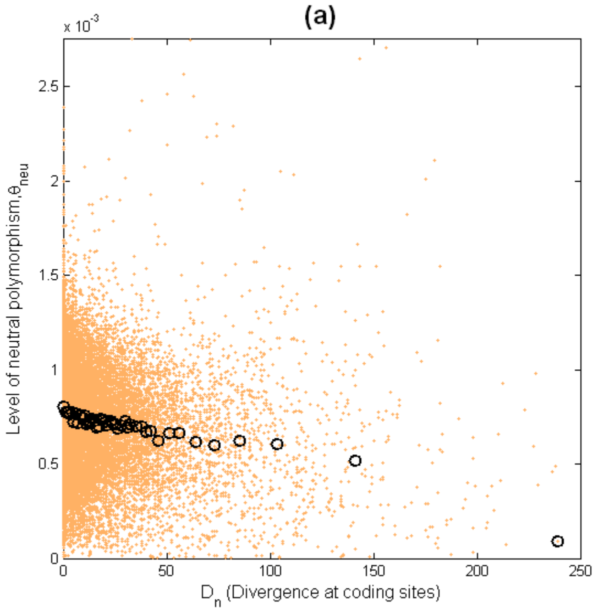

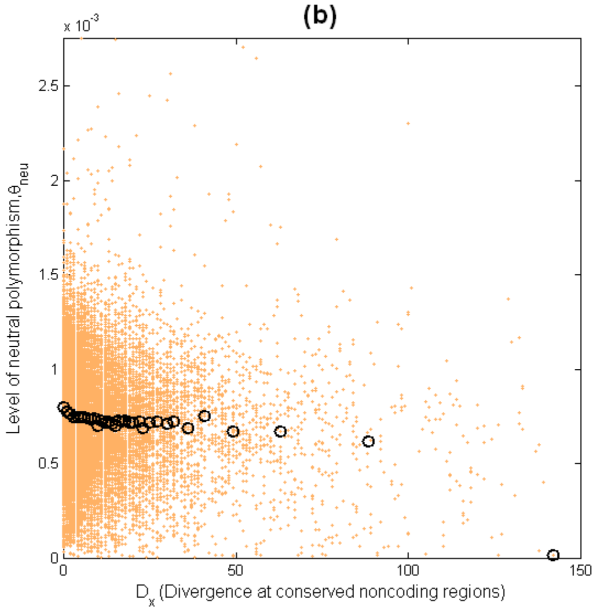

(c)

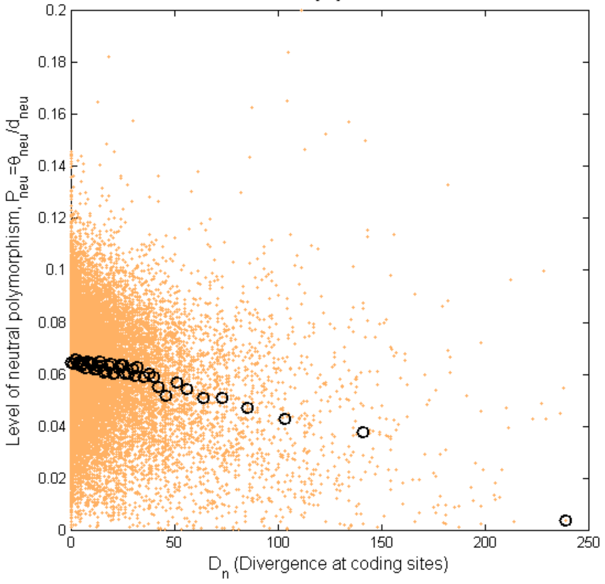

Level of neutral polymorphism, $P_{neu}=\theta_{neu}/d_{neu}$
D_n (Divergence at coding sites)
0
0.02
0.04
0.06
0.08
0.1
0.12
0.14
0.16
0.18
0.2
0
50
100
150
200
250

(d)

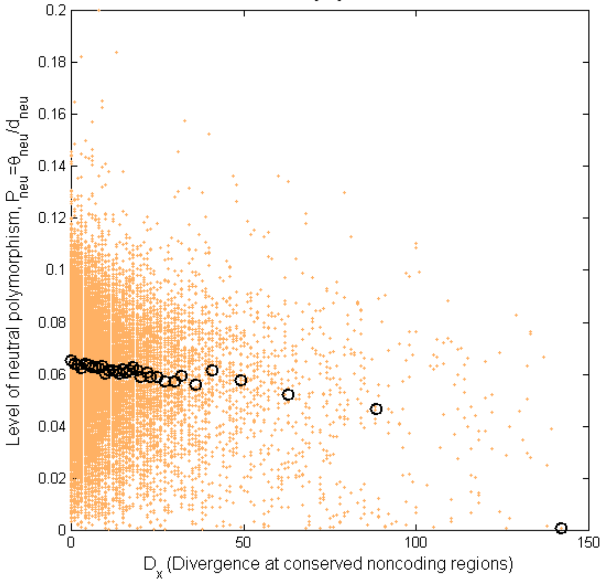

Level of neutral polymorphism, $P_{neu}=\theta_{neu}/d_{neu}$
D_x (Divergence at conserved noncoding regions)
0
0.02
0.04
0.06
0.08
0.1
0.12
0.14
0.16
0.18
0.2
0
50
100
150
