# Supplementary material for: Pervasive Hitchhiking at Coding and Regulatory Sites in Humans
Source: PLoS Genet. 2009 Jan 16;5(1):e1000336. doi: 10.1371/journal.pgen.1000336 (PMC2613029; doi:10.1371/journal.pgen.1000336)
Supplement: Figure S7 — Results derived from the sliding windows of 200 kb. Correlations between functional density [i.e., the number of codons (FDn) or the number of conserved noncoding sites (FDx)], and divergence [i.e., the divergence at coding sites (Dn) or the divergence at conserved noncoding region (Dx)] and neutral polymorphism [i.e., the level of neutral polymorphism (θneu) and the level of normalized neutral polymorphism (Pneu = θneu/dneu)] are given. The results are based on the Perlegen data. (0.1 MB PDF) [file pgen.1000336.s007.pdf]

Figure S7. Results derived from the sliding windows of 200 kb. Correlations between functional density [i.e. the number of codons (FD_n) or the number of conserved noncoding sites (FD_x)], and divergence [i.e. the divergence at coding sites (D_n) or the divergence at conserved noncoding region (D_x)] and neutral polymorphism [i.e. the level of neutral polymorphism (θ_{neu}) and the level of normalized neutral polymorphism ($P_{neu}=\theta_{neu}/d_{neu}$)] are given. The results are based on the Perlegen data.

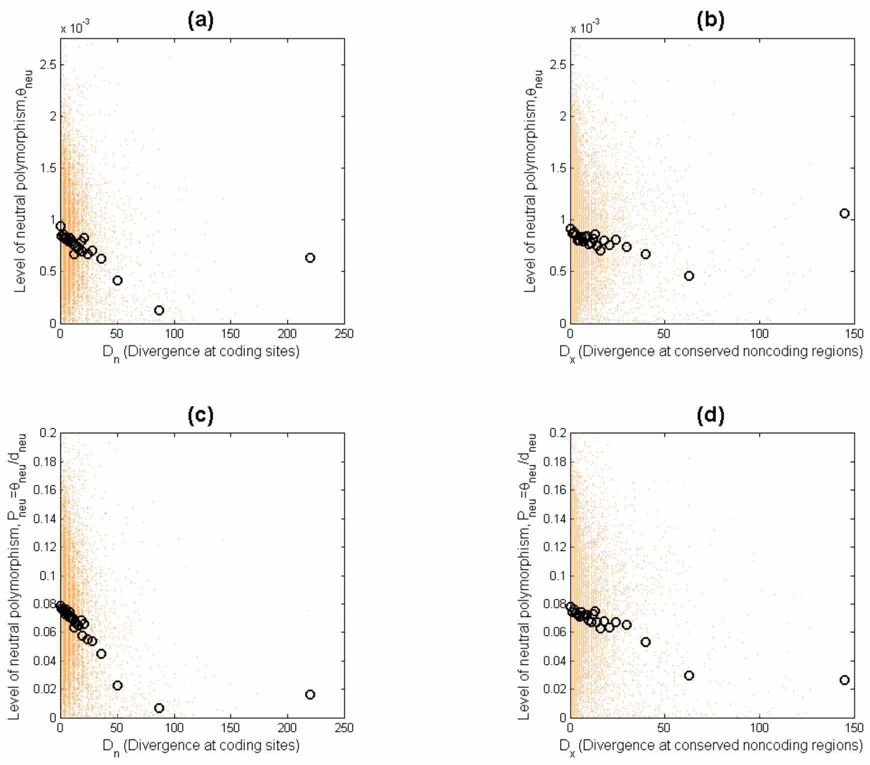

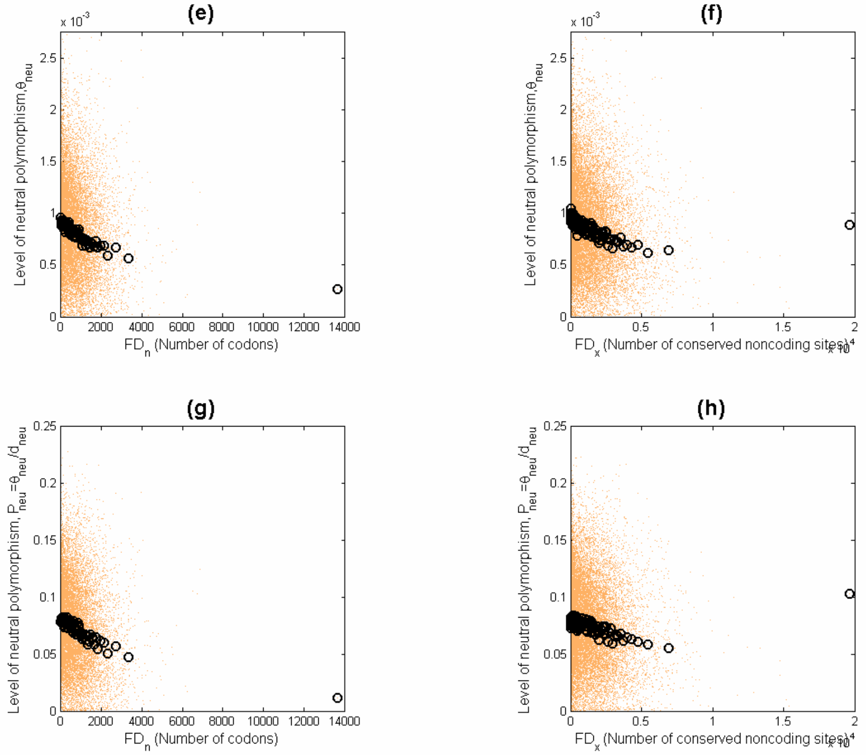
(e)
x 10-3
Level of neutral polymorphism, θneu
FDn (Number of codons)
(f)
x 10-3
Level of neutral polymorphism, θneu
FDx (Number of conserved noncoding sites)
x 10^4
(g)
Level of neutral polymorphism, Pneu=θneu/dneu
FDn (Number of codons)
(h)
Level of neutral polymorphism, Pneu=θneu/dneu
FDx (Number of conserved noncoding sites)
x 10^4
