# Supplementary material for: Pervasive Hitchhiking at Coding and Regulatory Sites in Humans
Source: PLoS Genet. 2009 Jan 16;5(1):e1000336. doi: 10.1371/journal.pgen.1000336 (PMC2613029; doi:10.1371/journal.pgen.1000336)
Supplement: Figure S8 — Results derived from the sliding windows of 200 kb. Correlations between functional density [i.e., the number of codons (FDn) or the number of conserved noncoding sites (FDx)], and divergence [i.e., the divergence at coding sites (Dn) or the divergence at conserved noncoding region (Dx)] and neutral polymorphism [i.e. the level of neutral polymorphism (θneu) or the level of normalized neutral polymorphism (Pneu = θneu/dneu)] are given. The results are based on the Watson data. (0.1 MB PDF) [file pgen.1000336.s008.pdf]

Figure S8. Results derived from the sliding windows of 200 kb. Correlations between functional density [i.e. the number of codons (FD_n) or the number of conserved noncoding sites (FD_x)], and divergence [i.e. the divergence at coding sites (D_n) or the divergence at conserved noncoding region (D_x)] and neutral polymorphism [i.e. the level of neutral polymorphism (θ_{neu}) or the level of normalized neutral polymorphism ($P_{neu}=\theta_{neu}/d_{neu}$)] are given. The results are based on the Watson data.

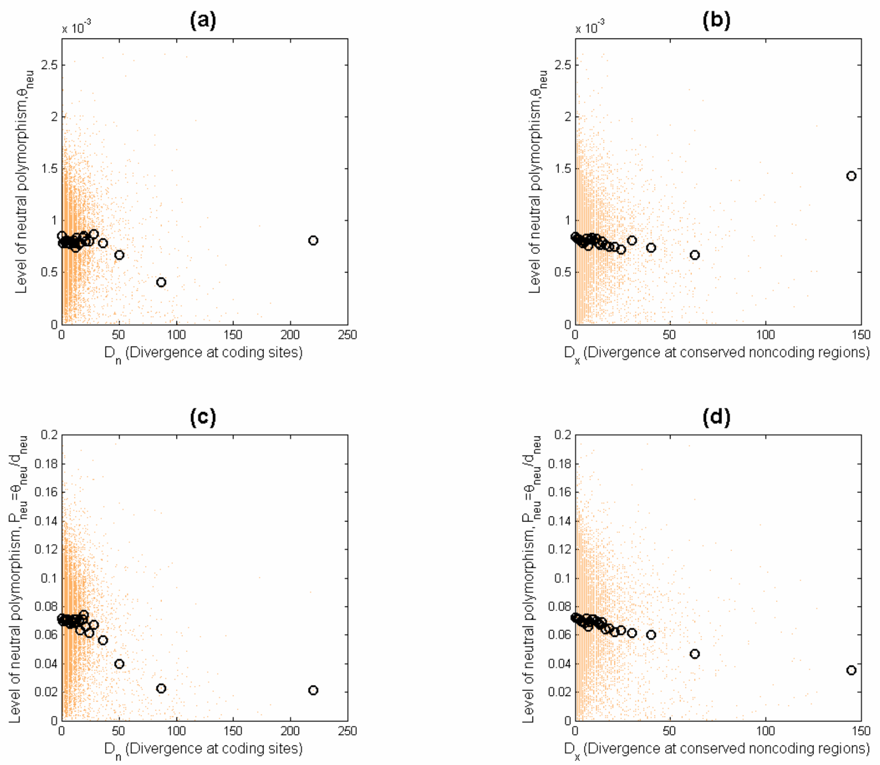

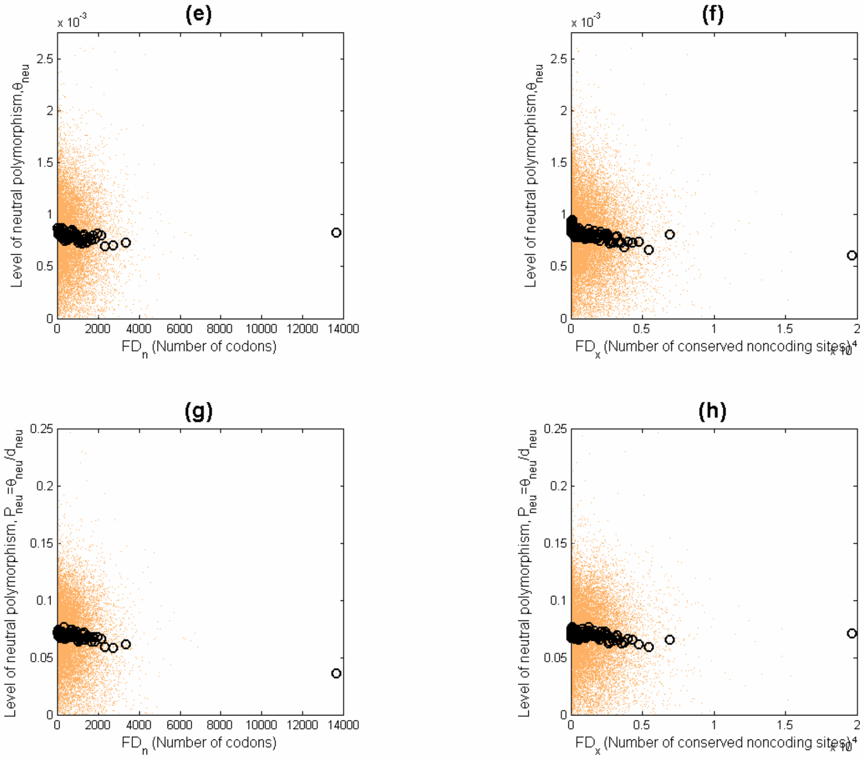
(e)
(f)
(g)
(h)
x 10^-3
Level of neutral polymorphism, θneu
Level of neutral polymorphism, Pneu=θneu/dneu
FDn (Number of codons)
FDx (Number of conserved noncoding sites)
x 10^4
0
2000
4000
6000
8000
10000
12000
14000
0.5
1
1.5
2
2.5
0.05
0.1
0.15
0.2
0.25
