# Supplementary material for: Pervasive Hitchhiking at Coding and Regulatory Sites in Humans
Source: PLoS Genet. 2009 Jan 16;5(1):e1000336. doi: 10.1371/journal.pgen.1000336 (PMC2613029; doi:10.1371/journal.pgen.1000336)
Supplement: Figure S10 — Results derived from the sliding windows of 600 kb. Correlations between functional density [i.e., the number of codons (FDn) or the number of conserved noncoding sites (FDx)], and divergence [i.e., the divergence at coding sites (Dn) or the divergence at conserved noncoding region (Dx)] and neutral polymorphism [i.e., the level of neutral polymorphism (θneu) and the level of normalized neutral polymorphism (Pneu = θneu/dneu)] are given. The results are based on the Watson data. (0.1 MB PDF) [file pgen.1000336.s010.pdf]

Figure S10. Results derived from the sliding windows of 600 kb. Correlations between functional density [i.e. the number of codons (FD_n) or the number of conserved noncoding sites (FD_x)], and divergence [i.e. the divergence at coding sites (D_n) or the divergence at conserved noncoding region (D_x)] and neutral polymorphism [i.e. the level of neutral polymorphism (θ_{neu}) and the level of normalized neutral polymorphism ($P_{neu}=\theta_{neu}/d_{neu}$)] are given. The results are based on the Watson data.

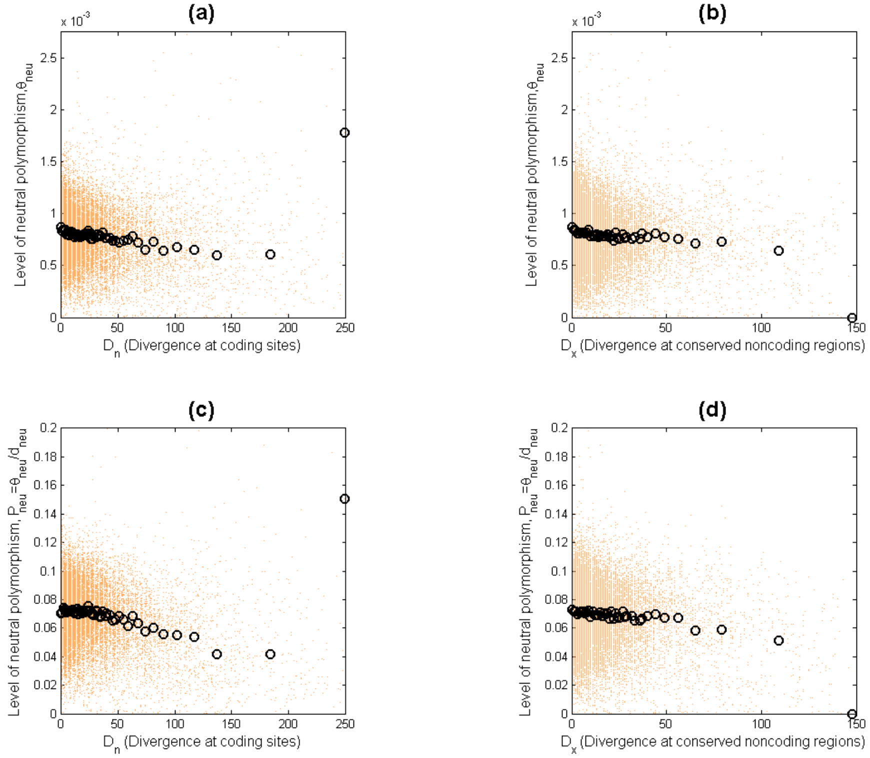

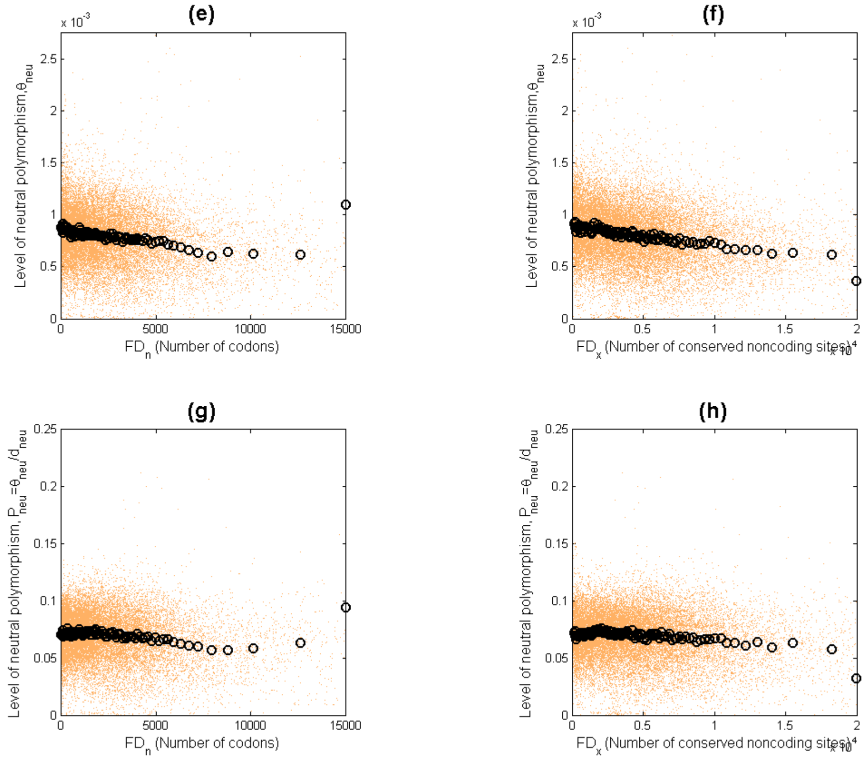
(e)
(f)
(g)
(h)
Level of neutral polymorphism,θ_{neu}
Level of neutral polymorphism, $P_{neu}=\theta_{neu}/d_{neu}$
FD_n (Number of codons)
FD_x (Number of conserved noncoding sites)
x 10^-3
x 10^4
