# Supplementary material for: Pervasive Hitchhiking at Coding and Regulatory Sites in Humans
Source: PLoS Genet. 2009 Jan 16;5(1):e1000336. doi: 10.1371/journal.pgen.1000336 (PMC2613029; doi:10.1371/journal.pgen.1000336)
Supplement: Table S1 — Correlation coefficients among the studied variables: the level of neutral polymorphism (θneu), he level of normalized neutral polymorphism (Pneu = θneu/dneu), recombination rate (RR), GC content (GC), the density of simple repeats (RD), the depth of sequencing coverage (SC), the divergence at coding sites (Dn), the divergence at conserved noncoding region (Dx), the number of codons (FDn), the number of conserved noncoding sites (FDx), and the level of neutral divergence (dneu). Spearman's ρ and Kendall's τ are given at the upper and lower diagonal parts of the table, respectively. The values of θneu and Pneu are based on the Watson data. (0.07 MB PDF) [file pgen.1000336.s011.pdf]

Table S1. Correlation coefficients among the studied variables: the level of neutral polymorphism (θ_{neu}), he level of normalized neutral polymorphism ($P_{neu}=\theta_{neu}/d_{neu}$), recombination rate (RR), GC content (GC), the density of simple repeats (RD), the depth of sequencing coverage (SC), the divergence at coding sites (D_n), the divergence at conserved noncoding region (D_x), the number of codons (FD_n), the number of conserved noncoding sites (FD_x), and the level of neutral divergence (d_{neu}). Spearman's ρ and Kendall's τ are given at the upper and lower diagonal parts of the table, respectively. The values of θ_{neu} and P_{neu} are based on the Watson data.

	θ_{neu}	P_{neu}	RR	GC	RD	SC	D_n	D_x	FD_n	FD_x	d_{neu}
θ_{neu}	—	0.9007**	0.3155**	-0.0875**	-0.0775**	0.1183**	-0.1855**	-0.1143**	-0.2247**	-0.2403**	0.3663**
P_{neu}	0.7361**	—	0.2098**	-0.0514**	-0.1086**	0.1364**	-0.1363**	-0.1075**	-0.1290**	-0.1384**	-0.0122 (0.0631)
RR	0.2149**	0.1412**	—	0.3432**	-0.2738**	0.0819**	0.0373**	-0.0519**	0.0189*	-0.0315**	0.3024**
GC	-0.0586**	-0.0342**	0.2351**	—	-0.0554**	-0.4729**	0.5789**	0.1934**	0.6192**	0.5183**	-0.1069**
RD	-0.0520**	-0.0730**	-0.1856**	-0.0370**	—	-0.4839**	0.0300**	0.0622**	-0.0147 (0.0256)	-0.0265**	0.0415**
SC	0.0787**	0.0912**	0.0564**	-0.3175**	-0.3415**	—	-0.4579**	-0.2683**	-0.4321**	-0.3821**	-0.0159 (0.0155)
D_n	-0.1304**	-0.0950**	0.0263**	0.4218**	0.0217**	-0.3279**	—	0.3078**	0.8964**	0.6824**	-0.1550**
D_x	-0.0790**	-0.0742**	-0.0358**	0.1319**	0.0429**	-0.1867**	0.2239**	—	0.3028**	0.4962**	-0.0260**
FD_n	-0.1540**	-0.0872**	0.0136*	0.4479**	-0.0095 (0.0329)	-0.2968**	0.7411**	0.2131**	—	0.8283**	-0.2748**
FD_x	-0.1617**	-0.0921**	-0.0205**	0.3610**	-0.0179**	-0.2587**	0.5099**	0.3519**	0.6533**	—	-0.2926**
d_{neu}	0.2553**	-0.0087 (0.0478)	0.2075**	-0.0734**	0.0280**	-0.0099 (0.0242)	-0.1124**	-0.0188**	-0.1903**	-0.1990**	—

** Correlation is significant at the 0.001 level (2-tailed).
* Correlation is significant at the 0.01 level (2-tailed).
