# Supplementary material for: Pervasive Hitchhiking at Coding and Regulatory Sites in Humans
Source: PLoS Genet. 2009 Jan 16;5(1):e1000336. doi: 10.1371/journal.pgen.1000336 (PMC2613029; doi:10.1371/journal.pgen.1000336)
Supplement: Table S2 — Correlation coefficients and partials correlation coefficients between recombination rate (RR) and levels of neutral polymorphism [i.e., the level of neutral polymorphism (θneu) and the level of normalized neutral polymorphism (Pneu = θneu/dneu)]. Spearman's partial correlation coefficients were calculated by controlling for all possible combinations of potentially confounding variables. The results of representative combinations are given here. Closed circles (•) indicate the controlled variables. These variables are GC content (GC), the density of simple repeats (RD), the divergence at coding sites (Dn), the divergence at conserved noncoding region (Dx), the number of codons (FDn), the number of conserved noncoding sites (FDx), and the level of neutral divergence rate (dneu). Open circles (○) indicate the variables that were not controlled in a particular analysis. (P) indicates the results are based on the Perlegen data, (W) indicates the results are based on the Watson data. P-values are given in parentheses. (0.1 MB PDF) [file pgen.1000336.s012.pdf]

Table S2. Correlation coefficients and partials correlation coefficients between recombination rate (RR) and levels of neutral polymorphism [i.e. the level of neutral polymorphism (θ_{neu}) and the level of normalized neutral polymorphism ($P_{neu}=\theta_{neu}/d_{neu}$)]. Spearman's partial correlation coefficients were calculated by controlling for all possible combinations of potentially confounding variables. The results of representative combinations are given here. Closed circles (●) indicate the controlled variables. These variables are GC content (GC), the density of simple repeats (RD), the divergence at coding sites (D_n), the divergence at conserved noncoding region (D_x), the number of codons (FD_n), the number of conserved noncoding sites (FD_x), and the level of neutral divergence rate (d_{neu}). Open circles (○) indicate the variables that were not controlled in a particular analysis. (P) indicates the results are based on the Perlegen data, (W) indicates the results are based on the Watson data. *P*-values are given in parentheses.

**RR and θ_{neu} (and P_{neu})**										
RR and θ_{neu} (P)	RR and P_{neu} (P)	RR and θ_{neu} (W)	RR and P_{neu} (W)	GC	RD	D_n	D_x	FD_n	FD_x	d_{neu}
0.2187*** (0)	0.1309*** (0)	0.3155*** (0)	0.2098*** (0)	○	○	○	○	○	○	○
0.3511*** (0)	0.2403*** (0)	0.3693*** (0)	0.2424*** (0)	●	○	○	○	○	○	○
0.1986*** (0)	0.0995*** (0)	0.3068*** (0)	0.1883*** (0)	○	●	○	○	○	○	○
0.2439*** (0)	0.1475*** (0)	0.3283*** (0)	0.2170*** (0)	○	○	●	○	○	○	○
0.2144*** (0)	0.1255*** (0)	0.3120*** (0)	0.2056*** (0)	○	○	○	●	○	○	○
0.2401*** (0)	0.1413*** (0)	0.3281*** (0)	0.2140*** (0)	○	○	○	○	●	○	○
0.2222*** (0)	0.1288*** (0)	0.3173*** (0)	0.2075*** (0)	○	○	○	○	○	●	○
0.1469*** (0)	0.1421*** (0)	0.2308*** (0)	0.2240*** (0)	○	○	○	○	○	○	●
0.3317*** (0)	0.2094*** (0)	0.3616*** (0)	0.2217*** (0)	●	●	○	○	○	○	○
0.3279*** (0)	0.2200*** (0)	0.3468*** (0)	0.2217*** (0)	●	○	●	○	○	○	○
0.3414*** (0)	0.2295*** (0)	0.3612*** (0)	0.2327*** (0)	●	○	○	●	○	○	○
0.3165*** (0)	0.2203*** (0)	0.3314*** (0)	0.2192*** (0)	●	○	○	○	●	○	○
0.3159*** (0)	0.2212*** (0)	0.3294*** (0)	0.2176*** (0)	●	○	○	○	○	●	○
0.2838*** (0)	0.2788*** (0)	0.2743*** (0)	0.2672*** (0)	●	○	○	○	○	○	●
0.2254*** (0)	0.1171*** (0)	0.3214*** (0)	0.1966*** (0)	○	●	●	○	○	○	○
0.1965*** (0)	0.0962*** (0)	0.3049*** (0)	0.1857*** (0)	○	●	○	●	○	○	○
0.2172*** (0)	0.1077*** (0)	0.3187*** (0)	0.1920*** (0)	○	●	○	○	●	○	○
0.1975*** (0)	0.0940*** (0)	0.3065*** (0)	0.1846*** (0)	○	●	○	○	○	●	○
0.1149*** (0)	0.1083*** (0)	0.2116*** (0)	0.2019*** (0)	○	●	○	○	○	○	●
0.2399*** (0)	0.1428*** (0)	0.3255*** (0)	0.2134*** (0)	○	○	●	●	○	○	○
0.2413*** (0)	0.1464*** (0)	0.3270*** (0)	0.2166*** (0)	○	○	●	○	●	○	○
0.2354*** (0)	0.1429*** (0)	0.3210*** (0)	0.2132*** (0)	○	○	●	○	○	●	○
0.1812*** (0)	0.1753*** (0)	0.2467*** (0)	0.2398*** (0)	○	○	●	○	○	○	●
0.2369*** (0)	0.1370*** (0)	0.3261*** (0)	0.2106*** (0)	○	○	○	●	●	○	○

0.2218*** (0)	0.1269*** (0)	0.3179*** (0)	0.2060*** (0)	○	○	○	●	○	●	○
0.1435*** (0)	0.1385*** (0)	0.2273*** (0)	0.2205*** (0)	○	○	○	●	○	○	●
0.2349*** (0)	0.1387*** (0)	0.3225*** (0)	0.2103*** (0)	○	○	○	○	●	●	○
0.1872*** (0)	0.1814*** (0)	0.2502*** (0)	0.2431*** (0)	○	○	○	○	●	○	●
0.1711*** (0)	0.1655*** (0)	0.2433*** (0)	0.2363*** (0)	○	○	○	○	○	●	●
0.3102*** (0)	0.1908*** (0)	0.3408*** (0)	0.2028*** (0)	●	●	●	○	○	○	○
0.3237*** (0)	0.2005*** (0)	0.3548*** (0)	0.2136*** (0)	●	●	○	●	○	○	○
0.2954*** (0)	0.1881*** (0)	0.3230*** (0)	0.1979*** (0)	●	●	○	○	●	○	○
0.2934*** (0)	0.1878*** (0)	0.3197*** (0)	0.1951*** (0)	●	●	○	○	○	●	○
0.2540*** (0)	0.2474*** (0)	0.2565*** (0)	0.2465*** (0)	●	●	○	○	○	○	●
0.3236*** (0)	0.2148*** (0)	0.3436*** (0)	0.2172*** (0)	●	○	●	●	○	○	○
0.3169*** (0)	0.2230*** (0)	0.3310*** (0)	0.2205*** (0)	●	○	●	○	●	○	○
0.3121*** (0)	0.2160*** (0)	0.3268*** (0)	0.2129*** (0)	●	○	●	○	○	●	○
0.2654*** (0)	0.2614*** (0)	0.2563*** (0)	0.2491*** (0)	●	○	●	○	○	○	●
0.3131*** (0)	0.2157*** (0)	0.3290*** (0)	0.2152*** (0)	●	○	○	●	●	○	○
0.3160*** (0)	0.2215*** (0)	0.3295*** (0)	0.2176*** (0)	●	○	○	●	○	●	○
0.2731*** (0)	0.2684*** (0)	0.2640*** (0)	0.2573*** (0)	●	○	○	●	○	○	●
0.3111*** (0)	0.2179*** (0)	0.3247*** (0)	0.2150*** (0)	●	○	○	○	●	●	○
0.2668*** (0)	0.2624*** (0)	0.2561*** (0)	0.2490*** (0)	●	○	○	○	●	○	●
0.2677*** (0)	0.2634*** (0)	0.2555*** (0)	0.2484*** (0)	●	○	○	○	○	●	●
0.2225*** (0)	0.1137*** (0)	0.3193*** (0)	0.1939*** (0)	○	●	●	●	○	○	○
0.2183*** (0)	0.1142*** (0)	0.3165*** (0)	0.1955*** (0)	○	●	●	○	●	○	○
0.2127*** (0)	0.1101*** (0)	0.3108*** (0)	0.1911*** (0)	○	●	●	○	○	●	○
0.1521*** (0)	0.1443*** (0)	0.2295*** (0)	0.2196*** (0)	○	●	●	○	○	○	●
0.2153*** (0)	0.1050*** (0)	0.3174*** (0)	0.1898*** (0)	○	●	○	●	●	○	○
0.1975*** (0)	0.0935*** (0)	0.3068*** (0)	0.1841*** (0)	○	●	○	●	○	●	○
0.1139*** (0)	0.1073*** (0)	0.2098*** (0)	0.2001*** (0)	○	●	○	●	○	○	●
0.2108*** (0)	0.1043*** (0)	0.3120*** (0)	0.1875*** (0)	○	●	○	○	●	●	○
0.1553*** (0)	0.1479*** (0)	0.2315*** (0)	0.2216*** (0)	○	●	○	○	●	○	●
0.1379*** (0)	0.1306*** (0)	0.2238*** (0)	0.2139*** (0)	○	●	○	○	○	●	●
0.2379*** (0)	0.1420*** (0)	0.3247*** (0)	0.2131*** (0)	○	○	●	●	●	○	○
0.2348*** (0)	0.1411*** (0)	0.3214*** (0)	0.2118*** (0)	○	○	●	●	○	●	○
0.1762*** (0)	0.1704*** (0)	0.2423*** (0)	0.2357*** (0)	○	○	●	●	○	○	●
0.2365*** (0)	0.1427*** (0)	0.3223*** (0)	0.2125*** (0)	○	○	●	○	●	●	○
0.1863*** (0)	0.1806*** (0)	0.2495*** (0)	0.2424*** (0)	○	○	●	○	●	○	●

0.1831*** (0)	0.1770*** (0)	0.2476*** (0)	0.2407*** (0)	○	○	●	○	○	●	●
0.2343*** (0)	0.1375*** (0)	0.3225*** (0)	0.2094*** (0)	○	○	○	●	●	●	○
0.1816*** (0)	0.1760*** (0)	0.2454*** (0)	0.2386*** (0)	○	○	○	●	●	○	●
0.1680*** (0)	0.1625*** (0)	0.2407*** (0)	0.2340*** (0)	○	○	○	●	○	●	●
0.1853*** (0)	0.1797*** (0)	0.2479*** (0)	0.2408*** (0)	○	○	○	○	●	●	●
0.3068*** (0)	0.1867*** (0)	0.3382*** (0)	0.1993*** (0)	●	●	●	●	○	○	○
0.2957*** (0)	0.1922*** (0)	0.3216*** (0)	0.2002*** (0)	●	●	●	○	●	○	○
0.2910*** (0)	0.1846*** (0)	0.3180*** (0)	0.1920*** (0)	●	●	●	○	○	●	○
0.2378*** (0)	0.2320*** (0)	0.2406*** (0)	0.2305*** (0)	●	●	●	○	○	○	●
0.2932*** (0)	0.1850*** (0)	0.3214*** (0)	0.1951*** (0)	●	●	○	●	●	○	○
0.2939*** (0)	0.1896*** (0)	0.3195*** (0)	0.1959*** (0)	●	●	○	●	○	●	○
0.2453*** (0)	0.2389*** (0)	0.2478*** (0)	0.2382*** (0)	●	●	○	●	○	○	●
0.2888*** (0)	0.1848*** (0)	0.3153*** (0)	0.1928*** (0)	●	●	○	○	●	●	○
0.2368*** (0)	0.2308*** (0)	0.2384*** (0)	0.2284*** (0)	●	●	○	○	●	○	●
0.2367*** (0)	0.2307*** (0)	0.2368*** (0)	0.2267*** (0)	●	●	○	○	○	●	●
0.3134*** (0)	0.2185*** (0)	0.3284*** (0)	0.2166*** (0)	●	○	●	●	●	○	○
0.3121*** (0)	0.2162*** (0)	0.3269*** (0)	0.2128*** (0)	●	○	●	●	○	●	○
0.2598*** (0)	0.2559*** (0)	0.2511*** (0)	0.2442*** (0)	●	○	●	●	○	○	●
0.3113*** (0)	0.2191*** (0)	0.3247*** (0)	0.2151*** (0)	●	○	●	○	●	●	○
0.2647*** (0)	0.2605*** (0)	0.2546*** (0)	0.2475*** (0)	●	○	●	○	●	○	●
0.2612*** (0)	0.2573*** (0)	0.2503*** (0)	0.2431*** (0)	●	○	●	○	○	●	●
0.3104*** (0)	0.2167*** (0)	0.3247*** (0)	0.2139*** (0)	●	○	○	●	●	●	○
0.2611*** (0)	0.2569*** (0)	0.2508*** (0)	0.2441*** (0)	●	○	○	●	●	○	●
0.2654*** (0)	0.2611*** (0)	0.2538*** (0)	0.2470*** (0)	●	○	○	●	○	●	●
0.2646*** (0)	0.2604*** (0)	0.2530*** (0)	0.2459*** (0)	●	○	○	○	●	●	●
0.2161*** (0)	0.1112*** (0)	0.3148*** (0)	0.1930*** (0)	○	●	●	●	●	○	○
0.2127*** (0)	0.1099*** (0)	0.3110*** (0)	0.1907*** (0)	○	●	●	●	○	●	○
0.1483*** (0)	0.1406*** (0)	0.2260*** (0)	0.2164*** (0)	○	●	●	●	○	○	●
0.2130*** (0)	0.1101*** (0)	0.3114*** (0)	0.1909*** (0)	○	●	●	○	●	●	○
0.1560*** (0)	0.1485*** (0)	0.2317*** (0)	0.2217*** (0)	○	●	●	○	●	○	●
0.1522*** (0)	0.1444*** (0)	0.2291*** (0)	0.2192*** (0)	○	●	●	○	○	●	●
0.2111*** (0)	0.1050*** (0)	0.3120*** (0)	0.1877*** (0)	○	●	○	●	●	●	○
0.1514*** (0)	0.1441*** (0)	0.2280*** (0)	0.2182*** (0)	○	●	○	●	●	○	●
0.1361*** (0)	0.1289*** (0)	0.2221*** (0)	0.2125*** (0)	○	●	○	●	○	●	●
0.1528*** (0)	0.1455*** (0)	0.2286*** (0)	0.2186*** (0)	○	●	○	○	●	●	●

0.2357*** (0)	0.1412*** (0)	0.3222*** (0)	0.2115*** (0)	○	○	●	●	●	●	○
0.1811*** (0)	0.1755*** (0)	0.2450*** (0)	0.2382*** (0)	○	○	●	●	●	○	●
0.1795*** (0)	0.1735*** (0)	0.2448*** (0)	0.2382*** (0)	○	○	●	●	○	●	●
0.1836*** (0)	0.1781*** (0)	0.2465*** (0)	0.2393*** (0)	○	○	●	○	●	●	●
0.1815*** (0)	0.1760*** (0)	0.2452*** (0)	0.2384*** (0)	○	○	○	●	●	●	●
0.2932*** (0)	0.1890*** (0)	0.3198*** (0)	0.1973*** (0)	●	●	●	●	●	○	○
0.2915*** (0)	0.1863*** (0)	0.3178*** (0)	0.1928*** (0)	●	●	●	●	○	●	○
0.2334*** (0)	0.2277*** (0)	0.2362*** (0)	0.2265*** (0)	●	●	●	●	○	○	●
0.2896*** (0)	0.1879*** (0)	0.3150*** (0)	0.1944*** (0)	●	●	●	○	●	●	○
0.2363*** (0)	0.2303*** (0)	0.2379*** (0)	0.2279*** (0)	●	●	●	○	●	○	●
0.2322*** (0)	0.2265*** (0)	0.2331*** (0)	0.2230*** (0)	●	●	●	○	○	●	●
0.2891*** (0)	0.1855*** (0)	0.3153*** (0)	0.1928*** (0)	●	●	○	●	●	●	○
0.2327*** (0)	0.2267*** (0)	0.2343*** (0)	0.2246*** (0)	●	●	○	●	●	○	●
0.2357*** (0)	0.2298*** (0)	0.2359*** (0)	0.2260*** (0)	●	●	○	●	○	●	●
0.2340*** (0)	0.2281*** (0)	0.2346*** (0)	0.2246*** (0)	●	●	○	○	●	●	●
0.3107*** (0)	0.2179*** (0)	0.3247*** (0)	0.2142*** (0)	●	○	●	●	●	●	○
0.2594*** (0)	0.2554*** (0)	0.2497*** (0)	0.2430*** (0)	●	○	●	●	●	○	●
0.2586*** (0)	0.2547*** (0)	0.2484*** (0)	0.2415*** (0)	●	○	●	●	○	●	●
0.2613*** (0)	0.2573*** (0)	0.2503*** (0)	0.2431*** (0)	●	○	●	○	●	●	●
0.2609*** (0)	0.2568*** (0)	0.2503*** (0)	0.2435*** (0)	●	○	○	●	●	●	●
0.2129*** (0)	0.1099*** (0)	0.3114*** (0)	0.1907*** (0)	○	●	●	●	●	●	○
0.1522*** (0)	0.1448*** (0)	0.2282*** (0)	0.2185*** (0)	○	●	●	●	●	○	●
0.1501*** (0)	0.1424*** (0)	0.2273*** (0)	0.2177*** (0)	○	●	●	●	○	●	●
0.1531*** (0)	0.1457*** (0)	0.2284*** (0)	0.2184*** (0)	○	●	●	○	●	●	●
0.1509*** (0)	0.1436*** (0)	0.2271*** (0)	0.2173*** (0)	○	●	○	●	●	●	●
0.1808*** (0)	0.1753*** (0)	0.2446*** (0)	0.2377*** (0)	○	○	●	●	●	●	●
0.2896*** (0)	0.1879*** (0)	0.3150*** (0)	0.1942*** (0)	●	●	●	●	●	●	○
0.2323*** (0)	0.2264*** (0)	0.2340*** (0)	0.2243*** (0)	●	●	●	●	●	○	●
0.2310*** (0)	0.2253*** (0)	0.2321*** (0)	0.2222*** (0)	●	●	●	●	○	●	●
0.2325*** (0)	0.2268*** (0)	0.2332*** (0)	0.2232*** (0)	●	●	●	○	●	●	●
0.2321*** (0)	0.2262*** (0)	0.2331*** (0)	0.2233*** (0)	●	●	○	●	●	●	●
0.2588*** (0)	0.2548*** (0)	0.2485*** (0)	0.2417*** (0)	●	○	●	●	●	●	●
0.1515*** (0)	0.1441*** (0)	0.2272*** (0)	0.2175*** (0)	○	●	●	●	●	●	●
0.2312*** (0)	0.2254*** (0)	0.2322*** (0)	0.2223*** (0)	●	●	●	●	●	●	●
0.2579*** (0)	0.2073*** (0)	0.2445*** (0)	0.2020*** (0)	●	●	●	●	●	●	●

*** $P<0.0001$; ** $0.0001 \leq P<0.001$; * $0.001 \leq P<0.01$
