# Supplementary material for: Pervasive Hitchhiking at Coding and Regulatory Sites in Humans
Source: PLoS Genet. 2009 Jan 16;5(1):e1000336. doi: 10.1371/journal.pgen.1000336 (PMC2613029; doi:10.1371/journal.pgen.1000336)
Supplement: Table S3 — Spearman rank correlation coefficients and partials correlation coefficients between functional divergence [i.e., the divergence at coding sites (Dn) or the divergence at conserved noncoding region (Dx)] and neutral polymorphism [i.e., the level of neutral polymorphism (θneu) and the level of normalized neutral polymorphism (Pneu = θneu/dneu)], and between functional constraints [i.e., the number of codons (FDn) and the number of conserved noncoding sites (FDx)] and neutral polymorphism (θneu and Pneu). Spearman's partial correlation coefficients were calculated by controlling for all possible combinations of potentially confounding variables. The results of representative combinations are given here. Closed circles (•) indicate the controlled variables. These variables are GC content (GC), the density of simple repeats (RD), the divergence at coding sites (Dn), the divergence at conserved noncoding region (Dx), the number of codons (FDn), the number of conserved noncoding sites (FDx), and the level of neutral divergence rate (dneu). Open circles (○) indicate the variables that were not controlled in a particular analysis. (P) indicates the results are based on the Perlegen data, (W) indicates the results are based on the Watson data. P-values are given in parentheses. (0.2 MB PDF) [file pgen.1000336.s013.pdf]

Table S3. Spearman rank correlation coefficients and partials correlation coefficients between functional divergence [i.e. the divergence at coding sites (D_n) or the divergence at conserved noncoding region (D_x)] and neutral polymorphism [i.e. the level of neutral polymorphism (θ_{neu}) and the level of normalized neutral polymorphism ($P_{neu}=\theta_{neu}/d_{neu}$)], and between functional constraints [i.e. the number of codons (FD_n) and the number of conserved noncoding sites (FD_x)] and neutral polymorphism (θ_{neu} and P_{neu}). Spearman's partial correlation coefficients were calculated by controlling for all possible combinations of potentially confounding variables. The results of representative combinations are given here. Closed circles (●) indicate the controlled variables. These variables are GC content (GC), the density of simple repeats (RD), the divergence at coding sites (D_n), the divergence at conserved noncoding region (D_x), the number of codons (FD_n), the number of conserved noncoding sites (FD_x), and the level of neutral divergence rate (d_{neu}). Open circles (○) indicate the variables that were not controlled in a particular analysis. (P) indicates the results are based on the Perlegen data, (W) indicates the results are based on the Watson data. *P*-values are given in parentheses.

**D_n and θ_{neu} (or P_{neu})**											
θ_{neu} (P)	P_{neu} (P)	θ_{neu} (W)	P_{neu} (W)	RR	GC	RD	D_n	D_x	FD_n	FD_x	d_{neu}
-0.2939*** (0)	-0.2467*** (0)	-0.1855*** (0)	-0.1363*** (0)	○	○	○	○	○	○	○	○
-0.3123*** (0)	-0.2555*** (0)	-0.2080*** (0)	-0.1475*** (0)	●	○	○	○	○	○	○	○
-0.1740*** (0)	-0.1338*** (0)	-0.1660*** (0)	-0.1308*** (0)	○	●	○	○	○	○	○	○
-0.2932*** (0)	-0.2459*** (0)	-0.1838*** (0)	-0.1339*** (0)	○	○	●	○	○	○	○	○
-0.2594*** (0)	-0.2121*** (0)	-0.1590*** (0)	-0.1091*** (0)	○	○	○	○	●	○	○	○
-0.0162 (1.511406e-002)	-0.0814*** (0)	0.0369*** (2.126458e-008)	-0.0471*** (8.087975e-013)	○	○	○	○	○	●	○	○
-0.1206*** (0)	-0.1396*** (0)	-0.0303*** (4.159202e-006)	-0.0578*** (0)	○	○	○	○	○	○	●	○
-0.2590*** (0)	-0.2534*** (0)	-0.1400*** (0)	-0.1399*** (0)	○	○	○	○	○	○	○	●
-0.1133*** (0)	-0.0904*** (0)	-0.0970*** (0)	-0.0841*** (0)	●	●	○	○	○	○	○	○
-0.3111*** (0)	-0.2532*** (0)	-0.2086*** (0)	-0.1456*** (0)	●	○	●	○	○	○	○	○
-0.2804*** (0)	-0.2225*** (0)	-0.1861*** (0)	-0.1235*** (0)	●	○	○	○	●	○	○	○
-0.0300*** (6.391491e-006)	-0.0900*** (0)	0.0231** (4.480959e-004)	-0.0583*** (0)	●	○	○	○	○	●	○	○
-0.1442*** (0)	-0.1528*** (0)	-0.0590*** (0)	-0.0764*** (0)	●	○	○	○	○	○	●	○
-0.2789*** (0)	-0.2725*** (0)	-0.1657*** (0)	-0.1647*** (0)	●	○	○	○	○	○	○	●
-0.1668*** (0)	-0.1250*** (0)	-0.1607*** (0)	-0.1234*** (0)	○	●	●	○	○	○	○	○
-0.1505*** (0)	-0.1103*** (0)	-0.1467*** (0)	-0.1103*** (0)	○	●	○	○	●	○	○	○
-0.0092 (1.689161e-001)	-0.0733*** (0)	0.0324*** (8.622048e-007)	-0.0498*** (3.919087e-014)	○	●	○	○	○	●	○	○
-0.0755*** (0)	-0.0893*** (0)	-0.0497*** (4.007905e-014)	-0.0712*** (0)	○	●	○	○	○	○	●	○
-0.1488*** (0)	-0.1411*** (0)	-0.1344*** (0)	-0.1338*** (0)	○	●	○	○	○	○	○	●
-0.2602*** (0)	-0.2132*** (0)	-0.1586*** (0)	-0.1085*** (0)	○	○	●	○	●	○	○	○
-0.0043 (5.150299e-001)	-0.0683*** (0)	0.0453*** (5.685674e-012)	-0.0366*** (2.557476e-008)	○	○	●	○	○	●	○	○
-0.1139*** (0)	-0.1322*** (0)	-0.0247** (1.701308e-004)	-0.0507*** (1.210143e-014)	○	○	●	○	○	○	●	○
-0.2570*** (0)	-0.2514*** (0)	-0.1371*** (0)	-0.1368*** (0)	○	○	●	○	○	○	○	●
-0.0102 (1.250525e-001)	-0.0747*** (0)	0.0413*** (3.287024e-010)	-0.0411*** (4.100734e-010)	○	○	○	○	●	●	○	○

-0.1215*** (0)	-0.1430*** (0)	-0.0300*** (4.988506e-006)	-0.0601*** (0)	○	○	○	○	●	○	●	○
-0.2242*** (0)	-0.2193*** (0)	-0.1114*** (0)	-0.1128*** (0)	○	○	○	○	●	○	○	●
-0.0375*** (1.696521e-008)	-0.0935*** (0)	0.0133 (4.343108e-002)	-0.0628*** (0)	○	○	○	○	○	●	●	○
-0.0674*** (0)	-0.0616*** (0)	-0.0354*** (7.152455e-008)	-0.0373*** (1.431946e-008)	○	○	○	○	○	●	○	●
-0.1378*** (0)	-0.1342*** (0)	-0.0535*** (4.440892e-016)	-0.0544*** (1.110223e-016)	○	○	○	○	○	○	●	●
-0.1128*** (0)	-0.0892*** (0)	-0.0974*** (0)	-0.0832*** (0)	●	●	●	○	○	○	○	○
-0.0976*** (0)	-0.0729*** (0)	-0.0866*** (0)	-0.0701*** (0)	●	●	○	○	●	○	○	○
-0.0190* (4.374306e-003)	-0.0814*** (0)	0.0278*** (2.466870e-005)	-0.0552*** (0)	●	●	○	○	○	●	○	○
-0.0550*** (1.110223e-016)	-0.0749*** (0)	-0.0234** (3.689300e-004)	-0.0544*** (1.110223e-016)	●	●	○	○	○	○	●	○
-0.1069*** (0)	-0.0995*** (0)	-0.0892*** (0)	-0.0897*** (0)	●	●	○	○	○	○	○	●
-0.2798*** (0)	-0.2214*** (0)	-0.1866*** (0)	-0.1223*** (0)	●	○	●	○	●	○	○	○
-0.0235** (4.143937e-004)	-0.0783*** (0)	0.0224** (6.696183e-004)	-0.0523*** (1.776357e-015)	●	○	●	○	○	●	○	○
-0.1392*** (0)	-0.1441*** (0)	-0.0594*** (0)	-0.0713*** (0)	●	○	●	○	○	○	●	○
-0.2747*** (0)	-0.2680*** (0)	-0.1640*** (0)	-0.1623*** (0)	●	○	●	○	○	○	○	●
-0.0249** (1.896632e-004)	-0.0837*** (0)	0.0260*** (7.556495e-005)	-0.0532*** (5.551115e-016)	●	○	○	○	●	●	○	○
-0.1447*** (0)	-0.1558*** (0)	-0.0582*** (0)	-0.0782*** (0)	●	○	○	○	●	○	●	○
-0.2458*** (0)	-0.2400*** (0)	-0.1407*** (0)	-0.1411*** (0)	●	○	○	○	●	○	○	●
-0.0471*** (1.417755e-012)	-0.0994*** (0)	0.0051 (4.428822e-001)	-0.0700*** (0)	●	○	○	○	○	●	●	○
-0.0649*** (0)	-0.0590*** (0)	-0.0303*** (4.132117e-006)	-0.0323*** (8.915754e-007)	●	○	○	○	○	●	○	●
-0.1526*** (0)	-0.1484*** (0)	-0.0714*** (0)	-0.0717*** (0)	●	○	○	○	○	○	●	●
-0.1453*** (0)	-0.1038*** (0)	-0.1429*** (0)	-0.1048*** (0)	○	●	●	○	●	○	○	○
0.0041 (5.429556e-001)	-0.0586*** (0)	0.0408*** (5.600295e-010)	-0.0390*** (2.987577e-009)	○	●	●	○	○	●	○	○
-0.0649*** (0)	-0.0772*** (0)	-0.0425*** (1.055250e-010)	-0.0618*** (0)	○	●	●	○	○	○	●	○
-0.1396*** (0)	-0.1316*** (0)	-0.1272*** (0)	-0.1260*** (0)	○	●	●	○	○	○	○	●
-0.0032 (6.286277e-001)	-0.0666*** (0)	0.0369*** (2.066301e-008)	-0.0438*** (2.813849e-011)	○	●	○	○	●	●	○	○
-0.0762*** (0)	-0.0912*** (0)	-0.0496*** (4.918288e-014)	-0.0721*** (0)	○	●	○	○	●	○	●	○
-0.1247*** (0)	-0.1177*** (0)	-0.1127*** (0)	-0.1133*** (0)	○	●	○	○	●	○	○	●
-0.0302*** (5.737372e-006)	-0.0849*** (0)	0.0082 (2.102428e-001)	-0.0658*** (0)	○	●	○	○	○	●	●	○
-0.0614*** (0)	-0.0554*** (1.110223e-016)	-0.0378*** (9.577847e-009)	-0.0395*** (1.894558e-009)	○	●	○	○	○	●	○	●
-0.0900*** (0)	-0.0847*** (0)	-0.0684*** (0)	-0.0687*** (0)	○	●	○	○	○	○	●	●
0.0006 (9.275753e-001)	-0.0628*** (0)	0.0490*** (8.903989e-014)	-0.0316*** (1.564047e-006)	○	○	●	○	●	●	○	○
-0.1143*** (0)	-0.1352*** (0)	-0.0240** (2.587975e-004)	-0.0528*** (9.992007e-016)	○	○	●	○	●	○	●	○
-0.2241*** (0)	-0.2192*** (0)	-0.1102*** (0)	-0.1115*** (0)	○	○	●	○	●	○	○	●
-0.0261*** (8.666063e-005)	-0.0810*** (0)	0.0216* (1.058044e-003)	-0.0528*** (9.992007e-016)	○	○	●	○	○	●	●	○
-0.0560*** (0)	-0.0498*** (7.538414e-014)	-0.0265*** (5.811270e-005)	-0.0276*** (2.790343e-005)	○	○	●	○	○	●	○	●

-0.1311*** (0)	-0.1273*** (0)	-0.0473*** (6.380452e-013)	-0.0477*** (4.194423e-013)	○	○	●	○	○	○	●	●
-0.0287*** (1.585115e-005)	-0.0774*** (0)	0.0148 (2.482477e-002)	-0.0523*** (1.776357e-015)	○	○	○	○	●	●	●	○
-0.0616*** (0)	-0.0560*** (0)	-0.0301*** (4.871898e-006)	-0.0323*** (9.291490e-007)	○	○	○	○	●	●	○	●
-0.1411*** (0)	-0.1374*** (0)	-0.0562*** (0)	-0.0567*** (0)	○	○	○	○	●	○	●	●
-0.0816*** (0)	-0.0749*** (0)	-0.0511*** (7.993606e-015)	-0.0529*** (8.881784e-016)	○	○	○	○	○	●	●	●
-0.0975*** (0)	-0.0725*** (0)	-0.0869*** (0)	-0.0696*** (0)	●	●	●	○	●	○	○	○
-0.0142 (3.266694e-002)	-0.0712*** (0)	0.0265*** (5.735219e-005)	-0.0497*** (4.007905e-014)	●	●	●	○	○	●	○	○
-0.0520*** (5.218048e-015)	-0.0687*** (0)	-0.0241** (2.495600e-004)	-0.0509*** (1.065814e-014)	●	●	●	○	○	○	●	○
-0.1055*** (0)	-0.0981*** (0)	-0.0887*** (0)	-0.0889*** (0)	●	●	●	○	○	○	○	●
-0.0144 (3.097365e-002)	-0.0756*** (0)	0.0304*** (3.843354e-006)	-0.0503*** (1.965095e-014)	●	●	○	○	●	●	○	○
-0.0557*** (1.110223e-016)	-0.0769*** (0)	-0.0232** (4.236875e-004)	-0.0553*** (0)	●	●	○	○	●	○	●	○
-0.0897*** (0)	-0.0830*** (0)	-0.0753*** (0)	-0.0768*** (0)	●	●	○	○	●	○	○	●
-0.0332*** (6.119953e-007)	-0.0880*** (0)	0.0106 (1.072802e-001)	-0.0662*** (0)	●	●	○	○	○	●	●	○
-0.0508*** (2.409184e-014)	-0.0446*** (1.984568e-011)	-0.0256** (1.020780e-004)	-0.0277*** (2.531341e-005)	●	●	○	○	○	●	○	●
-0.0667*** (0)	-0.0615*** (0)	-0.0434*** (4.152145e-011)	-0.0444*** (1.420730e-011)	●	●	○	○	○	○	●	●
-0.0189* (4.483160e-003)	-0.0728*** (0)	0.0252** (1.303568e-004)	-0.0477*** (4.057865e-013)	●	○	●	○	●	●	○	○
-0.1394*** (0)	-0.1469*** (0)	-0.0584*** (0)	-0.0732*** (0)	●	○	●	○	●	○	●	○
-0.2427*** (0)	-0.2367*** (0)	-0.1395*** (0)	-0.1394*** (0)	●	○	●	○	●	○	○	●
-0.0408*** (8.593971e-010)	-0.0885*** (0)	0.0047 (4.760720e-001)	-0.0643*** (0)	●	○	●	○	○	●	●	○
-0.0581*** (0)	-0.0517*** (8.437695e-015)	-0.0280*** (2.122301e-005)	-0.0290*** (1.032217e-005)	●	○	●	○	○	●	○	●
-0.1461*** (0)	-0.1414*** (0)	-0.0689*** (0)	-0.0682*** (0)	●	○	●	○	○	○	●	●
-0.0394*** (3.266200e-009)	-0.0839*** (0)	0.0044 (5.049546e-001)	-0.0606*** (0)	●	○	○	○	●	●	●	○
-0.0600*** (0)	-0.0542*** (3.330669e-016)	-0.0264*** (5.981742e-005)	-0.0288*** (1.232489e-005)	●	○	○	○	●	●	○	●
-0.1547*** (0)	-0.1504*** (0)	-0.0727*** (0)	-0.0727*** (0)	●	○	○	○	●	○	●	●
-0.0776*** (0)	-0.0708*** (0)	-0.0435*** (3.764611e-011)	-0.0455*** (4.440559e-012)	●	○	○	○	○	●	●	●
0.0089 (1.809502e-001)	-0.0531*** (1.443290e-015)	0.0446*** (1.263878e-011)	-0.0340*** (2.347587e-007)	○	●	●	○	●	●	○	○
-0.0655*** (0)	-0.0792*** (0)	-0.0421*** (1.622309e-010)	-0.0628*** (0)	○	●	●	○	●	○	●	○
-0.1178*** (0)	-0.1105*** (0)	-0.1073*** (0)	-0.1074*** (0)	○	●	●	○	●	○	○	●
-0.0174* (8.886397e-003)	-0.0708*** (0)	0.0165 (1.212202e-002)	-0.0554*** (0)	○	●	●	○	○	●	●	○
-0.0487*** (2.566836e-013)	-0.0421*** (2.464055e-010)	-0.0287*** (1.337222e-005)	-0.0296*** (6.684758e-006)	○	●	●	○	○	●	○	●
-0.0789*** (0)	-0.0732*** (0)	-0.0600*** (0)	-0.0596*** (0)	○	●	●	○	○	○	●	●
-0.0212* (1.456697e-003)	-0.0684*** (0)	0.0096 (1.432256e-001)	-0.0554*** (0)	○	●	○	○	●	●	●	○
-0.0556*** (1.110223e-016)	-0.0497*** (8.082424e-014)	-0.0324*** (8.599219e-007)	-0.0345*** (1.576668e-007)	○	●	○	○	●	●	○	●
-0.0922*** (0)	-0.0869*** (0)	-0.0695*** (0)	-0.0697*** (0)	○	●	○	○	●	○	●	●
-0.0751*** (0)	-0.0681*** (0)	-0.0538*** (2.220446e-016)	-0.0555*** (0)	○	●	○	○	○	●	●	●

-0.0198* (2.965481e-003)	-0.0678*** (0)	0.0214* (1.163007e-003)	-0.0445*** (1.292788e-011)	○	○	●	○	●	●	●	○
-0.0512*** (1.454392e-014)	-0.0451*** (1.262823e-011)	-0.0219** (8.840907e-004)	-0.0234** (3.857765e-004)	○	○	●	○	●	●	○	●
-0.1338*** (0)	-0.1299*** (0)	-0.0496*** (4.463097e-014)	-0.0496*** (4.440892e-014)	○	○	●	○	●	○	●	●
-0.0706*** (0)	-0.0635*** (0)	-0.0424*** (1.205956e-010)	-0.0434*** (3.999900e-011)	○	○	●	○	○	●	●	●
-0.0688*** (0)	-0.0622*** (0)	-0.0416*** (2.588919e-010)	-0.0445*** (1.330480e-011)	○	○	○	○	●	●	●	●
-0.0101 (1.290997e-001)	-0.0661*** (0)	0.0290*** (1.041398e-005)	-0.0453*** (5.473288e-012)	●	●	●	○	●	●	○	○
-0.0528*** (2.109424e-015)	-0.0709*** (0)	-0.0238** (2.968444e-004)	-0.0519*** (2.997602e-015)	●	●	●	○	●	○	●	○
-0.0889*** (0)	-0.0820*** (0)	-0.0749*** (0)	-0.0763*** (0)	●	●	●	○	●	○	○	●
-0.0286*** (1.719959e-005)	-0.0786*** (0)	0.0097 (1.393480e-001)	-0.0610*** (0)	●	●	●	○	○	●	●	○
-0.0456*** (7.056578e-012)	-0.0389*** (4.916138e-009)	-0.0238** (3.032347e-004)	-0.0250** (1.484661e-004)	●	●	●	○	○	●	○	●
-0.0632*** (0)	-0.0577*** (0)	-0.0420*** (1.697060e-010)	-0.0424*** (1.178923e-010)	●	●	●	○	○	○	●	●
-0.0256** (1.243712e-004)	-0.0723*** (0)	0.0098 (1.358280e-001)	-0.0569*** (0)	●	●	○	○	●	●	●	○
-0.0465*** (2.918554e-012)	-0.0405*** (1.183725e-009)	-0.0220** (8.282663e-004)	-0.0245** (2.023009e-004)	●	●	○	○	●	●	○	●
-0.0686*** (0)	-0.0635*** (0)	-0.0443*** (1.632749e-011)	-0.0452*** (6.450396e-012)	●	●	○	○	●	○	●	●
-0.0612*** (0)	-0.0542*** (3.330669e-016)	-0.0382*** (6.656590e-009)	-0.0403*** (8.873384e-010)	●	●	○	○	○	●	●	●
-0.0344*** (2.444366e-007)	-0.0752*** (0)	0.0041 (5.325417e-001)	-0.0561*** (0)	●	○	●	○	●	●	●	○
-0.0537*** (6.661338e-016)	-0.0474*** (1.079803e-012)	-0.0243** (2.156504e-004)	-0.0257*** (9.247749e-005)	●	○	●	○	●	●	○	●
-0.1481*** (0)	-0.1433*** (0)	-0.0701*** (0)	-0.0692*** (0)	●	○	●	○	●	○	●	●
-0.0712*** (0)	-0.0639*** (0)	-0.0412*** (3.702989e-010)	-0.0423*** (1.225859e-010)	●	○	●	○	○	●	●	●
-0.0669*** (0)	-0.0602*** (0)	-0.0374*** (1.374435e-008)	-0.0404*** (7.900589e-010)	●	○	○	○	●	●	●	●
-0.0110 (9.771876e-002)	-0.0574*** (0)	0.0163 (1.314278e-002)	-0.0472*** (7.156498e-013)	○	●	●	○	●	●	●	○
-0.0439*** (4.343537e-011)	-0.0375*** (1.816710e-008)	-0.0241** (2.522646e-004)	-0.0254** (1.113920e-004)	○	●	●	○	●	●	○	●
-0.0810*** (0)	-0.0752*** (0)	-0.0611*** (0)	-0.0606*** (0)	○	●	●	○	●	○	●	●
-0.0627*** (0)	-0.0552*** (1.110223e-016)	-0.0449*** (8.408163e-012)	-0.0459*** (3.071654e-012)	○	●	●	○	○	●	●	●
-0.0618*** (0)	-0.0549*** (1.110223e-016)	-0.0444*** (1.448142e-011)	-0.0472*** (6.912249e-013)	○	●	○	○	●	●	●	●
-0.0602*** (0)	-0.0532*** (1.221245e-015)	-0.0347*** (1.333095e-007)	-0.0370*** (1.833220e-008)	○	○	●	○	●	●	●	●
-0.0220** (9.633955e-004)	-0.0649*** (0)	0.0091 (1.659113e-001)	-0.0529*** (8.881784e-016)	●	●	●	○	●	●	●	○
-0.0417*** (3.637880e-010)	-0.0352*** (1.265127e-007)	-0.0204* (1.920650e-003)	-0.0219** (8.621080e-004)	●	●	●	○	●	●	○	●
-0.0651*** (0)	-0.0596*** (0)	-0.0430*** (6.525058e-011)	-0.0431*** (5.520273e-011)	●	●	●	○	●	○	●	●
-0.0564*** (0)	-0.0489*** (2.086109e-013)	-0.0364*** (3.186208e-008)	-0.0377*** (1.015541e-008)	●	●	●	○	○	●	●	●
-0.0510*** (1.776357e-014)	-0.0441*** (3.593537e-011)	-0.0322*** (9.746785e-007)	-0.0354*** (7.213633e-008)	●	●	○	○	●	●	●	●
-0.0617*** (0)	-0.0546*** (2.220446e-016)	-0.0356*** (6.553237e-008)	-0.0379*** (8.785629e-009)	●	○	●	○	●	●	●	●
-0.0519*** (6.217249e-015)	-0.0446*** (2.046641e-011)	-0.0373*** (1.383337e-008)	-0.0395*** (1.903878e-009)	○	●	●	○	●	●	●	●
-0.0472*** (1.297296e-012)	-0.0398*** (2.205825e-009)	-0.0309*** (2.740027e-006)	-0.0333*** (4.074510e-007)	●	●	●	○	●	●	●	●
-0.0817*** (1.187098e-034)	-0.0574*** (1.642633e-006)	-0.0813*** (4.202497e-035)	-0.0506*** (1.685221e-010)	●	●	●	○	●	●	●	●

**D_x and θ_{neu} (and P_{neu})**											
θ_{neu} (P)	P_{neu} (P)	θ_{neu} (W)	P_{neu} (W)	RR	GC	RD	D_n	D_x	FD_n	FD_x	d_{neu}
-0.1655*** (0)	-0.1552*** (0)	-0.1143*** (0)	-0.1075*** (0)	○	○	○	○	○	○	○	○
-0.1595*** (0)	-0.1507*** (0)	-0.1033*** (0)	-0.0990*** (0)	●	○	○	○	○	○	○	○
-0.1200*** (0)	-0.1140*** (0)	-0.0996*** (0)	-0.0996*** (0)	○	●	○	○	○	○	○	○
-0.1602*** (0)	-0.1487*** (0)	-0.1100*** (0)	-0.1016*** (0)	○	○	●	○	○	○	○	○
-0.0840*** (0)	-0.0872*** (0)	-0.0612*** (0)	-0.0696*** (0)	○	○	○	●	○	○	○	○
-0.0763*** (0)	-0.0907*** (0)	-0.0498*** (3.619327e-014)	-0.0725*** (0)	○	○	○	○	○	●	○	○
-0.0144 (3.109781e-002)	-0.0565*** (0)	0.0059 (3.726204e-001)	-0.0452*** (6.628476e-012)	○	○	○	○	○	○	●	○
-0.1596*** (0)	-0.1561*** (0)	-0.1126*** (0)	-0.1079*** (0)	○	○	○	○	○	○	○	●
-0.0828*** (0)	-0.0877*** (0)	-0.0566*** (0)	-0.0712*** (0)	●	●	○	○	○	○	○	○
-0.1575*** (0)	-0.1465*** (0)	-0.1039*** (0)	-0.0965*** (0)	●	○	●	○	○	○	○	○
-0.0709*** (0)	-0.0789*** (0)	-0.0417*** (2.331193e-010)	-0.0566*** (0)	●	○	○	●	○	○	○	○
-0.0648*** (0)	-0.0838*** (0)	-0.0318*** (1.369661e-006)	-0.0610*** (0)	●	○	○	○	○	●	○	○
-0.0060 (3.702684e-001)	-0.0520*** (5.551115e-015)	0.0202* (2.136569e-003)	-0.0373*** (1.403839e-008)	●	○	○	○	○	○	●	○
-0.1564*** (0)	-0.1529*** (0)	-0.1049*** (0)	-0.1002*** (0)	●	○	○	○	○	○	○	●
-0.1117*** (0)	-0.1042*** (0)	-0.0940*** (0)	-0.0921*** (0)	○	●	●	○	○	○	○	○
-0.0817*** (0)	-0.0850*** (0)	-0.0617*** (0)	-0.0703*** (0)	○	●	○	●	○	○	○	○
-0.0758*** (0)	-0.0904*** (0)	-0.0505*** (1.676437e-014)	-0.0728*** (0)	○	●	○	○	○	●	○	○
-0.0268*** (5.664814e-005)	-0.0713*** (0)	0.0097 (1.389139e-001)	-0.0433*** (4.775758e-011)	○	●	○	○	○	○	●	○
-0.1190*** (0)	-0.1152*** (0)	-0.1047*** (0)	-0.0997*** (0)	○	●	○	○	○	○	○	●
-0.0786*** (0)	-0.0805*** (0)	-0.0574*** (0)	-0.0642*** (0)	○	○	●	●	○	○	○	○
-0.0683*** (0)	-0.0816*** (0)	-0.0443*** (1.692602e-011)	-0.0652*** (0)	○	○	●	○	○	●	○	○
-0.0033 (6.246756e-001)	-0.0441*** (3.349454e-011)	0.0135 (4.053792e-002)	-0.0357*** (5.873729e-008)	○	○	●	○	○	○	●	○
-0.1530*** (0)	-0.1493*** (0)	-0.1071*** (0)	-0.1018*** (0)	○	○	●	○	○	○	○	●
-0.0752*** (0)	-0.0849*** (0)	-0.0532*** (5.551115e-016)	-0.0687*** (0)	○	○	○	●	○	●	○	○
-0.0209* (1.728800e-003)	-0.0645*** (0)	0.0044 (5.035508e-001)	-0.0481*** (2.601253e-013)	○	○	○	●	○	○	●	○
-0.0889*** (0)	-0.0868*** (0)	-0.0739*** (0)	-0.0688*** (0)	○	○	○	●	○	○	○	●
-0.0430*** (1.014443e-010)	-0.0825*** (0)	-0.0047 (4.711421e-001)	-0.0522*** (1.998401e-015)	○	○	○	○	○	●	●	○
-0.0891*** (0)	-0.0864*** (0)	-0.0743*** (0)	-0.0696*** (0)	○	○	○	○	○	●	○	●
-0.0452*** (1.097189e-011)	-0.0438*** (4.717582e-011)	-0.0425*** (9.966694e-011)	-0.0376*** (1.083488e-008)	○	○	○	○	○	○	●	●
-0.0815*** (0)	-0.0842*** (0)	-0.0575*** (0)	-0.0693*** (0)	●	●	●	○	○	○	○	○
-0.0595*** (0)	-0.0696*** (0)	-0.0359*** (4.678740e-008)	-0.0538*** (2.220446e-016)	●	●	○	●	○	○	○	○
-0.0580*** (0)	-0.0780*** (0)	-0.0288*** (1.187843e-005)	-0.0589*** (0)	●	●	○	○	○	●	○	○
-0.0273*** (4.046523e-005)	-0.0725*** (0)	0.0125 (5.698302e-002)	-0.0429*** (6.827972e-011)	●	●	○	○	○	○	●	○

-0.0881*** (0)	-0.0846*** (0)	-0.0708*** (0)	-0.0665*** (0)	●	●	○	○	○	○	○	●
-0.0694*** (0)	-0.0754*** (0)	-0.0425*** (1.080873e-010)	-0.0548*** (1.110223e-016)	●	○	●	●	○	○	○	○
-0.0617*** (0)	-0.0780*** (0)	-0.0322*** (9.558635e-007)	-0.0581*** (0)	●	○	●	○	○	●	○	○
-0.0006 (9.300263e-001)	-0.0431*** (9.971157e-011)	0.0202* (2.140405e-003)	-0.0328*** (6.275763e-007)	●	○	●	○	○	○	●	○
-0.1523*** (0)	-0.1486*** (0)	-0.1034*** (0)	-0.0981*** (0)	●	○	●	○	○	○	○	●
-0.0626*** (0)	-0.0770*** (0)	-0.0340*** (2.415488e-007)	-0.0562*** (0)	●	○	○	●	○	●	○	○
-0.0132 (4.687862e-002)	-0.0603*** (0)	0.0176* (7.581405e-003)	-0.0410*** (4.809830e-010)	●	○	○	●	○	○	●	○
-0.0779*** (0)	-0.0761*** (0)	-0.0566*** (0)	-0.0519*** (3.219647e-015)	●	○	○	●	○	○	○	●
-0.0393*** (3.463579e-009)	-0.0804*** (0)	0.0034 (6.061614e-001)	-0.0481*** (2.567946e-013)	●	○	○	○	○	●	●	○
-0.0764*** (0)	-0.0740*** (0)	-0.0549*** (1.110223e-016)	-0.0506*** (1.498801e-014)	●	○	○	○	○	●	○	●
-0.0310*** (3.115864e-006)	-0.0301*** (6.326553e-006)	-0.0211* (1.361606e-003)	-0.0166 (1.164243e-002)	●	○	○	○	○	○	●	●
-0.0754*** (0)	-0.0773*** (0)	-0.0578*** (0)	-0.0648*** (0)	○	●	●	●	○	○	○	○
-0.0673*** (0)	-0.0806*** (0)	-0.0451*** (6.884271e-012)	-0.0656*** (0)	○	●	●	○	○	●	○	○
-0.0156 (1.940278e-002)	-0.0589*** (0)	0.0169 (1.033297e-002)	-0.0343*** (1.904372e-007)	○	●	●	○	○	○	●	○
-0.1095*** (0)	-0.1053*** (0)	-0.0978*** (0)	-0.0922*** (0)	○	●	●	○	○	○	○	●
-0.0753*** (0)	-0.0852*** (0)	-0.0535*** (4.440892e-016)	-0.0689*** (0)	○	●	○	●	○	●	○	○
-0.0287*** (1.599764e-005)	-0.0738*** (0)	0.0088 (1.809125e-001)	-0.0447*** (1.029432e-011)	○	●	○	●	○	○	●	○
-0.0867*** (0)	-0.0845*** (0)	-0.0746*** (0)	-0.0696*** (0)	○	●	○	●	○	○	○	●
-0.0430*** (1.084549e-010)	-0.0828*** (0)	-0.0049 (4.534169e-001)	-0.0523*** (1.776357e-015)	○	●	○	○	○	●	●	○
-0.0892*** (0)	-0.0865*** (0)	-0.0745*** (0)	-0.0697*** (0)	○	●	○	○	○	●	○	●
-0.0603*** (0)	-0.0593*** (0)	-0.0400*** (1.175365e-009)	-0.0352*** (8.714715e-008)	○	●	○	○	○	○	●	●
-0.0682*** (0)	-0.0770*** (0)	-0.0481*** (2.643441e-013)	-0.0625*** (0)	○	○	●	●	○	●	○	○
-0.0100 (1.323235e-001)	-0.0524*** (3.330669e-015)	0.0121 (6.489105e-002)	-0.0386*** (4.611154e-009)	○	○	●	●	○	○	●	○
-0.0827*** (0)	-0.0804*** (0)	-0.0690*** (0)	-0.0636*** (0)	○	○	●	●	○	○	○	●
-0.0318*** (1.827717e-006)	-0.0699*** (0)	0.0032 (6.271274e-001)	-0.0423*** (1.339809e-010)	○	○	●	○	○	●	●	○
-0.0807*** (0)	-0.0777*** (0)	-0.0679*** (0)	-0.0627*** (0)	○	○	●	○	○	●	○	●
-0.0341*** (3.005027e-007)	-0.0323*** (1.214234e-006)	-0.0343*** (1.836093e-007)	-0.0287*** (1.331191e-005)	○	○	●	○	○	○	●	●
-0.0356*** (8.847215e-008)	-0.0636*** (0)	-0.0080 (2.242484e-001)	-0.0389*** (3.246991e-009)	○	○	○	●	○	●	●	○
-0.0849*** (0)	-0.0825*** (0)	-0.0719*** (0)	-0.0670*** (0)	○	○	○	●	○	●	○	●
-0.0545*** (2.220446e-016)	-0.0528*** (2.109424e-015)	-0.0458*** (3.263945e-012)	-0.0409*** (4.937624e-010)	○	○	○	●	○	○	●	●
-0.0715*** (0)	-0.0701*** (0)	-0.0501*** (2.531308e-014)	-0.0450*** (7.691403e-012)	○	○	○	○	○	●	●	●
-0.0582*** (0)	-0.0663*** (0)	-0.0368*** (2.231459e-008)	-0.0521*** (2.220446e-015)	●	●	●	●	○	○	○	○
-0.0558*** (0)	-0.0730*** (0)	-0.0296*** (7.062731e-006)	-0.0563*** (0)	●	●	●	○	○	●	○	○
-0.0234** (4.434050e-004)	-0.0644*** (0)	0.0118 (7.246518e-002)	-0.0385*** (4.952221e-009)	●	●	●	○	○	○	●	○
-0.0859*** (0)	-0.0822*** (0)	-0.0702*** (0)	-0.0654*** (0)	●	●	●	○	○	○	○	●

-0.0567*** (0)	-0.0719*** (0)	-0.0314*** (1.860310e-006)	-0.0544*** (1.110223e-016)	●	●	○	●	○	●	○	○
-0.0287*** (1.598294e-005)	-0.0746*** (0)	0.0121 (6.660073e-002)	-0.0441*** (2.112566e-011)	●	●	○	●	○	○	●	○
-0.0662*** (0)	-0.0642*** (0)	-0.0522*** (2.220446e-015)	-0.0475*** (4.934941e-013)	●	●	○	●	○	○	○	●
-0.0374*** (2.014431e-008)	-0.0795*** (0)	0.0046 (4.887530e-001)	-0.0474*** (6.068479e-013)	●	●	○	○	○	●	●	○
-0.0688*** (0)	-0.0663*** (0)	-0.0521*** (2.331468e-015)	-0.0478*** (3.734790e-013)	●	●	○	○	○	●	○	●
-0.0483*** (4.138911e-013)	-0.0473*** (1.142642e-012)	-0.0265*** (5.582365e-005)	-0.0219** (8.733917e-004)	●	●	○	○	○	○	●	●
-0.0601*** (0)	-0.0725*** (0)	-0.0342*** (1.952699e-007)	-0.0540*** (2.220446e-016)	●	○	●	●	○	●	○	○
-0.0087 (1.919240e-001)	-0.0520*** (5.773160e-015)	0.0171* (9.329207e-003)	-0.0367*** (2.414388e-008)	●	○	●	●	○	○	●	○
-0.0755*** (0)	-0.0735*** (0)	-0.0559*** (0)	-0.0508*** (1.110223e-014)	●	○	●	●	○	○	○	●
-0.0339*** (3.484631e-007)	-0.0710*** (0)	0.0031 (6.397438e-001)	-0.0432*** (5.250334e-011)	●	○	●	○	○	●	●	○
-0.0728*** (0)	-0.0701*** (0)	-0.0538*** (3.330669e-016)	-0.0489*** (1.031397e-013)	●	○	●	○	○	●	○	●
-0.0257** (1.126598e-004)	-0.0243** (2.575435e-004)	-0.0192* (3.484240e-003)	-0.0141 (3.234624e-002)	●	○	●	○	○	○	●	●
-0.0296*** (8.761899e-006)	-0.0601*** (0)	0.0023 (7.273902e-001)	-0.0330*** (5.275221e-007)	●	○	○	●	○	●	●	○
-0.0723*** (0)	-0.0703*** (0)	-0.0529*** (8.881784e-016)	-0.0484*** (1.942890e-013)	●	○	○	●	○	●	○	●
-0.0403*** (1.430225e-009)	-0.0390*** (4.575767e-009)	-0.0250** (1.489299e-004)	-0.0205* (1.853513e-003)	●	○	○	●	○	○	●	●
-0.0607*** (0)	-0.0596*** (0)	-0.0337*** (2.945023e-007)	-0.0289*** (1.106765e-005)	●	○	○	○	○	●	●	●
-0.0678*** (0)	-0.0767*** (0)	-0.0485*** (1.575406e-013)	-0.0628*** (0)	○	●	●	●	○	●	○	○
-0.0177* (7.866727e-003)	-0.0616*** (0)	0.0158 (1.660715e-002)	-0.0360*** (4.467060e-008)	○	●	●	●	○	○	●	○
-0.0796*** (0)	-0.0771*** (0)	-0.0697*** (0)	-0.0642*** (0)	○	●	●	●	○	○	○	●
-0.0310*** (3.211497e-006)	-0.0694*** (0)	0.0027 (6.833076e-001)	-0.0425*** (1.022292e-010)	○	●	●	○	○	●	●	○
-0.0802*** (0)	-0.0772*** (0)	-0.0682*** (0)	-0.0629*** (0)	○	●	●	○	○	●	○	●
-0.0492*** (1.407763e-013)	-0.0478*** (6.890044e-013)	-0.0323*** (9.103352e-007)	-0.0268*** (4.638674e-005)	○	●	●	○	○	○	●	●
-0.0372*** (2.303144e-008)	-0.0658*** (0)	-0.0070 (2.866385e-001)	-0.0384*** (5.540990e-009)	○	●	○	●	○	●	●	○
-0.0853*** (0)	-0.0830*** (0)	-0.0719*** (0)	-0.0670*** (0)	○	●	○	●	○	●	○	●
-0.0636*** (0)	-0.0623*** (0)	-0.0419*** (1.826149e-010)	-0.0371*** (1.672531e-008)	○	●	○	●	○	○	●	●
-0.0729*** (0)	-0.0715*** (0)	-0.0498*** (3.796963e-014)	-0.0447*** (1.102152e-011)	○	●	○	○	○	●	●	●
-0.0268*** (5.731823e-005)	-0.0539*** (5.551115e-016)	-0.0016 (8.041300e-001)	-0.0314*** (1.890018e-006)	○	○	●	●	○	●	●	○
-0.0775*** (0)	-0.0748*** (0)	-0.0663*** (0)	-0.0609*** (0)	○	○	●	●	○	●	○	●
-0.0436*** (6.022682e-011)	-0.0415*** (4.739324e-010)	-0.0375*** (1.236654e-008)	-0.0318*** (1.330156e-006)	○	○	●	●	○	○	●	●
-0.0602*** (0)	-0.0584*** (0)	-0.0415*** (2.968858e-010)	-0.0356*** (6.390405e-008)	○	○	●	○	○	●	●	●
-0.0564*** (0)	-0.0564*** (0)	-0.0404*** (8.310490e-010)	-0.0348*** (1.245115e-007)	○	○	○	●	○	●	●	●
-0.0549*** (1.110223e-016)	-0.0681*** (0)	-0.0318*** (1.300957e-006)	-0.0525*** (1.554312e-015)	●	●	●	●	○	●	○	○
-0.0250** (1.688333e-004)	-0.0667*** (0)	0.0112 (8.849886e-002)	-0.0398*** (1.389858e-009)	●	●	●	●	○	○	●	○
-0.0643*** (0)	-0.0621*** (0)	-0.0516*** (4.107825e-015)	-0.0467*** (1.271538e-012)	●	●	●	●	○	○	○	●
-0.0334*** (5.413473e-007)	-0.0712*** (0)	0.0038 (5.666471e-001)	-0.0428*** (7.904222e-011)	●	●	●	○	○	●	●	○

-0.0661*** (0)	-0.0633*** (0)	-0.0512*** (6.772360e-015)	-0.0464*** (1.663447e-012)	●	●	●	○	○	●	○	●
-0.0439*** (4.063505e-011)	-0.0426*** (1.579347e-010)	-0.0250** (1.483296e-004)	-0.0196* (2.898184e-003)	●	●	●	○	○	○	●	●
-0.0308*** (3.863429e-006)	-0.0617*** (0)	0.0022 (7.400039e-001)	-0.0331*** (4.767778e-007)	●	●	○	●	○	●	●	○
-0.0657*** (0)	-0.0636*** (0)	-0.0505*** (1.709743e-014)	-0.0460*** (2.770117e-012)	●	●	○	●	○	●	○	●
-0.0510*** (1.920686e-014)	-0.0498*** (7.160939e-014)	-0.0280*** (2.131795e-005)	-0.0234** (3.801964e-004)	●	●	○	●	○	○	●	●
-0.0569*** (0)	-0.0557*** (0)	-0.0323*** (9.521784e-007)	-0.0275*** (3.012502e-005)	●	●	○	○	○	●	●	●
-0.0258** (1.083107e-004)	-0.0534*** (9.992007e-016)	0.0021 (7.508942e-001)	-0.0297*** (6.458440e-006)	●	○	●	●	○	●	●	○
-0.0694*** (0)	-0.0670*** (0)	-0.0520*** (2.775558e-015)	-0.0470*** (8.605339e-013)	●	○	●	●	○	●	○	●
-0.0354*** (1.097858e-007)	-0.0336*** (4.380518e-007)	-0.0233** (3.894248e-004)	-0.0181* (5.841608e-003)	●	○	●	●	○	○	●	●
-0.0550*** (1.110223e-016)	-0.0534*** (9.992007e-016)	-0.0317*** (1.413272e-006)	-0.0261*** (7.233772e-005)	●	○	●	○	○	●	●	●
-0.0463*** (3.629763e-012)	-0.0465*** (2.862155e-012)	-0.0253** (1.210152e-004)	-0.0199* (2.441642e-003)	●	○	○	●	○	●	●	●
-0.0279*** (2.751705e-005)	-0.0556*** (1.110223e-016)	-0.0010 (8.780467e-001)	-0.0310*** (2.422411e-006)	○	●	●	●	○	●	●	○
-0.0774*** (0)	-0.0747*** (0)	-0.0665*** (0)	-0.0611*** (0)	○	●	●	●	○	●	○	●
-0.0526*** (2.775558e-015)	-0.0509*** (2.020606e-014)	-0.0344*** (1.713294e-007)	-0.0289*** (1.148894e-005)	○	●	●	●	○	○	●	●
-0.0608*** (0)	-0.0591*** (0)	-0.0413*** (3.291375e-010)	-0.0355*** (6.981161e-008)	○	●	●	○	○	●	●	●
-0.0591*** (0)	-0.0591*** (0)	-0.0394*** (2.093680e-009)	-0.0338*** (2.714768e-007)	○	●	○	●	○	●	●	●
-0.0474*** (1.013634e-012)	-0.0470*** (1.579403e-012)	-0.0336*** (3.314300e-007)	-0.0274*** (3.205953e-005)	○	○	●	●	○	●	●	●
-0.0279*** (2.827738e-005)	-0.0558*** (0)	0.0016 (8.021761e-001)	-0.0300*** (5.114062e-006)	●	●	●	●	○	●	●	○
-0.0635*** (0)	-0.0610*** (0)	-0.0498*** (3.874678e-014)	-0.0449*** (8.908763e-012)	●	●	●	●	○	●	○	●
-0.0468*** (2.162936e-012)	-0.0451*** (1.190703e-011)	-0.0265*** (5.512333e-005)	-0.0212* (1.290702e-003)	●	●	●	●	○	○	●	●
-0.0524*** (3.441691e-015)	-0.0508*** (2.320366e-014)	-0.0306*** (3.230856e-006)	-0.0250** (1.419645e-004)	●	●	●	○	○	●	●	●
-0.0457*** (6.504353e-012)	-0.0460*** (5.080047e-012)	-0.0249** (1.503562e-004)	-0.0196* (2.916063e-003)	●	●	○	●	○	●	●	●
-0.0420*** (2.817238e-010)	-0.0418*** (3.315348e-010)	-0.0239** (2.811144e-004)	-0.0179* (6.452274e-003)	●	○	●	●	○	●	●	●
-0.0496*** (8.770762e-014)	-0.0493*** (1.249001e-013)	-0.0329*** (5.626174e-007)	-0.0267*** (4.869785e-005)	○	●	●	●	○	●	●	●
-0.0423*** (2.059104e-010)	-0.0422*** (2.411454e-010)	-0.0238** (2.993361e-004)	-0.0178* (6.803477e-003)	●	●	●	●	○	●	●	●
-0.0593*** (5.133121e-019)	-0.0497*** (1.090322e-008)	-0.0436*** (3.323341e-011)	-0.0348*** (1.472559e-005)	●	●	●	●	○	●	●	●
**FD_n and θ_{neu} (and P_{neu})**											
θ_{neu} (P)	P_{neu} (P)	θ_{neu} (W)	P_{neu} (W)	RR	GC	RD	D_n	D_x	FD_n	FD_x	d_{neu}
-0.3210*** (0)	-0.2363*** (0)	-0.2247*** (0)	-0.1290*** (0)	○	○	○	○	○	○	○	○
-0.3351*** (0)	-0.2420*** (0)	-0.2431*** (0)	-0.1360*** (0)	●	○	○	○	○	○	○	○
-0.2014*** (0)	-0.1123*** (0)	-0.2179*** (0)	-0.1239*** (0)	○	●	○	○	○	○	○	○
-0.3255*** (0)	-0.2417*** (0)	-0.2265*** (0)	-0.1313*** (0)	○	○	●	○	○	○	○	○
-0.1361*** (0)	-0.0363*** (5.069871e-008)	-0.1341*** (0)	-0.0154 (1.892989e-002)	○	○	○	●	○	○	○	○
-0.2884*** (0)	-0.2013*** (0)	-0.2007*** (0)	-0.1017*** (0)	○	○	○	○	●	○	○	○
-0.1221*** (0)	-0.1050*** (0)	-0.0471*** (7.851497e-013)	-0.0257*** (9.181020e-005)	○	○	○	○	○	○	●	○

-0.2566*** (0)	-0.2532*** (0)	-0.1386*** (0)	-0.1376*** (0)	○	○	○	○	○	○	○	●
-0.1236*** (0)	-0.0544*** (3.330669e-016)	-0.1349*** (0)	-0.0643*** (0)	●	●	○	○	○	○	○	○
-0.3364*** (0)	-0.2450*** (0)	-0.2430*** (0)	-0.1367*** (0)	●	○	●	○	○	○	○	○
-0.1313*** (0)	-0.0313*** (2.614180e-006)	-0.1307*** (0)	-0.0085 (1.943310e-001)	●	○	○	●	○	○	○	○
-0.3048*** (0)	-0.2084*** (0)	-0.2234*** (0)	-0.1117*** (0)	●	○	○	○	●	○	○	○
-0.1447*** (0)	-0.1171*** (0)	-0.0768*** (0)	-0.0435*** (3.819733e-011)	●	○	○	○	○	○	●	○
-0.2805*** (0)	-0.2762*** (0)	-0.1699*** (0)	-0.1678*** (0)	●	○	○	○	○	○	○	●
-0.2010*** (0)	-0.1111*** (0)	-0.2167*** (0)	-0.1219*** (0)	○	●	●	○	○	○	○	○
-0.1036*** (0)	0.0000 (9.945409e-001)	-0.1468*** (0)	-0.0259*** (8.204116e-005)	○	●	○	●	○	○	○	○
-0.1793*** (0)	-0.0883*** (0)	-0.2010*** (0)	-0.1037*** (0)	○	●	○	○	●	○	○	○
-0.0728*** (0)	-0.0462*** (4.092615e-012)	-0.0706*** (0)	-0.0384*** (5.280135e-009)	○	●	○	○	○	○	●	○
-0.1375*** (0)	-0.1323*** (0)	-0.1354*** (0)	-0.1335*** (0)	○	●	○	○	○	○	○	●
-0.1479*** (0)	-0.0499*** (6.317169e-014)	-0.1420*** (0)	-0.0256*** (9.980479e-005)	○	○	●	●	○	○	○	○
-0.2944*** (0)	-0.2087*** (0)	-0.2039*** (0)	-0.1060*** (0)	○	○	●	○	●	○	○	○
-0.1220*** (0)	-0.1049*** (0)	-0.0462*** (2.231326e-012)	-0.0244** (2.063289e-004)	○	○	●	○	○	○	●	○
-0.2598*** (0)	-0.2565*** (0)	-0.1397*** (0)	-0.1388*** (0)	○	○	●	○	○	○	○	●
-0.1310*** (0)	-0.0303*** (5.331657e-006)	-0.1307*** (0)	-0.0111 (9.296586e-002)	○	○	○	●	●	○	○	○
-0.0423*** (1.992064e-010)	0.0132 (4.764139e-002)	-0.0385*** (4.957238e-009)	0.0357*** (5.742784e-008)	○	○	○	●	○	○	●	○
-0.0572*** (0)	-0.0607*** (0)	-0.0295*** (7.348427e-006)	-0.0274*** (3.169148e-005)	○	○	○	●	○	○	○	●
-0.1286*** (0)	-0.1208*** (0)	-0.0470*** (9.044987e-013)	-0.0367*** (2.336547e-008)	○	○	○	○	●	○	●	○
-0.2215*** (0)	-0.2188*** (0)	-0.1100*** (0)	-0.1104*** (0)	○	○	○	○	●	○	○	●
-0.1114*** (0)	-0.1117*** (0)	-0.0294*** (7.832342e-006)	-0.0292*** (9.057761e-006)	○	○	○	○	○	○	●	●
-0.1259*** (0)	-0.0600*** (0)	-0.1341*** (0)	-0.0669*** (0)	●	●	●	○	○	○	○	○
-0.0532*** (1.221245e-015)	0.0377*** (1.539470e-008)	-0.0982*** (0)	0.0102 (1.215075e-001)	●	●	○	●	○	○	○	○
-0.1087*** (0)	-0.0365*** (4.174373e-008)	-0.1259*** (0)	-0.0504*** (1.731948e-014)	●	●	○	○	●	○	○	○
-0.0439*** (4.346523e-011)	-0.0249** (1.825815e-004)	-0.0388*** (3.560083e-009)	-0.0163 (1.312594e-002)	●	●	○	○	○	○	●	○
-0.0946*** (0)	-0.0898*** (0)	-0.0897*** (0)	-0.0889*** (0)	●	●	○	○	○	○	○	●
-0.1366*** (0)	-0.0424*** (1.836803e-010)	-0.1294*** (0)	-0.0139 (3.518928e-002)	●	○	●	●	○	○	○	○
-0.3067*** (0)	-0.2127*** (0)	-0.2231*** (0)	-0.1132*** (0)	●	○	●	○	●	○	○	○
-0.1430*** (0)	-0.1142*** (0)	-0.0769*** (0)	-0.0414*** (3.122423e-010)	●	○	●	○	○	○	●	○
-0.2790*** (0)	-0.2746*** (0)	-0.1691*** (0)	-0.1667*** (0)	●	○	●	○	○	○	○	●
-0.1271*** (0)	-0.0260*** (9.335412e-005)	-0.1284*** (0)	-0.0051 (4.415618e-001)	●	○	○	●	●	○	○	○
-0.0486*** (2.690070e-013)	0.0103 (1.216628e-001)	-0.0495*** (5.362377e-014)	0.0309*** (2.724924e-006)	●	○	○	●	○	○	●	○
-0.0720*** (0)	-0.0750*** (0)	-0.0486*** (1.461054e-013)	-0.0458*** (3.238965e-012)	●	○	○	●	○	○	○	●
-0.1497*** (0)	-0.1320*** (0)	-0.0742*** (0)	-0.0530*** (7.771561e-016)	●	○	○	○	●	○	●	○

-0.2472*** (0)	-0.2436*** (0)	-0.1450*** (0)	-0.1443*** (0)	●	○	○	○	●	○	○	●
-0.1326*** (0)	-0.1322*** (0)	-0.0567*** (0)	-0.0557*** (0)	●	○	○	○	○	○	●	●
-0.1139*** (0)	-0.0114 (8.685437e-002)	-0.1527*** (0)	-0.0336*** (3.235487e-007)	○	●	●	●	○	○	○	○
-0.1808*** (0)	-0.0895*** (0)	-0.2010*** (0)	-0.1034*** (0)	○	●	●	○	●	○	○	○
-0.0695*** (0)	-0.0420*** (2.690772e-010)	-0.0679*** (0)	-0.0347*** (1.345486e-007)	○	●	●	○	○	○	●	○
-0.1346*** (0)	-0.1292*** (0)	-0.1325*** (0)	-0.1304*** (0)	○	●	●	○	○	○	○	●
-0.0986*** (0)	0.0058 (3.850198e-001)	-0.1436*** (0)	-0.0217** (9.545061e-004)	○	●	○	●	●	○	○	○
-0.0225** (7.160631e-004)	0.0369*** (2.854014e-008)	-0.0509*** (1.021405e-014)	0.0270*** (4.004719e-005)	○	●	○	●	○	○	●	○
-0.0222** (8.665557e-004)	-0.0244** (2.493077e-004)	-0.0410*** (4.620850e-010)	-0.0385*** (4.905915e-009)	○	●	○	●	○	○	○	●
-0.0801*** (0)	-0.0625*** (0)	-0.0701*** (0)	-0.0484*** (1.842970e-013)	○	●	○	○	●	○	●	○
-0.1128*** (0)	-0.1083*** (0)	-0.1138*** (0)	-0.1131*** (0)	○	●	○	○	●	○	○	●
-0.0555*** (1.110223e-016)	-0.0543*** (3.330669e-016)	-0.0447*** (1.114442e-011)	-0.0438*** (2.847822e-011)	○	●	○	○	○	○	●	●
-0.1428*** (0)	-0.0440*** (3.857314e-011)	-0.1386*** (0)	-0.0212* (1.244980e-003)	○	○	●	●	●	○	○	○
-0.0512*** (1.365574e-014)	0.0034 (6.110572e-001)	-0.0445*** (1.272948e-011)	0.0284*** (1.583246e-005)	○	○	●	●	○	○	●	○
-0.0684*** (0)	-0.0723*** (0)	-0.0378*** (9.219582e-009)	-0.0364*** (3.141709e-008)	○	○	●	●	○	○	○	●
-0.1260*** (0)	-0.1180*** (0)	-0.0443*** (1.683564e-011)	-0.0333*** (4.161008e-007)	○	○	●	○	●	○	●	○
-0.2267*** (0)	-0.2242*** (0)	-0.1128*** (0)	-0.1135*** (0)	○	○	●	○	●	○	○	●
-0.1109*** (0)	-0.1113*** (0)	-0.0280*** (2.093830e-005)	-0.0277*** (2.582643e-005)	○	○	●	○	○	○	●	●
-0.0512*** (1.376677e-014)	-0.0070 (2.954510e-001)	-0.0391*** (2.923436e-009)	0.0218** (9.157731e-004)	○	○	○	●	●	○	●	○
-0.0506*** (2.919887e-014)	-0.0543*** (3.330669e-016)	-0.0241** (2.470570e-004)	-0.0224** (6.809800e-004)	○	○	○	●	●	○	○	●
0.0015 (8.166330e-001)	-0.0041 (5.350856e-001)	0.0247** (1.774316e-004)	0.0263*** (6.608333e-005)	○	○	○	●	○	○	●	●
-0.1243*** (0)	-0.1243*** (0)	-0.0396*** (1.760661e-009)	-0.0383*** (5.926902e-009)	○	○	○	○	●	○	●	●
-0.0579*** (0)	0.0264*** (7.405917e-005)	-0.0963*** (0)	0.0045 (4.935828e-001)	●	●	●	●	○	○	○	○
-0.1111*** (0)	-0.0428*** (1.317484e-010)	-0.1249*** (0)	-0.0534*** (4.440892e-016)	●	●	●	○	●	○	○	○
-0.0438*** (4.613787e-011)	-0.0248** (1.943511e-004)	-0.0389*** (3.373941e-009)	-0.0160 (1.507347e-002)	●	●	●	○	○	○	●	○
-0.0962*** (0)	-0.0916*** (0)	-0.0902*** (0)	-0.0897*** (0)	●	●	●	○	○	○	○	●
-0.0501*** (5.084821e-014)	0.0417*** (3.865142e-010)	-0.0966*** (0)	0.0128 (5.116910e-002)	●	●	○	●	●	○	○	○
-0.0020 (7.644559e-001)	0.0526*** (2.775558e-015)	-0.0327*** (6.492769e-007)	0.0411*** (4.282505e-010)	●	●	○	●	○	○	●	○
-0.0087 (1.925500e-001)	-0.0112 (9.310595e-002)	-0.0274*** (3.221727e-005)	-0.0251** (1.336290e-004)	●	●	○	●	○	○	○	●
-0.0507*** (2.520206e-014)	-0.0411*** (6.842255e-010)	-0.0370*** (1.794241e-008)	-0.0258*** (8.650398e-005)	●	●	○	○	●	○	●	○
-0.0769*** (0)	-0.0728*** (0)	-0.0759*** (0)	-0.0761*** (0)	●	●	○	○	●	○	○	●
-0.0366*** (3.816736e-008)	-0.0356*** (8.791448e-008)	-0.0252** (1.309078e-004)	-0.0248** (1.666871e-004)	●	●	○	○	○	○	●	●
-0.1321*** (0)	-0.0371*** (2.594221e-008)	-0.1270*** (0)	-0.0103 (1.188994e-001)	●	○	●	●	●	○	○	○
-0.0525*** (3.219647e-015)	0.0034 (6.092972e-001)	-0.0492*** (7.605028e-014)	0.0276*** (2.809494e-005)	●	○	●	●	○	○	●	○
-0.0769*** (0)	-0.0805*** (0)	-0.0502*** (2.420286e-014)	-0.0481*** (2.520206e-013)	●	○	●	●	○	○	○	●

-0.1468*** (0)	-0.1272*** (0)	-0.0743*** (0)	-0.0500*** (2.831069e-014)	●	○	●	○	●	○	●	○
-0.2468*** (0)	-0.2432*** (0)	-0.1447*** (0)	-0.1438*** (0)	●	○	●	○	●	○	○	●
-0.1291*** (0)	-0.1285*** (0)	-0.0553*** (0)	-0.0536*** (3.330669e-016)	●	○	●	○	○	○	●	●
-0.0554*** (1.110223e-016)	-0.0086 (1.961984e-001)	-0.0463*** (1.901257e-012)	0.0191* (3.732921e-003)	●	○	○	●	●	○	●	○
-0.0659*** (0)	-0.0691*** (0)	-0.0443*** (1.707012e-011)	-0.0418*** (2.017474e-010)	●	○	○	●	●	○	○	●
-0.0146 (2.838655e-002)	-0.0198* (2.892377e-003)	0.0025 (7.012729e-001)	0.0048 (4.651617e-001)	●	○	○	●	○	○	●	●
-0.1423*** (0)	-0.1417*** (0)	-0.0625*** (0)	-0.0605*** (0)	●	○	○	○	●	○	●	●
-0.1090*** (0)	-0.0058 (3.798420e-001)	-0.1495*** (0)	-0.0295*** (7.265075e-006)	○	●	●	●	●	○	○	○
-0.0303*** (5.524975e-006)	0.0286*** (1.715569e-005)	-0.0556*** (0)	0.0213* (1.189333e-003)	○	●	●	●	○	○	●	○
-0.0312*** (2.844408e-006)	-0.0338*** (3.929649e-007)	-0.0470*** (9.262591e-013)	-0.0450*** (7.690848e-012)	○	●	●	●	○	○	○	●
-0.0745*** (0)	-0.0558*** (0)	-0.0659*** (0)	-0.0429*** (7.250611e-011)	○	●	●	○	●	○	●	○
-0.1122*** (0)	-0.1076*** (0)	-0.1126*** (0)	-0.1118*** (0)	○	●	●	○	●	○	○	●
-0.0511*** (1.609823e-014)	-0.0497*** (7.982504e-014)	-0.0410*** (4.782735e-010)	-0.0398*** (1.510562e-009)	○	●	●	○	○	○	●	●
-0.0327*** (9.371099e-007)	0.0156 (1.928152e-002)	-0.0506*** (1.432188e-014)	0.0141 (3.219099e-002)	○	●	○	●	●	○	●	○
-0.0158 (1.761202e-002)	-0.0182* (6.286996e-003)	-0.0358*** (5.191920e-008)	-0.0337*** (3.137165e-007)	○	●	○	●	●	○	○	●
0.0247** (2.110441e-004)	0.0199* (2.738859e-003)	0.0146 (2.604698e-002)	0.0165 (1.198881e-002)	○	●	○	●	○	○	●	●
-0.0689*** (0)	-0.0675*** (0)	-0.0536*** (3.330669e-016)	-0.0517*** (3.885781e-015)	○	●	○	○	●	○	●	●
-0.0569*** (0)	-0.0131 (4.940499e-002)	-0.0429*** (7.040857e-011)	0.0174* (8.187597e-003)	○	○	●	●	●	○	●	○
-0.0619*** (0)	-0.0661*** (0)	-0.0325*** (7.985107e-007)	-0.0315*** (1.683493e-006)	○	○	●	●	●	○	○	●
-0.0070 (2.957323e-001)	-0.0130 (5.077567e-002)	0.0184* (5.131697e-003)	0.0195* (3.065617e-003)	○	○	●	●	○	○	●	●
-0.1214*** (0)	-0.1214*** (0)	-0.0364*** (3.172641e-008)	-0.0348*** (1.226583e-007)	○	○	●	○	●	○	●	●
-0.0147 (2.728367e-002)	-0.0201* (2.510527e-003)	0.0118 (7.273101e-002)	0.0150 (2.299722e-002)	○	○	○	●	●	○	●	●
-0.0546*** (2.220446e-016)	0.0306*** (4.455787e-006)	-0.0945*** (0)	0.0073 (2.665450e-001)	●	●	●	●	●	○	○	○
-0.0054 (4.197942e-001)	0.0455*** (8.038792e-012)	-0.0321*** (1.111947e-006)	0.0374*** (1.306553e-008)	●	●	●	●	○	○	●	○
-0.0137 (4.004790e-002)	-0.0168 (1.171594e-002)	-0.0290*** (1.083619e-005)	-0.0277*** (2.582407e-005)	●	●	●	●	○	○	○	●
-0.0499*** (6.872281e-014)	-0.0393*** (3.604959e-009)	-0.0373*** (1.498083e-008)	-0.0246** (1.845356e-004)	●	●	●	○	●	○	●	○
-0.0791*** (0)	-0.0752*** (0)	-0.0765*** (0)	-0.0770*** (0)	●	●	●	○	●	○	○	●
-0.0360*** (6.667414e-008)	-0.0349*** (1.589772e-007)	-0.0248** (1.637674e-004)	-0.0242** (2.294817e-004)	●	●	●	○	○	○	●	●
-0.0112 (9.394030e-002)	0.0317*** (1.908514e-006)	-0.0305*** (3.530134e-006)	0.0290*** (1.020898e-005)	●	●	○	●	●	○	●	○
-0.0040 (5.447945e-001)	-0.0067 (3.151277e-001)	-0.0240** (2.727436e-004)	-0.0220** (8.154682e-004)	●	●	○	●	●	○	○	●
0.0254** (1.402026e-004)	0.0204* (2.143437e-003)	0.0143 (2.978272e-002)	0.0162 (1.357532e-002)	●	●	○	●	○	○	●	●
-0.0474*** (1.049383e-012)	-0.0462*** (3.856027e-012)	-0.0312*** (2.200065e-006)	-0.0298*** (5.947414e-006)	●	●	○	○	●	○	●	●
-0.0578*** (0)	-0.0129 (5.241042e-002)	-0.0462*** (2.257861e-012)	0.0171* (9.314838e-003)	●	○	●	●	●	○	●	○
-0.0709*** (0)	-0.0746*** (0)	-0.0458*** (3.526179e-012)	-0.0441*** (1.975420e-011)	●	○	●	●	●	○	○	●
-0.0178* (7.580746e-003)	-0.0233** (4.575034e-004)	0.0015 (8.181155e-001)	0.0034 (6.095412e-001)	●	○	●	●	○	○	●	●

-0.1378*** (0)	-0.1369*** (0)	-0.0607*** (0)	-0.0580*** (0)	●	○	●	○	●	○	●	●
-0.0270*** (4.881507e-005)	-0.0321*** (1.390179e-006)	-0.0048 (4.628978e-001)	-0.0011 (8.660990e-001)	●	○	○	●	●	○	●	●
-0.0372*** (2.371232e-008)	0.0109 (1.018280e-001)	-0.0533*** (5.551115e-016)	0.0110 (9.429943e-002)	○	●	●	●	●	○	●	○
-0.0251** (1.676972e-004)	-0.0279*** (2.852906e-005)	-0.0419*** (1.866709e-010)	-0.0404*** (8.517188e-010)	○	●	●	●	●	○	○	●
0.0177* (7.818000e-003)	0.0127 (5.696085e-002)	0.0100 (1.295591e-001)	0.0115 (8.159171e-002)	○	●	●	●	○	○	●	●
-0.0624*** (0)	-0.0607*** (0)	-0.0484*** (1.859624e-013)	-0.0460*** (2.560396e-012)	○	●	●	○	●	○	●	●
0.0073 (2.731051e-001)	0.0027 (6.812677e-001)	0.0029 (6.591190e-001)	0.0063 (3.389272e-001)	○	●	○	●	●	○	●	●
-0.0201* (2.556660e-003)	-0.0258** (1.085074e-004)	0.0080 (2.265470e-001)	0.0108 (1.015417e-001)	○	○	●	●	●	○	●	●
-0.0134 (4.464140e-002)	0.0271*** (4.796265e-005)	-0.0301*** (4.878854e-006)	0.0267*** (5.040456e-005)	●	●	●	●	●	○	●	○
-0.0090 (1.776450e-001)	-0.0123 (6.549218e-002)	-0.0255** (1.098214e-004)	-0.0245** (1.941895e-004)	●	●	●	●	●	○	○	●
0.0221** (9.214591e-004)	0.0168 (1.179404e-002)	0.0132 (4.520052e-002)	0.0145 (2.708037e-002)	●	●	●	●	○	○	●	●
-0.0459*** (5.306089e-012)	-0.0446*** (2.193967e-011)	-0.0305*** (3.560564e-006)	-0.0288*** (1.190746e-005)	●	●	●	○	●	○	●	●
0.0116 (8.039500e-002)	0.0069 (3.036488e-001)	0.0067 (3.119647e-001)	0.0100 (1.273105e-001)	●	●	○	●	●	○	●	●
-0.0288*** (1.502021e-005)	-0.0341*** (3.011388e-007)	-0.0054 (4.153690e-001)	-0.0019 (7.744969e-001)	●	○	●	●	●	○	●	●
0.0034 (6.110625e-001)	-0.0014 (8.362762e-001)	0.0004 (9.571652e-001)	0.0035 (5.931885e-001)	○	●	●	●	●	○	●	●
0.0096 (1.511924e-001)	0.0045 (4.985632e-001)	0.0060 (3.643084e-001)	0.0090 (1.732872e-001)	●	●	●	●	●	○	●	●
0.0352*** (1.250451e-007)	0.0466* (1.286619e-003)	0.0322*** (1.016088e-006)	0.0365 (1.709658e-002)	●	●	●	●	●	○	●	●
***FD_x* and *θ_{neu}* (and *P_{neu}*)**											
θ_{neu} (P)	P_{neu} (P)	θ_{neu} (W)	P_{neu} (W)	RR	GC	RD	D_n	D_x	FD_n	FD_x	d_{neu}
-0.3094*** (0)	-0.2161*** (0)	-0.2403*** (0)	-0.1384*** (0)	○	○	○	○	○	○	○	○
-0.3117*** (0)	-0.2149*** (0)	-0.2429*** (0)	-0.1349*** (0)	●	○	○	○	○	○	○	○
-0.2048*** (0)	-0.1089*** (0)	-0.2288*** (0)	-0.1309*** (0)	○	●	○	○	○	○	○	○
-0.3151*** (0)	-0.2228*** (0)	-0.2432*** (0)	-0.1422*** (0)	○	○	●	○	○	○	○	○
-0.1570*** (0)	-0.0688*** (0)	-0.1583*** (0)	-0.0627*** (0)	○	○	○	●	○	○	○	○
-0.2655*** (0)	-0.1622*** (0)	-0.2129*** (0)	-0.0986*** (0)	○	○	○	○	●	○	○	○
-0.0829*** (0)	-0.0382*** (9.288573e-009)	-0.0993*** (0)	-0.0569*** (0)	○	○	○	○	○	●	○	○
-0.2388*** (0)	-0.2342*** (0)	-0.1496*** (0)	-0.1485*** (0)	○	○	○	○	○	○	○	●
-0.1277*** (0)	-0.0509*** (1.976197e-014)	-0.1478*** (0)	-0.0723*** (0)	●	●	○	○	○	○	○	○
-0.3144*** (0)	-0.2205*** (0)	-0.2427*** (0)	-0.1372*** (0)	●	○	●	○	○	○	○	○
-0.1428*** (0)	-0.0582*** (0)	-0.1410*** (0)	-0.0471*** (8.156809e-013)	●	○	○	●	○	○	○	○
-0.2713*** (0)	-0.1632*** (0)	-0.2219*** (0)	-0.0993*** (0)	●	○	○	○	●	○	○	○
-0.0652*** (0)	-0.0270*** (5.144947e-005)	-0.0761*** (0)	-0.0400*** (1.231244e-009)	●	○	○	○	○	●	○	○
-0.2538*** (0)	-0.2486*** (0)	-0.1689*** (0)	-0.1671*** (0)	●	○	○	○	○	○	○	●
-0.2071*** (0)	-0.1109*** (0)	-0.2294*** (0)	-0.1314*** (0)	○	●	●	○	○	○	○	○
-0.1330*** (0)	-0.0429*** (1.103758e-010)	-0.1671*** (0)	-0.0714*** (0)	○	●	○	●	○	○	○	○

-0.1692*** (0)	-0.0628*** (0)	-0.2072*** (0)	-0.0956*** (0)	○	●	○	○	●	○	○	○
-0.0820*** (0)	-0.0369*** (3.044022e-008)	-0.1003*** (0)	-0.0574*** (0)	○	●	○	○	○	●	○	○
-0.1366*** (0)	-0.1305*** (0)	-0.1432*** (0)	-0.1415*** (0)	○	●	○	○	○	○	○	●
-0.1653*** (0)	-0.0786*** (0)	-0.1638*** (0)	-0.0701*** (0)	○	○	●	●	○	○	○	○
-0.2749*** (0)	-0.1734*** (0)	-0.2186*** (0)	-0.1062*** (0)	○	○	●	○	●	○	○	○
-0.0867*** (0)	-0.0424*** (1.830495e-010)	-0.1018*** (0)	-0.0602*** (0)	○	○	●	○	○	●	○	○
-0.2432*** (0)	-0.2388*** (0)	-0.1519*** (0)	-0.1511*** (0)	○	○	●	○	○	○	○	●
-0.1347*** (0)	-0.0360*** (6.556874e-008)	-0.1464*** (0)	-0.0375*** (1.183444e-008)	○	○	○	●	●	○	○	○
-0.0895*** (0)	-0.0600*** (0)	-0.0932*** (0)	-0.0705*** (0)	○	○	○	●	○	●	○	○
-0.0919*** (0)	-0.0905*** (0)	-0.0755*** (0)	-0.0741*** (0)	○	○	○	●	○	○	○	●
-0.0539*** (5.551115e-016)	0.0041 (5.370370e-001)	-0.0861*** (0)	-0.0267*** (5.077298e-005)	○	○	○	○	●	●	○	○
-0.1854*** (0)	-0.1819*** (0)	-0.1078*** (0)	-0.1093*** (0)	○	○	○	○	●	○	○	●
-0.0554*** (1.110223e-016)	-0.0522*** (4.551914e-015)	-0.0640*** (0)	-0.0635*** (0)	○	○	○	○	○	●	○	●
-0.1309*** (0)	-0.0586*** (0)	-0.1467*** (0)	-0.0762*** (0)	●	●	●	○	○	○	○	○
-0.0807*** (0)	-0.0042 (5.257151e-001)	-0.1144*** (0)	-0.0334*** (3.976290e-007)	●	●	○	●	○	○	○	○
-0.1012*** (0)	-0.0119 (7.382681e-002)	-0.1372*** (0)	-0.0448*** (9.949375e-012)	●	●	○	○	●	○	○	○
-0.0543*** (3.330669e-016)	-0.0160 (1.647770e-002)	-0.0721*** (0)	-0.0369*** (2.081281e-008)	●	●	○	○	○	●	○	○
-0.0958*** (0)	-0.0901*** (0)	-0.0997*** (0)	-0.0991*** (0)	●	●	○	○	○	○	○	●
-0.1471*** (0)	-0.0676*** (0)	-0.1399*** (0)	-0.0517*** (3.774758e-015)	●	○	●	●	○	○	○	○
-0.2756*** (0)	-0.1719*** (0)	-0.2214*** (0)	-0.1032*** (0)	●	○	●	○	●	○	○	○
-0.0683*** (0)	-0.0327*** (8.986624e-007)	-0.0758*** (0)	-0.0430*** (6.606937e-011)	●	○	●	○	○	●	○	○
-0.2543*** (0)	-0.2492*** (0)	-0.1689*** (0)	-0.1671*** (0)	●	○	●	○	○	○	○	●
-0.1250*** (0)	-0.0283*** (2.206282e-005)	-0.1359*** (0)	-0.0263*** (6.366883e-005)	●	○	○	●	●	○	○	○
-0.0746*** (0)	-0.0502*** (4.685141e-014)	-0.0727*** (0)	-0.0556*** (0)	●	○	○	●	○	●	○	○
-0.0955*** (0)	-0.0939*** (0)	-0.0787*** (0)	-0.0771*** (0)	●	○	○	●	○	○	○	●
-0.0400*** (1.859620e-009)	0.0130 (5.109251e-002)	-0.0693*** (0)	-0.0136 (3.864496e-002)	●	○	○	○	●	●	○	○
-0.2047*** (0)	-0.2005*** (0)	-0.1348*** (0)	-0.1354*** (0)	●	○	○	○	●	○	○	●
-0.0487*** (2.633449e-013)	-0.0455*** (7.779777e-012)	-0.0536*** (3.330669e-016)	-0.0534*** (4.440892e-016)	●	○	○	○	○	●	○	●
-0.1402*** (0)	-0.0510*** (1.898481e-014)	-0.1711*** (0)	-0.0767*** (0)	○	●	●	●	○	○	○	○
-0.1761*** (0)	-0.0701*** (0)	-0.2108*** (0)	-0.1002*** (0)	○	●	●	○	●	○	○	○
-0.0860*** (0)	-0.0413*** (5.683145e-010)	-0.1027*** (0)	-0.0605*** (0)	○	●	●	○	○	●	○	○
-0.1366*** (0)	-0.1305*** (0)	-0.1427*** (0)	-0.1410*** (0)	○	●	●	○	○	○	○	●
-0.1091*** (0)	-0.0080 (2.281683e-001)	-0.1558*** (0)	-0.0464*** (1.749711e-012)	○	●	○	●	●	○	○	○
-0.0869*** (0)	-0.0566*** (0)	-0.0954*** (0)	-0.0718*** (0)	○	●	○	●	○	●	○	○
-0.0676*** (0)	-0.0651*** (0)	-0.0846*** (0)	-0.0829*** (0)	○	●	○	●	○	○	○	●

-0.0531*** (1.443290e-015)	0.0055 (4.109014e-001)	-0.0870*** (0)	-0.0270*** (3.963711e-005)	○	●	○	○	●	●	○	○
-0.0904*** (0)	-0.0855*** (0)	-0.1061*** (0)	-0.1068*** (0)	○	●	○	○	●	○	○	●
-0.0531*** (1.554312e-015)	-0.0497*** (7.993606e-014)	-0.0650*** (0)	-0.0645*** (0)	○	●	○	○	○	●	○	●
-0.1462*** (0)	-0.0494*** (1.135758e-013)	-0.1542*** (0)	-0.0478*** (3.810285e-013)	○	○	●	●	●	○	○	○
-0.0904*** (0)	-0.0609*** (0)	-0.0937*** (0)	-0.0711*** (0)	○	○	●	●	○	●	○	○
-0.0994*** (0)	-0.0983*** (0)	-0.0812*** (0)	-0.0803*** (0)	○	○	●	●	○	○	○	●
-0.0622*** (0)	-0.0051 (4.406607e-001)	-0.0918*** (0)	-0.0339*** (2.569945e-007)	○	○	●	○	●	●	○	○
-0.1941*** (0)	-0.1911*** (0)	-0.1136*** (0)	-0.1157*** (0)	○	○	●	○	●	○	○	●
-0.0586*** (0)	-0.0555*** (1.110223e-016)	-0.0665*** (0)	-0.0662*** (0)	○	○	●	○	○	●	○	●
-0.0602*** (0)	-0.0206* (1.976350e-003)	-0.0770*** (0)	-0.0420*** (1.792309e-010)	○	○	○	●	●	●	○	○
-0.0593*** (0)	-0.0587*** (0)	-0.0485*** (1.684208e-013)	-0.0493*** (6.561418e-014)	○	○	○	●	●	○	○	●
-0.0721*** (0)	-0.0674*** (0)	-0.0738*** (0)	-0.0737*** (0)	○	○	○	●	○	●	○	●
-0.0150 (2.386892e-002)	-0.0128 (5.419091e-002)	-0.0329*** (5.582352e-007)	-0.0349*** (1.139422e-007)	○	○	○	○	●	●	○	●
-0.0846*** (0)	-0.0137 (3.937408e-002)	-0.1128*** (0)	-0.0383*** (5.926449e-009)	●	●	●	●	○	○	○	○
-0.1053*** (0)	-0.0220** (9.546893e-004)	-0.1357*** (0)	-0.0499*** (3.186340e-014)	●	●	●	○	●	○	○	○
-0.0568*** (0)	-0.0213* (1.346381e-003)	-0.0715*** (0)	-0.0398*** (1.388290e-009)	●	●	●	○	○	●	○	○
-0.0987*** (0)	-0.0933*** (0)	-0.1008*** (0)	-0.1006*** (0)	●	●	●	○	○	○	○	●
-0.0617*** (0)	0.0271*** (4.718233e-005)	-0.1093*** (0)	-0.0124 (5.971643e-002)	●	●	○	●	●	○	○	○
-0.0607*** (0)	-0.0370*** (2.807690e-008)	-0.0674*** (0)	-0.0519*** (2.997602e-015)	●	●	○	●	○	●	○	○
-0.0467*** (2.325584e-012)	-0.0445*** (2.414668e-011)	-0.0624*** (0)	-0.0612*** (0)	●	●	○	●	○	○	○	●
-0.0312*** (2.688674e-006)	0.0223** (8.199936e-004)	-0.0662*** (0)	-0.0113 (8.701946e-002)	●	●	○	○	●	●	○	○
-0.0612*** (0)	-0.0566*** (0)	-0.0752*** (0)	-0.0768*** (0)	●	●	○	○	●	○	○	●
-0.0396*** (2.596076e-009)	-0.0364*** (4.629943e-008)	-0.0505*** (1.676437e-014)	-0.0503*** (2.087219e-014)	●	●	○	○	○	●	○	●
-0.1303*** (0)	-0.0398*** (2.345507e-009)	-0.1345*** (0)	-0.0320*** (1.192233e-006)	●	○	●	●	●	○	○	○
-0.0760*** (0)	-0.0528*** (2.220446e-015)	-0.0726*** (0)	-0.0569*** (0)	●	○	●	●	○	●	○	○
-0.0997*** (0)	-0.0985*** (0)	-0.0801*** (0)	-0.0791*** (0)	●	○	●	●	○	○	○	●
-0.0449*** (1.466272e-011)	0.0039 (5.564449e-001)	-0.0687*** (0)	-0.0183* (5.545503e-003)	●	○	●	○	●	●	○	○
-0.2076*** (0)	-0.2037*** (0)	-0.1356*** (0)	-0.1366*** (0)	●	○	●	○	●	○	○	●
-0.0516*** (8.770762e-015)	-0.0487*** (2.428058e-013)	-0.0547*** (1.110223e-016)	-0.0549*** (1.110223e-016)	●	○	●	○	○	●	○	●
-0.0503*** (3.930190e-014)	-0.0140 (3.580970e-002)	-0.0644*** (0)	-0.0321*** (1.065081e-006)	●	○	○	●	●	●	○	○
-0.0685*** (0)	-0.0676*** (0)	-0.0602*** (0)	-0.0607*** (0)	●	○	○	●	●	○	○	●
-0.0647*** (0)	-0.0601*** (0)	-0.0620*** (0)	-0.0623*** (0)	●	○	○	●	○	●	○	●
-0.0143 (3.173839e-002)	-0.0121 (7.016215e-002)	-0.0316*** (1.597098e-006)	-0.0336*** (3.272229e-007)	●	○	○	○	●	●	○	●
-0.1198*** (0)	-0.0201* (2.558988e-003)	-0.1620*** (0)	-0.0546*** (1.110223e-016)	○	●	●	●	●	○	○	○
-0.0876*** (0)	-0.0573*** (0)	-0.0957*** (0)	-0.0722*** (0)	○	●	●	●	○	●	○	○

-0.0734*** (0)	-0.0712*** (0)	-0.0884*** (0)	-0.0872*** (0)	○	●	●	●	○	○	○	●
-0.0619*** (0)	-0.0043 (5.148277e-001)	-0.0923*** (0)	-0.0341*** (2.191246e-007)	○	●	●	○	●	●	○	○
-0.0957*** (0)	-0.0910*** (0)	-0.1092*** (0)	-0.1103*** (0)	○	●	●	○	●	○	○	●
-0.0563*** (0)	-0.0531*** (1.554312e-015)	-0.0673*** (0)	-0.0670*** (0)	○	●	●	○	○	●	○	●
-0.0571*** (0)	-0.0165 (1.296850e-002)	-0.0794*** (0)	-0.0433*** (4.418710e-011)	○	●	○	●	●	●	○	○
-0.0328*** (8.400349e-007)	-0.0311*** (2.898331e-006)	-0.0579*** (0)	-0.0585*** (0)	○	●	○	●	●	○	○	●
-0.0684*** (0)	-0.0636*** (0)	-0.0755*** (0)	-0.0753*** (0)	○	●	○	●	○	●	○	●
-0.0123 (6.437298e-002)	-0.0100 (1.336099e-001)	-0.0340*** (2.354241e-007)	-0.0359*** (4.756515e-008)	○	●	○	○	●	●	○	●
-0.0653*** (0)	-0.0260*** (9.204076e-005)	-0.0806*** (0)	-0.0462*** (2.189693e-012)	○	○	●	●	●	●	○	○
-0.0704*** (0)	-0.0703*** (0)	-0.0569*** (0)	-0.0585*** (0)	○	○	●	●	●	○	○	●
-0.0727*** (0)	-0.0680*** (0)	-0.0742*** (0)	-0.0742*** (0)	○	○	●	●	○	●	○	●
-0.0230** (5.524521e-004)	-0.0211* (1.571091e-003)	-0.0390*** (3.035934e-009)	-0.0416*** (2.673266e-010)	○	○	●	○	●	●	○	●
-0.0342*** (2.796812e-007)	-0.0302*** (5.872845e-006)	-0.0437*** (3.078049e-011)	-0.0464*** (1.684652e-012)	○	○	○	●	●	●	○	●
-0.0663*** (0)	0.0157 (1.862272e-002)	-0.1073*** (0)	-0.0183* (5.493927e-003)	●	●	●	●	●	○	○	○
-0.0619*** (0)	-0.0396*** (2.830382e-009)	-0.0671*** (0)	-0.0533*** (5.551115e-016)	●	●	●	●	○	●	○	○
-0.0508*** (2.242651e-014)	-0.0491*** (1.666445e-013)	-0.0638*** (0)	-0.0635*** (0)	●	●	●	●	○	○	○	●
-0.0350*** (1.513315e-007)	0.0141 (3.472730e-002)	-0.0652*** (0)	-0.0157 (1.683545e-002)	●	●	●	○	●	●	○	○
-0.0656*** (0)	-0.0615*** (0)	-0.0767*** (0)	-0.0791*** (0)	●	●	●	○	●	○	○	●
-0.0422*** (2.338190e-010)	-0.0392*** (3.946560e-009)	-0.0515*** (5.107026e-015)	-0.0518*** (3.552714e-015)	●	●	●	○	○	●	○	●
-0.0377*** (1.466750e-008)	-0.0017 (7.998632e-001)	-0.0597*** (0)	-0.0288*** (1.168265e-005)	●	●	○	●	●	●	○	○
-0.0198* (2.925385e-003)	-0.0183* (5.997323e-003)	-0.0442*** (1.801859e-011)	-0.0452*** (6.563416e-012)	●	●	○	●	●	○	○	●
-0.0524*** (3.441691e-015)	-0.0476*** (8.331114e-013)	-0.0579*** (0)	-0.0582*** (0)	●	●	○	●	○	●	○	●
-0.0082 (2.186980e-001)	-0.0058 (3.800061e-001)	-0.0295*** (7.171957e-006)	-0.0316*** (1.558101e-006)	●	●	○	○	●	●	○	●
-0.0533*** (1.110223e-015)	-0.0193* (3.678468e-003)	-0.0641*** (0)	-0.0348*** (1.265356e-007)	●	○	●	●	●	●	○	○
-0.0741*** (0)	-0.0738*** (0)	-0.0620*** (0)	-0.0634*** (0)	●	○	●	●	●	○	○	●
-0.0660*** (0)	-0.0616*** (0)	-0.0625*** (0)	-0.0630*** (0)	●	○	●	●	○	●	○	●
-0.0194* (3.546776e-003)	-0.0176* (8.174852e-003)	-0.0333*** (4.056248e-007)	-0.0362*** (3.906919e-008)	●	○	●	○	●	●	○	●
-0.0329*** (7.544105e-007)	-0.0289*** (1.457828e-005)	-0.0412*** (3.941120e-010)	-0.0440*** (2.234191e-011)	●	○	○	●	●	●	○	●
-0.0623*** (0)	-0.0221** (9.096329e-004)	-0.0826*** (0)	-0.0473*** (6.686873e-013)	○	●	●	●	●	●	○	○
-0.0426*** (1.606367e-010)	-0.0413*** (5.419917e-010)	-0.0644*** (0)	-0.0657*** (0)	○	●	●	●	●	○	○	●
-0.0688*** (0)	-0.0639*** (0)	-0.0757*** (0)	-0.0756*** (0)	○	●	●	●	○	●	○	●
-0.0206* (1.936646e-003)	-0.0186* (5.216549e-003)	-0.0398*** (1.445563e-009)	-0.0423*** (1.276734e-010)	○	●	●	○	●	●	○	●
-0.0297*** (8.465647e-006)	-0.0254** (1.340031e-004)	-0.0456*** (4.045764e-012)	-0.0483*** (2.120526e-013)	○	●	○	●	●	●	○	●
-0.0391*** (4.175287e-009)	-0.0353*** (1.165826e-007)	-0.0474*** (5.776490e-013)	-0.0505*** (1.609823e-014)	○	○	●	●	●	●	○	●
-0.0400*** (1.813445e-009)	-0.0067 (3.158078e-001)	-0.0591*** (0)	-0.0315*** (1.700016e-006)	●	●	●	●	●	●	○	○

-0.0251** (1.610184e-004)	-0.0243** (2.693022e-004)	-0.0459*** (2.938316e-012)	-0.0480*** (3.063105e-013)	●	●	●	●	●	○	○	●
-0.0537*** (7.771561e-016)	-0.0491*** (1.678657e-013)	-0.0584*** (0)	-0.0590*** (0)	●	●	●	●	○	●	○	●
-0.0124 (6.346420e-002)	-0.0105 (1.158492e-001)	-0.0310*** (2.425026e-006)	-0.0339*** (2.531162e-007)	●	●	●	○	●	●	○	●
-0.0226** (6.799651e-004)	-0.0184* (5.837299e-003)	-0.0378*** (9.469923e-009)	-0.0407*** (6.155787e-010)	●	●	○	●	●	●	○	●
-0.0361*** (5.772076e-008)	-0.0324*** (1.170538e-006)	-0.0422*** (1.395616e-010)	-0.0456*** (4.255596e-012)	●	○	●	●	●	●	○	●
-0.0346*** (2.045020e-007)	-0.0305*** (4.478624e-006)	-0.0490*** (9.725554e-014)	-0.0520*** (2.664535e-015)	○	●	●	●	●	●	○	●
-0.0253** (1.415664e-004)	-0.0214* (1.303405e-003)	-0.0387*** (4.067748e-009)	-0.0422*** (1.421798e-010)	●	●	●	●	●	●	○	●
-0.0539*** (5.617120e-016)	-0.0157*** (9.168664e-007)	-0.0675*** (1.005795e-024)	-0.0439*** (1.049893e-017)	●	●	●	●	●	●	○	●

*** $P<0.0001$; ** $0.0001\le P<0.001$; * $0.001\le P<0.01$, Spearman test
