# Supplementary material for: Pervasive Hitchhiking at Coding and Regulatory Sites in Humans
Source: PLoS Genet. 2009 Jan 16;5(1):e1000336. doi: 10.1371/journal.pgen.1000336 (PMC2613029; doi:10.1371/journal.pgen.1000336)
Supplement: Table S4 — Partial Spearman rank correlation coefficients controlling for the depth of sequencing coverage (SC) of Watson data. Partial Spearman rank correlation coefficients between functional divergence [i.e. the divergence at coding sites (Dn) or the divergence at conserved noncoding region (Dx)] and neutral polymorphism [i.e.,the level of neutral polymorphism (θneu) and the level of normalized neutral polymorphism (Pneu = θneu/dneu)], and between functional constraints [i.e.,the number of codons (FDn) and the number of conserved noncoding sites (FDx)] and neutral polymorphism (θneu and Pneu) are given. Spearman's partial correlation coefficients were calculated by controlling for all possible combinations of potentially confounding variables. The results of representative combinations are given here. Closed circles (•) indicate the controlled variables. These variables are GC content (GC), the density of simple repeats (RD), the depth of sequencing coverage (SC), the divergence at coding sites (Dn), the divergence at conserved noncoding region (Dx), the number of codons (FDn), the number of conserved noncoding sites (FDx), and the level of neutral divergence rate (dneu). Open circles (○) indicate the variables that were not controlled in a particular analysis. (W) indicates the results are based on the Watson data. P-values are given in parentheses. (0.2 MB PDF) [file pgen.1000336.s014.pdf]

Table S4. Partial Spearman rank correlation coefficients controlling for the depth of sequencing coverage (SC) of Watson data. Partial Spearman rank correlation coefficients between functional divergence [i.e. the divergence at coding sites (D_n) or the divergence at conserved noncoding region (D_x)] and neutral polymorphism [i.e. the level of neutral polymorphism (θ_{neu}) and the level of normalized neutral polymorphism ($P_{neu}=\theta_{neu}/d_{neu}$)], and between functional constraints [i.e. the number of codons (FD_n) and the number of conserved noncoding sites (FD_x)] and neutral polymorphism (θ_{neu} and P_{neu}) are given. Spearman's partial correlation coefficients were calculated by controlling for all possible combinations of potentially confounding variables. The results of representative combinations are given here. Closed circles (●) indicate the controlled variables. These variables are GC content (GC), the density of simple repeats (RD), the depth of sequencing coverage (SC), the divergence at coding sites (D_n), the divergence at conserved noncoding region (D_x), the number of codons (FD_n), the number of conserved noncoding sites (FD_x), and the level of neutral divergence rate (d_{neu}). Open circles (○) indicate the variables that were not controlled in a particular analysis. (W) indicates the results are based on the Watson data. *P*-values are given in parentheses.

**D_n and θ_{neu}**	**D_n and P_{neu}**									
Watson θ_{neu}	Watson P_{neu}	RR	GC	RD	SC	D_n	D_x	FD_n	FD_x	d_{neu}
-0.1484*** (0)	-0.0835*** (0)	○	○	○	●	○	○	○	○	○
-0.1842*** (0)	-0.1030*** (0)	●	○	○	●	○	○	○	○	○
-0.1488*** (0)	-0.1021*** (0)	○	●	○	●	○	○	○	○	○
-0.1591*** (0)	-0.0987*** (0)	○	○	●	●	○	○	○	○	○
-0.1335*** (0)	-0.0693*** (0)	○	○	○	●	○	●	○	○	○
0.0420*** (1.794467e-010)	-0.0316*** (1.555604e-006)	○	○	○	●	○	○	●	○	○
-0.0224** (6.663786e-004)	-0.0324*** (8.417918e-007)	○	○	○	●	○	○	○	●	○
-0.0883*** (0)	-0.0868*** (0)	○	○	○	●	○	○	○	○	●
-0.1041*** (0)	-0.0731*** (0)	●	●	○	●	○	○	○	○	○
-0.1741*** (0)	-0.1046*** (0)	●	○	●	●	○	○	○	○	○
-0.1712*** (0)	-0.0902*** (0)	●	○	○	●	○	●	○	○	○
0.0219** (8.594909e-004)	-0.0461*** (2.316258e-012)	●	○	○	●	○	○	●	○	○
-0.0598*** (0)	-0.0556*** (0)	●	○	○	●	○	○	○	●	○
-0.1260*** (0)	-0.1231*** (0)	●	○	○	●	○	○	○	○	●
-0.1532*** (0)	-0.1070*** (0)	○	●	●	●	○	○	○	○	○
-0.1351*** (0)	-0.0889*** (0)	○	●	○	●	○	●	○	○	○
0.0406*** (6.481587e-010)	-0.0329*** (5.700460e-007)	○	●	○	●	○	○	●	○	○
-0.0411*** (4.035233e-010)	-0.0520*** (2.664535e-015)	○	●	○	●	○	○	○	●	○
-0.1057*** (0)	-0.1043*** (0)	○	●	○	●	○	○	○	○	●
-0.1447*** (0)	-0.0848*** (0)	○	○	●	●	○	●	○	○	○
0.0422*** (1.358813e-010)	-0.0316*** (1.595835e-006)	○	○	●	●	○	○	●	○	○

-0.0320*** (1.120025e-006)	-0.0413*** (3.298196e-010)	○	○	●	●	○	○	○	●	○
-0.1013*** (0)	-0.1018*** (0)	○	○	●	●	○	○	○	○	●
0.0448*** (9.282686e-012)	-0.0282*** (1.813066e-005)	○	○	○	●	○	●	●	○	○
-0.0217** (9.544143e-004)	-0.0355*** (6.782377e-008)	○	○	○	●	○	●	○	●	○
-0.0731*** (0)	-0.0728*** (0)	○	○	○	●	○	●	○	○	●
0.0176* (7.539606e-003)	-0.0467*** (1.267209e-012)	○	○	○	●	○	○	●	●	○
-0.0237** (3.102589e-004)	-0.0251** (1.360732e-004)	○	○	○	●	○	○	●	○	●
-0.0316*** (1.612537e-006)	-0.0315*** (1.731546e-006)	○	○	○	●	○	○	○	●	●
-0.1039*** (0)	-0.0758*** (0)	●	●	●	●	○	○	○	○	○
-0.0941*** (0)	-0.0624*** (0)	●	●	○	●	○	●	○	○	○
0.0207* (1.635053e-003)	-0.0467*** (1.259437e-012)	●	●	○	●	○	○	●	○	○
-0.0310*** (2.387944e-006)	-0.0458*** (3.362199e-012)	●	●	○	●	○	○	○	●	○
-0.0841*** (0)	-0.0834*** (0)	●	●	○	●	○	○	○	○	●
-0.1627*** (0)	-0.0926*** (0)	●	○	●	●	○	●	○	○	○
0.0219** (8.876913e-004)	-0.0457*** (3.550049e-012)	●	○	●	●	○	○	●	○	○
-0.0596*** (0)	-0.0577*** (0)	●	○	●	●	○	○	○	●	○
-0.1232*** (0)	-0.1226*** (0)	●	○	●	●	○	○	○	○	●
0.0241** (2.515330e-004)	-0.0431*** (5.654122e-011)	●	○	○	●	○	●	●	○	○
-0.0584*** (0)	-0.0582*** (0)	●	○	○	●	○	●	○	●	○
-0.1121*** (0)	-0.1104*** (0)	●	○	○	●	○	●	○	○	●
0.0028 (6.677852e-001)	-0.0574*** (0)	●	○	○	●	○	○	●	●	○
-0.0243** (2.207753e-004)	-0.0257*** (9.535484e-005)	●	○	○	●	○	○	●	○	●
-0.0585*** (0)	-0.0575*** (0)	●	○	○	●	○	○	○	●	●
-0.1393*** (0)	-0.0935*** (0)	○	●	●	●	○	●	○	○	○
0.0412*** (3.878577e-010)	-0.0326*** (7.491163e-007)	○	●	●	●	○	○	●	○	○
-0.0433*** (4.891265e-011)	-0.0539*** (2.220446e-016)	○	●	●	●	○	○	○	●	○
-0.1098*** (0)	-0.1093*** (0)	○	●	●	●	○	○	○	○	●
0.0434*** (4.267864e-011)	-0.0296*** (6.798181e-006)	○	●	○	●	○	●	●	○	○
-0.0404*** (8.136856e-010)	-0.0534*** (4.440892e-016)	○	●	○	●	○	●	○	●	○
-0.0914*** (0)	-0.0911*** (0)	○	●	○	●	○	●	○	○	●
0.0162 (1.395493e-002)	-0.0480*** (2.835510e-013)	○	●	○	●	○	○	●	●	○
-0.0245** (1.929563e-004)	-0.0259*** (8.290254e-005)	○	●	○	●	○	○	●	○	●
-0.0513*** (6.439294e-015)	-0.0511*** (8.104628e-015)	○	●	○	●	○	○	○	●	●

0.0452*** (6.072365e-012)	-0.0281*** (1.989336e-005)	○	○	●	●	○	●	●	○	○
-0.0312*** (2.157583e-006)	-0.0441*** (2.076084e-011)	○	○	●	●	○	●	○	●	○
-0.0865*** (0)	-0.0881*** (0)	○	○	●	●	○	●	○	○	●
0.0163 (1.301204e-002)	-0.0480*** (3.020917e-013)	○	○	●	●	○	○	●	●	○
-0.0229** (4.915017e-004)	-0.0242** (2.316099e-004)	○	○	●	●	○	○	●	○	●
-0.0398*** (1.487575e-009)	-0.0406*** (6.894404e-010)	○	○	●	●	○	○	○	●	●
0.0183* (5.449267e-003)	-0.0396*** (1.816519e-009)	○	○	○	●	○	●	●	●	○
-0.0205* (1.873100e-003)	-0.0221** (7.765351e-004)	○	○	○	●	○	●	●	○	●
-0.0348*** (1.233967e-007)	-0.0343*** (1.936200e-007)	○	○	○	●	○	●	○	●	●
-0.0388*** (3.642590e-009)	-0.0400*** (1.157386e-009)	○	○	○	●	○	○	●	●	●
-0.0942*** (0)	-0.0652*** (0)	●	●	●	●	○	●	○	○	○
0.0210* (1.452075e-003)	-0.0462*** (2.085221e-012)	●	●	●	●	○	○	●	○	○
-0.0317*** (1.494569e-006)	-0.0469*** (9.888756e-013)	●	●	●	●	○	○	○	●	○
-0.0851*** (0)	-0.0855*** (0)	●	●	●	●	○	○	○	○	●
0.0230** (4.713263e-004)	-0.0436*** (3.317846e-011)	●	●	○	●	○	●	●	○	○
-0.0308*** (2.943367e-006)	-0.0475*** (5.141443e-013)	●	●	○	●	○	●	○	●	○
-0.0726*** (0)	-0.0730*** (0)	●	●	○	●	○	●	○	○	●
0.0025 (7.047857e-001)	-0.0575*** (0)	●	●	○	●	○	○	●	●	○
-0.0236** (3.362312e-004)	-0.0250** (1.443408e-004)	●	●	○	●	○	○	●	○	●
-0.0412*** (3.739248e-010)	-0.0414*** (3.283945e-010)	●	●	○	●	○	○	○	●	●
0.0241** (2.576414e-004)	-0.0426*** (9.156476e-011)	●	○	●	●	○	●	●	○	○
-0.0582*** (0)	-0.0601*** (0)	●	○	●	●	○	●	○	●	○
-0.1104*** (0)	-0.1108*** (0)	●	○	●	●	○	●	○	○	●
0.0028 (6.686183e-001)	-0.0574*** (0)	●	○	●	●	○	○	●	●	○
-0.0242** (2.316516e-004)	-0.0255** (1.087283e-004)	●	○	●	●	○	○	●	○	●
-0.0592*** (0)	-0.0591*** (0)	●	○	●	●	○	○	○	●	●
0.0025 (6.999443e-001)	-0.0507*** (1.265654e-014)	●	○	○	●	○	●	●	●	○
-0.0217** (9.562586e-004)	-0.0234** (3.820223e-004)	●	○	○	●	○	●	●	○	●
-0.0600*** (0)	-0.0587*** (0)	●	○	○	●	○	●	○	●	●
-0.0375*** (1.163820e-008)	-0.0388*** (3.690394e-009)	●	○	○	●	○	○	●	●	●
0.0441*** (2.105505e-011)	-0.0291*** (9.596221e-006)	○	●	●	●	○	●	●	○	○
-0.0425*** (1.028878e-010)	-0.0553*** (0)	○	●	●	●	○	●	○	●	○
-0.0955*** (0)	-0.0959*** (0)	○	●	●	●	○	●	○	○	●

0.0155 (1.842674e-002)	-0.0488*** (1.253442e-013)	○	●	●	●	○	○	●	●	○
-0.0237** (3.103718e-004)	-0.0250** (1.469243e-004)	○	●	●	●	○	○	●	○	●
-0.0529*** (8.881784e-016)	-0.0530*** (7.771561e-016)	○	●	●	●	○	○	○	●	●
0.0161 (1.430726e-002)	-0.0415*** (2.838294e-010)	○	●	○	●	○	●	●	●	○
-0.0214* (1.161412e-003)	-0.0230** (4.669270e-004)	○	●	○	●	○	●	●	○	●
-0.0528*** (9.992007e-016)	-0.0523*** (1.776357e-015)	○	●	○	●	○	●	○	●	●
-0.0397*** (1.544897e-009)	-0.0410*** (4.819685e-010)	○	●	○	●	○	○	●	●	●
0.0169 (1.039759e-002)	-0.0410*** (4.664537e-010)	○	○	●	●	○	●	●	●	○
-0.0196* (2.936739e-003)	-0.0211* (1.316466e-003)	○	○	●	●	○	●	●	○	●
-0.0426*** (9.595047e-011)	-0.0429*** (6.723444e-011)	○	○	●	●	○	●	○	●	●
-0.0392*** (2.503486e-009)	-0.0405*** (7.427322e-010)	○	○	●	●	○	○	●	●	●
-0.0318*** (1.346045e-006)	-0.0341*** (2.125840e-007)	○	○	○	●	○	●	●	●	●
0.0233** (3.997541e-004)	-0.0431*** (5.920742e-011)	●	●	●	●	○	●	●	○	○
-0.0314*** (1.855196e-006)	-0.0486*** (1.477707e-013)	●	●	●	●	○	●	○	●	○
-0.0739*** (0)	-0.0752*** (0)	●	●	●	●	○	●	○	○	●
0.0024 (7.109458e-001)	-0.0577*** (0)	●	●	●	●	○	○	●	●	○
-0.0234** (3.875706e-004)	-0.0246** (1.829806e-004)	●	●	●	●	○	○	●	○	●
-0.0419*** (1.822558e-010)	-0.0424*** (1.109141e-010)	●	●	●	●	○	○	○	●	●
0.0031 (6.411357e-001)	-0.0505*** (1.565414e-014)	●	●	○	●	○	●	●	●	○
-0.0210* (1.442739e-003)	-0.0227** (5.760952e-004)	●	●	○	●	○	●	●	○	●
-0.0426*** (9.957624e-011)	-0.0424*** (1.146694e-010)	●	●	○	●	○	●	○	●	●
-0.0365*** (2.943440e-008)	-0.0378*** (9.060346e-009)	●	●	○	●	○	○	●	●	●
0.0025 (7.013048e-001)	-0.0508*** (1.121325e-014)	●	○	●	●	○	●	●	●	○
-0.0216* (1.019735e-003)	-0.0231** (4.450541e-004)	●	○	●	●	○	●	●	○	●
-0.0607*** (0)	-0.0602*** (0)	●	○	●	●	○	●	○	●	●
-0.0376*** (1.086180e-008)	-0.0390*** (3.194188e-009)	●	○	●	●	○	○	●	●	●
-0.0327*** (6.993112e-007)	-0.0350*** (1.050080e-007)	●	○	○	●	○	●	●	●	●
0.0155 (1.856714e-002)	-0.0422*** (1.462791e-010)	○	●	●	●	○	●	●	●	○
-0.0205* (1.887505e-003)	-0.0220** (8.427144e-004)	○	●	●	●	○	●	●	○	●
-0.0544*** (1.110223e-016)	-0.0542*** (2.220446e-016)	○	●	●	●	○	●	○	●	●
-0.0399*** (1.385339e-009)	-0.0411*** (4.182614e-010)	○	●	●	●	○	○	●	●	●
-0.0333*** (4.029085e-007)	-0.0357*** (5.892904e-008)	○	●	○	●	○	●	●	●	●
-0.0324*** (8.405642e-007)	-0.0348*** (1.198690e-007)	○	○	●	●	○	●	●	●	●

0.0030 (6.465238e-001)	-0.0506*** (1.387779e-014)	●	●	●	●	○	●	●	●	○
-0.0207* (1.699385e-003)	-0.0222** (7.508353e-004)	●	●	●	●	○	●	●	○	●
-0.0433*** (4.802625e-011)	-0.0435*** (3.823164e-011)	●	●	●	●	○	●	○	●	●
-0.0366*** (2.731661e-008)	-0.0380*** (8.033108e-009)	●	●	●	●	○	○	●	●	●
-0.0312*** (2.086485e-006)	-0.0337*** (3.125535e-007)	●	●	○	●	○	●	●	●	●
-0.0327*** (6.550178e-007)	-0.0352*** (9.115795e-008)	●	○	●	●	○	●	●	●	●
-0.0335*** (3.620750e-007)	-0.0358*** (5.109727e-008)	○	●	●	●	○	●	●	●	●
-0.0313*** (1.967982e-006)	-0.0338*** (2.834373e-007)	●	●	●	●	○	●	●	●	●
-0.0648*** (6.694256e-023)	-0.0269*** (4.436343e-005)	●	●	●	●	○	●	●	●	●
**D_x and θ_{neu}**	**D_x and P_{neu}**									
Watson θ_{neu}	Watson P_{neu}	RR	GC	RD	SC	D_n	D_x	FD_n	FD_x	d_{neu}
-0.0861*** (0)	-0.0741*** (0)	○	○	○	●	○	○	○	○	○
-0.0805*** (0)	-0.0693*** (0)	●	○	○	●	○	○	○	○	○
-0.0836*** (0)	-0.0756*** (0)	○	●	○	●	○	○	○	○	○
-0.0883*** (0)	-0.0784*** (0)	○	○	●	●	○	○	○	○	○
-0.0560*** (0)	-0.0577*** (0)	○	○	○	●	●	○	○	○	○
-0.0464*** (1.670775e-012)	-0.0589*** (0)	○	○	○	●	○	○	●	○	○
0.0089 (1.745154e-001)	-0.0364*** (3.129826e-008)	○	○	○	●	○	○	○	●	○
-0.0802*** (0)	-0.0745*** (0)	○	○	○	●	○	○	○	○	●
-0.0617*** (0)	-0.0615*** (0)	●	●	○	●	○	○	○	○	○
-0.0749*** (0)	-0.0691*** (0)	●	○	●	●	○	○	○	○	○
-0.0418*** (2.128405e-010)	-0.0482*** (2.469136e-013)	●	○	○	●	●	○	○	○	○
-0.0335*** (3.547692e-007)	-0.0507*** (1.321165e-014)	●	○	○	●	○	○	●	○	○
0.0210* (1.451977e-003)	-0.0301*** (4.904770e-006)	●	○	○	●	○	○	○	●	○
-0.0771*** (0)	-0.0713*** (0)	●	○	○	●	○	○	○	○	●
-0.0860*** (0)	-0.0783*** (0)	○	●	●	●	○	○	○	○	○
-0.0551*** (0)	-0.0563*** (0)	○	●	○	●	●	○	○	○	○
-0.0434*** (4.048162e-011)	-0.0564*** (0)	○	●	○	●	○	○	●	○	○
0.0166 (1.143016e-002)	-0.0290*** (1.045216e-005)	○	●	○	●	○	○	○	●	○
-0.0814*** (0)	-0.0757*** (0)	○	●	○	●	○	○	○	○	●
-0.0579*** (0)	-0.0599*** (0)	○	○	●	●	●	○	○	○	○
-0.0483*** (2.020606e-013)	-0.0606*** (0)	○	○	●	●	○	○	●	○	○
0.0124 (5.964048e-002)	-0.0334*** (3.807884e-007)	○	○	●	●	○	○	○	●	○

-0.0837*** (0)	-0.0787*** (0)	○	○	●	●	○	○	○	○	●
-0.0491*** (8.737455e-014)	-0.0571*** (0)	○	○	○	●	●	○	●	○	○
0.0071 (2.778932e-001)	-0.0392*** (2.562068e-009)	○	○	○	●	●	○	○	●	○
-0.0630*** (0)	-0.0575*** (0)	○	○	○	●	●	○	○	○	●
-0.0015 (8.140994e-001)	-0.0390*** (3.047346e-009)	○	○	○	●	○	○	●	●	○
-0.0629*** (0)	-0.0576*** (0)	○	○	○	●	○	○	●	○	●
-0.0363*** (3.504312e-008)	-0.0311*** (2.350417e-006)	○	○	○	●	○	○	○	●	●
-0.0613*** (0)	-0.0629*** (0)	●	●	●	●	○	○	○	○	○
-0.0425*** (1.098766e-010)	-0.0483*** (2.143841e-013)	●	●	○	●	●	○	○	○	○
-0.0357*** (5.751271e-008)	-0.0515*** (4.662937e-015)	●	●	○	●	○	○	●	○	○
0.0069 (2.955515e-001)	-0.0358*** (5.160333e-008)	●	●	○	●	○	○	○	●	○
-0.0663*** (0)	-0.0609*** (0)	●	●	○	●	○	○	○	○	●
-0.0408*** (5.444519e-010)	-0.0490*** (8.959500e-014)	●	○	●	●	●	○	○	○	○
-0.0334*** (3.816097e-007)	-0.0514*** (5.329071e-015)	●	○	●	●	○	○	●	○	○
0.0208* (1.568755e-003)	-0.0294*** (7.993746e-006)	●	○	●	●	○	○	○	●	○
-0.0751*** (0)	-0.0702*** (0)	●	○	●	●	○	○	○	○	●
-0.0350*** (1.084014e-007)	-0.0479*** (3.180789e-013)	●	○	○	●	●	○	●	○	○
0.0164 (1.290537e-002)	-0.0346*** (1.496938e-007)	●	○	○	●	●	○	○	●	○
-0.0511*** (7.993606e-015)	-0.0457*** (3.679723e-012)	●	○	○	●	●	○	○	○	●
0.0017 (8.002987e-001)	-0.0378*** (8.956846e-009)	●	○	○	●	○	○	●	●	○
-0.0495*** (5.506706e-014)	-0.0444*** (1.548295e-011)	●	○	○	●	○	○	●	○	●
-0.0175* (7.727445e-003)	-0.0128 (5.272801e-002)	●	○	○	●	○	○	○	●	●
-0.0573*** (0)	-0.0585*** (0)	○	●	●	●	●	○	○	○	○
-0.0457*** (3.817391e-012)	-0.0584*** (0)	○	●	●	●	○	○	●	○	○
0.0169 (1.031112e-002)	-0.0289*** (1.136953e-005)	○	●	●	●	○	○	○	●	○
-0.0837*** (0)	-0.0786*** (0)	○	●	●	●	○	○	○	○	●
-0.0460*** (2.684741e-012)	-0.0545*** (1.110223e-016)	○	●	○	●	●	○	●	○	○
0.0148 (2.491327e-002)	-0.0315*** (1.694385e-006)	○	●	○	●	●	○	○	●	○
-0.0617*** (0)	-0.0561*** (0)	○	●	○	●	●	○	○	○	●
0.0020 (7.654199e-001)	-0.0362*** (3.868845e-008)	○	●	○	●	○	○	●	●	○
-0.0602*** (0)	-0.0549*** (1.110223e-016)	○	●	○	●	○	○	●	○	●
-0.0287*** (1.291443e-005)	-0.0235** (3.626169e-004)	○	●	○	●	○	○	○	●	●
-0.0510*** (9.214851e-015)	-0.0589*** (0)	○	○	●	●	●	○	●	○	○

0.0100 (1.304082e-001)	-0.0368*** (2.330053e-008)	○	○	●	●	●	○	○	●	○
-0.0650*** (0)	-0.0597*** (0)	○	○	●	●	●	○	○	○	●
-0.0008 (9.052153e-001)	-0.0385*** (5.048841e-009)	○	○	●	●	○	○	●	●	○
-0.0643*** (0)	-0.0592*** (0)	○	○	●	●	○	○	●	○	●
-0.0330*** (5.290512e-007)	-0.0274*** (3.123140e-005)	○	○	●	●	○	○	○	●	●
-0.0053 (4.237589e-001)	-0.0301*** (4.749013e-006)	○	○	○	●	●	○	●	●	○
-0.0617*** (0)	-0.0563*** (0)	○	○	○	●	●	○	●	○	●
-0.0391*** (2.735052e-009)	-0.0339*** (2.623484e-007)	○	○	○	●	●	○	○	●	●
-0.0400*** (1.166316e-009)	-0.0344*** (1.716805e-007)	○	○	○	●	○	○	●	●	●
-0.0427*** (8.853884e-011)	-0.0496*** (4.729550e-014)	●	●	●	●	●	○	○	○	○
-0.0362*** (3.682690e-008)	-0.0527*** (1.221245e-015)	●	●	●	●	○	○	●	○	○
0.0070 (2.860584e-001)	-0.0356*** (6.255422e-008)	●	●	●	●	○	○	○	●	○
-0.0668*** (0)	-0.0620*** (0)	●	●	●	●	○	○	○	○	●
-0.0371*** (1.752971e-008)	-0.0488*** (1.212364e-013)	●	●	○	●	●	○	●	○	○
0.0055 (4.049192e-001)	-0.0380*** (7.731233e-009)	●	●	○	●	●	○	○	●	○
-0.0509*** (9.547918e-015)	-0.0456*** (4.356404e-012)	●	●	○	●	●	○	○	○	●
-0.0025 (7.071457e-001)	-0.0395*** (1.869480e-009)	●	●	○	●	○	○	●	●	○
-0.0504*** (1.909584e-014)	-0.0452*** (6.437184e-012)	●	●	○	●	○	○	●	○	●
-0.0249** (1.553185e-004)	-0.0196* (2.862099e-003)	●	●	○	●	○	○	○	●	●
-0.0349*** (1.156878e-007)	-0.0487*** (1.350031e-013)	●	○	●	●	●	○	●	○	○
0.0164 (1.284129e-002)	-0.0339*** (2.582221e-007)	●	○	●	●	●	○	○	●	○
-0.0511*** (7.549517e-015)	-0.0463*** (2.049028e-012)	●	○	●	●	●	○	○	○	●
0.0017 (7.978613e-001)	-0.0377*** (1.016011e-008)	●	○	●	●	○	○	●	●	○
-0.0497*** (4.041212e-014)	-0.0450*** (7.844392e-012)	●	○	●	●	○	○	●	○	●
-0.0174* (8.287566e-003)	-0.0123 (6.132110e-002)	●	○	●	●	○	○	○	●	●
0.0011 (8.663580e-001)	-0.0266*** (5.158055e-005)	●	○	○	●	●	○	●	●	○
-0.0483*** (2.217115e-013)	-0.0431*** (5.872880e-011)	●	○	○	●	●	○	●	○	●
-0.0221** (7.672076e-004)	-0.0173* (8.737930e-003)	●	○	○	●	●	○	○	●	●
-0.0289*** (1.115662e-005)	-0.0235** (3.661907e-004)	●	○	○	●	○	○	●	●	●
-0.0483*** (2.096101e-013)	-0.0566*** (0)	○	●	●	●	●	○	●	○	○
0.0149 (2.354959e-002)	-0.0315*** (1.736331e-006)	○	●	●	●	●	○	○	●	○
-0.0636*** (0)	-0.0584*** (0)	○	●	●	●	●	○	○	○	●
0.0017 (7.959467e-001)	-0.0365*** (2.974876e-008)	○	●	●	●	○	○	●	●	○

-0.0620*** (0)	-0.0569*** (0)	○	●	●	●	○	○	●	○	●
-0.0282*** (1.821035e-005)	-0.0229** (5.046901e-004)	○	●	●	●	○	○	○	●	●
-0.0014 (8.314947e-001)	-0.0269*** (4.426487e-005)	○	●	○	●	●	○	●	●	○
-0.0590*** (0)	-0.0536*** (3.330669e-016)	○	●	○	●	●	○	●	○	●
-0.0313*** (1.908092e-006)	-0.0261*** (7.380151e-005)	○	●	○	●	●	○	○	●	●
-0.0368*** (2.323183e-008)	-0.0311*** (2.233707e-006)	○	●	○	●	○	○	●	●	●
-0.0042 (5.207283e-001)	-0.0293*** (8.645598e-006)	○	○	●	●	●	○	●	●	○
-0.0632*** (0)	-0.0580*** (0)	○	○	●	●	●	○	●	○	●
-0.0363*** (3.326436e-008)	-0.0308*** (2.887685e-006)	○	○	●	●	●	○	○	●	●
-0.0390*** (3.102354e-009)	-0.0332*** (4.396624e-007)	○	○	●	●	○	○	●	●	●
-0.0333*** (4.254955e-007)	-0.0273*** (3.357250e-005)	○	○	○	●	●	○	●	●	●
-0.0376*** (1.073291e-008)	-0.0499*** (3.275158e-014)	●	●	●	●	●	○	●	○	○
0.0056 (3.949910e-001)	-0.0378*** (9.138643e-009)	●	●	●	●	●	○	○	●	○
-0.0516*** (4.218847e-015)	-0.0467*** (1.246891e-012)	●	●	●	●	●	○	○	○	●
-0.0025 (7.037890e-001)	-0.0396*** (1.756284e-009)	●	●	●	●	○	○	●	●	○
-0.0511*** (8.437695e-015)	-0.0463*** (1.997957e-012)	●	●	●	●	○	○	●	○	●
-0.0248** (1.681744e-004)	-0.0194* (3.152518e-003)	●	●	●	●	○	○	○	●	●
-0.0031 (6.429819e-001)	-0.0284*** (1.648349e-005)	●	●	○	●	●	○	●	●	○
-0.0492*** (7.582823e-014)	-0.0439*** (2.420208e-011)	●	●	○	●	●	○	●	○	●
-0.0271*** (3.954771e-005)	-0.0218** (9.357775e-004)	●	●	○	●	●	○	○	●	●
-0.0307*** (3.198966e-006)	-0.0251** (1.397952e-004)	●	●	○	●	○	○	●	●	●
0.0011 (8.636389e-001)	-0.0265*** (5.719229e-005)	●	○	●	●	●	○	●	●	○
-0.0485*** (1.647571e-013)	-0.0437*** (3.030720e-011)	●	○	●	●	●	○	●	○	●
-0.0219** (8.641822e-004)	-0.0168 (1.054101e-002)	●	○	●	●	●	○	○	●	●
-0.0289*** (1.137345e-005)	-0.0234** (3.772934e-004)	●	○	●	●	○	○	●	●	●
-0.0222** (7.449442e-004)	-0.0164 (1.279633e-002)	●	○	○	●	●	○	●	●	●
-0.0015 (8.165377e-001)	-0.0270*** (3.980764e-005)	○	●	●	●	●	○	●	●	○
-0.0608*** (0)	-0.0557*** (0)	○	●	●	●	●	○	●	○	●
-0.0309*** (2.630127e-006)	-0.0256** (1.008305e-004)	○	●	●	●	●	○	○	●	●
-0.0367*** (2.496203e-008)	-0.0310*** (2.399961e-006)	○	●	●	●	○	○	●	●	●
-0.0297*** (6.258532e-006)	-0.0238** (3.060795e-004)	○	●	○	●	●	○	●	●	●
-0.0321*** (1.044195e-006)	-0.0260*** (7.699792e-005)	○	○	●	●	●	○	●	●	●
-0.0031 (6.409233e-001)	-0.0284*** (1.599421e-005)	●	●	●	●	●	○	●	●	○

-0.0499*** (3.386180e-014)	-0.0450*** (7.682188e-012)	●	●	●	●	●	○	●	○	●
-0.0269*** (4.228621e-005)	-0.0216* (1.013987e-003)	●	●	●	●	●	○	○	●	●
-0.0307*** (3.080708e-006)	-0.0251** (1.332428e-004)	●	●	●	●	○	○	●	●	●
-0.0241** (2.433125e-004)	-0.0182* (5.698801e-003)	●	●	○	●	●	○	●	●	●
-0.0222** (7.631436e-004)	-0.0163 (1.326144e-002)	●	○	●	●	●	○	●	●	●
-0.0296*** (6.753615e-006)	-0.0236** (3.295283e-004)	○	●	●	●	●	○	●	●	●
-0.0242** (2.382187e-004)	-0.0183* (5.557461e-003)	●	●	●	●	●	○	●	●	●
-0.0266*** (5.412254e-005)	-0.0128 (5.116761e-002)	●	●	●	●	●	○	●	●	●
FD_n* and *θ_{neu}	***FD_n* and *P_{neu}***									
Watson θ_{neu}	Watson P_{neu}	RR	GC	RD	SC	D_n	D_x	FD_n	FD_x	d_{neu}
-0.1935*** (0)	-0.0781*** (0)	○	○	○	●	○	○	○	○	○
-0.2236*** (0)	-0.0923*** (0)	●	○	○	●	○	○	○	○	○
-0.2051*** (0)	-0.1009*** (0)	○	●	○	●	○	○	○	○	○
-0.2088*** (0)	-0.0960*** (0)	○	○	●	●	○	○	○	○	○
-0.1323*** (0)	-0.0109 (9.776066e-002)	○	○	○	●	●	○	○	○	○
-0.1799*** (0)	-0.0638*** (0)	○	○	○	●	○	●	○	○	○
-0.0414*** (3.159792e-010)	-0.0053 (4.201675e-001)	○	○	○	●	○	○	○	●	○
-0.0880*** (0)	-0.0855*** (0)	○	○	○	●	○	○	○	○	●
-0.1393*** (0)	-0.0567*** (0)	●	●	○	●	○	○	○	○	○
-0.2143*** (0)	-0.0945*** (0)	●	○	●	●	○	○	○	○	○
-0.1307*** (0)	-0.0054 (4.138431e-001)	●	○	○	●	●	○	○	○	○
-0.2118*** (0)	-0.0793*** (0)	●	○	○	●	○	●	○	○	○
-0.0774*** (0)	-0.0262*** (6.864408e-005)	●	○	○	●	○	○	○	●	○
-0.1313*** (0)	-0.1272*** (0)	●	○	○	●	○	○	○	○	●
-0.2112*** (0)	-0.1071*** (0)	○	●	●	●	○	○	○	○	○
-0.1483*** (0)	-0.0287*** (1.281598e-005)	○	●	○	●	●	○	○	○	○
-0.1927*** (0)	-0.0875*** (0)	○	●	○	●	○	●	○	○	○
-0.0661*** (0)	-0.0280*** (2.116490e-005)	○	●	○	●	○	○	○	●	○
-0.1104*** (0)	-0.1079*** (0)	○	●	○	●	○	○	○	○	●
-0.1432*** (0)	-0.0216* (1.019326e-003)	○	○	●	●	●	○	○	○	○
-0.1958*** (0)	-0.0822*** (0)	○	○	●	●	○	●	○	○	○
-0.0528*** (9.992007e-016)	-0.0154 (1.952601e-002)	○	○	●	●	○	○	○	●	○
-0.1040*** (0)	-0.1039*** (0)	○	○	●	●	○	○	○	○	●

-0.1296*** (0)	-0.0077 (2.432916e-001)	○	○	○	●	●	●	○	○	○
-0.0390*** (3.082556e-009)	0.0340*** (2.291219e-007)	○	○	○	●	●	○	○	●	○
-0.0226** (5.924553e-004)	-0.0202* (2.188146e-003)	○	○	○	●	●	○	○	○	●
-0.0404*** (7.905960e-010)	-0.0150 (2.285898e-002)	○	○	○	●	○	●	○	●	○
-0.0727*** (0)	-0.0713*** (0)	○	○	○	●	○	●	○	○	●
-0.0105 (1.098125e-001)	-0.0095 (1.489428e-001)	○	○	○	●	○	○	○	●	●
-0.1397*** (0)	-0.0602*** (0)	●	●	●	●	○	○	○	○	○
-0.0954*** (0)	0.0070 (2.849846e-001)	●	●	○	●	●	○	○	○	○
-0.1301*** (0)	-0.0457*** (3.654299e-012)	●	●	○	●	○	●	○	○	○
-0.0420*** (1.731276e-010)	-0.0123 (6.186619e-002)	●	●	○	●	○	○	○	●	○
-0.0853*** (0)	-0.0835*** (0)	●	●	○	●	○	○	○	○	●
-0.1287*** (0)	-0.0085 (1.956931e-001)	●	○	●	●	●	○	○	○	○
-0.2040*** (0)	-0.0825*** (0)	●	○	●	●	○	●	○	○	○
-0.0773*** (0)	-0.0287*** (1.328959e-005)	●	○	●	●	○	○	○	●	○
-0.1288*** (0)	-0.1274*** (0)	●	○	●	●	○	○	○	○	●
-0.1288*** (0)	-0.0027 (6.773881e-001)	●	○	○	●	●	●	○	○	○
-0.0493*** (7.038814e-014)	0.0298*** (6.094484e-006)	●	○	○	●	●	○	○	●	○
-0.0445*** (1.352940e-011)	-0.0412*** (3.678571e-010)	●	○	○	●	●	○	○	○	●
-0.0745*** (0)	-0.0348*** (1.194868e-007)	●	○	○	●	○	●	○	●	○
-0.1174*** (0)	-0.1145*** (0)	●	○	○	●	○	●	○	○	●
-0.0450*** (7.788992e-012)	-0.0428*** (7.778145e-011)	●	○	○	●	○	○	○	●	●
-0.1526*** (0)	-0.0328*** (6.294174e-007)	○	●	●	●	●	○	○	○	○
-0.1987*** (0)	-0.0936*** (0)	○	●	●	●	○	●	○	○	○
-0.0683*** (0)	-0.0298*** (5.781531e-006)	○	●	●	●	○	○	○	●	○
-0.1160*** (0)	-0.1146*** (0)	○	●	●	●	○	○	○	○	●
-0.1452*** (0)	-0.0251** (1.353067e-004)	○	●	○	●	●	●	○	○	○
-0.0542*** (1.110223e-016)	0.0197* (2.800023e-003)	○	●	○	●	●	○	○	●	○
-0.0404*** (8.338269e-010)	-0.0378*** (8.818483e-009)	○	●	○	●	●	○	○	○	●
-0.0640*** (0)	-0.0354*** (7.745422e-008)	○	●	○	●	○	●	○	●	○
-0.0959*** (0)	-0.0945*** (0)	○	●	○	●	○	●	○	○	●
-0.0339*** (2.514702e-007)	-0.0327*** (6.593281e-007)	○	●	○	●	○	○	○	●	●
-0.1405*** (0)	-0.0185* (4.883318e-003)	○	○	●	●	●	●	○	○	○
-0.0451*** (7.441159e-012)	0.0288*** (1.201840e-005)	○	○	●	●	●	○	○	●	○

-0.0330*** (5.215428e-007)	-0.0318*** (1.315214e-006)	○	○	●	●	●	○	○	○	●
-0.0513*** (5.995204e-015)	-0.0245** (1.994942e-004)	○	○	●	●	○	●	○	●	○
-0.0892*** (0)	-0.0901*** (0)	○	○	●	●	○	●	○	○	●
-0.0203* (1.997783e-003)	-0.0204* (1.950335e-003)	○	○	●	●	○	○	○	●	●
-0.0387*** (4.065688e-009)	0.0230** (4.739386e-004)	○	○	○	●	●	●	○	●	○
-0.0188* (4.352498e-003)	-0.0167 (1.140233e-002)	○	○	○	●	●	●	○	○	●
0.0249** (1.513886e-004)	0.0265*** (5.545702e-005)	○	○	○	●	●	○	○	●	●
-0.0199* (2.450836e-003)	-0.0176* (7.592026e-003)	○	○	○	●	○	●	○	●	●
-0.0962*** (0)	0.0044 (5.085925e-001)	●	●	●	●	●	○	○	○	○
-0.1308*** (0)	-0.0494*** (5.639933e-014)	●	●	●	●	○	●	○	○	○
-0.0428*** (7.963252e-011)	-0.0136 (3.859460e-002)	●	●	●	●	○	○	○	●	○
-0.0868*** (0)	-0.0864*** (0)	●	●	●	●	○	○	○	○	●
-0.0931*** (0)	0.0099 (1.323080e-001)	●	●	○	●	●	●	○	○	○
-0.0284*** (1.581379e-005)	0.0370*** (1.911242e-008)	●	●	○	●	●	○	○	●	○
-0.0275*** (2.943987e-005)	-0.0253** (1.192789e-004)	●	●	○	●	●	○	○	○	●
-0.0415*** (2.822301e-010)	-0.0207* (1.619575e-003)	●	●	○	●	○	●	○	●	○
-0.0736*** (0)	-0.0729*** (0)	●	●	○	●	○	●	○	○	●
-0.0237** (3.249916e-004)	-0.0228** (5.417477e-004)	●	●	○	●	○	○	○	●	●
-0.1270*** (0)	-0.0061 (3.511800e-001)	●	○	●	●	●	●	○	○	○
-0.0494*** (6.172840e-014)	0.0282*** (1.872341e-005)	●	○	●	●	●	○	○	●	○
-0.0449*** (8.630985e-012)	-0.0431*** (5.564360e-011)	●	○	●	●	●	○	○	○	●
-0.0745*** (0)	-0.0371*** (1.674496e-008)	●	○	●	●	○	●	○	●	○
-0.1160*** (0)	-0.1156*** (0)	●	○	●	●	○	●	○	○	●
-0.0459*** (3.106293e-012)	-0.0447*** (1.058842e-011)	●	○	●	●	○	○	○	●	●
-0.0465*** (1.615486e-012)	0.0200* (2.349320e-003)	●	○	○	●	●	●	○	●	○
-0.0412*** (3.803508e-010)	-0.0383*** (5.949674e-009)	●	○	○	●	●	●	○	○	●
0.0031 (6.383806e-001)	0.0054 (4.084116e-001)	●	○	○	●	●	○	○	●	●
-0.0505*** (1.554312e-014)	-0.0471*** (8.107959e-013)	●	○	○	●	○	●	○	●	●
-0.1496*** (0)	-0.0292*** (9.144191e-006)	○	●	●	●	●	●	○	○	○
-0.0551*** (0)	0.0190* (3.814409e-003)	○	●	●	●	●	○	○	●	○
-0.0445*** (1.296530e-011)	-0.0427*** (8.638967e-011)	○	●	●	●	●	○	○	○	●
-0.0662*** (0)	-0.0372*** (1.528546e-008)	○	●	●	●	○	●	○	●	○
-0.1015*** (0)	-0.1011*** (0)	○	●	●	●	○	●	○	○	●

-0.0359*** (4.735971e-008)	-0.0350*** (1.018141e-007)	○	●	●	●	○	○	○	●	●
-0.0522*** (2.109424e-015)	0.0108 (1.003835e-001)	○	●	○	●	●	●	○	●	○
-0.0361*** (3.963196e-008)	-0.0340*** (2.423577e-007)	○	●	○	●	●	●	○	○	●
0.0100 (1.278130e-001)	0.0117 (7.471089e-002)	○	●	○	●	●	○	○	●	●
-0.0410*** (4.765931e-010)	-0.0386*** (4.493879e-009)	○	●	○	●	○	●	○	●	●
-0.0441*** (1.939859e-011)	0.0183* (5.364198e-003)	○	○	●	●	●	●	○	●	○
-0.0293*** (8.268672e-006)	-0.0284*** (1.543117e-005)	○	○	●	●	●	●	○	○	●
0.0192* (3.477254e-003)	0.0202* (2.109571e-003)	○	○	●	●	●	○	○	●	●
-0.0291*** (9.976708e-006)	-0.0277*** (2.497326e-005)	○	○	●	●	○	●	○	●	●
0.0141 (3.259487e-002)	0.0173* (8.393551e-003)	○	○	○	●	●	●	○	●	●
-0.0941*** (0)	0.0071 (2.815810e-001)	●	●	●	●	●	●	○	○	○
-0.0289*** (1.147693e-005)	0.0362*** (3.727909e-008)	●	●	●	●	●	○	○	●	○
-0.0289*** (1.157567e-005)	-0.0276*** (2.734775e-005)	●	●	●	●	●	○	○	○	●
-0.0423*** (1.324859e-010)	-0.0221** (8.074637e-004)	●	●	●	●	○	●	○	●	○
-0.0754*** (0)	-0.0759*** (0)	●	●	●	●	○	●	○	○	●
-0.0245** (1.955387e-004)	-0.0241** (2.578278e-004)	●	●	●	●	○	○	○	●	●
-0.0280*** (2.033966e-005)	0.0270*** (4.155628e-005)	●	●	○	●	●	●	○	●	○
-0.0241** (2.532974e-004)	-0.0223** (7.165957e-004)	●	●	○	●	●	●	○	○	●
0.0138 (3.548500e-002)	0.0154 (1.898969e-002)	●	●	○	●	●	○	○	●	●
-0.0297*** (6.594587e-006)	-0.0276*** (2.758187e-005)	●	●	○	●	○	●	○	●	●
-0.0466*** (1.423417e-012)	0.0186* (4.757621e-003)	●	○	●	●	●	●	○	●	○
-0.0419*** (1.914262e-010)	-0.0404*** (8.298146e-010)	●	○	●	●	●	●	○	○	●
0.0025 (7.064700e-001)	0.0042 (5.187732e-001)	●	○	●	●	●	○	○	●	●
-0.0513*** (5.995204e-015)	-0.0489*** (1.005862e-013)	●	○	●	●	○	●	○	●	●
-0.0035 (5.993108e-001)	0.0005 (9.429744e-001)	●	○	○	●	●	●	○	●	●
-0.0531*** (6.661338e-016)	0.0102 (1.225383e-001)	○	●	●	●	●	●	○	●	○
-0.0403*** (8.724362e-010)	-0.0388*** (3.570962e-009)	○	●	●	●	●	●	○	○	●
0.0089 (1.787638e-001)	0.0104 (1.143547e-001)	○	●	●	●	●	○	○	●	●
-0.0429*** (7.003831e-011)	-0.0408*** (5.517281e-010)	○	●	●	●	○	●	○	●	●
0.0013 (8.380366e-001)	0.0046 (4.799739e-001)	○	●	○	●	●	●	○	●	●
0.0090 (1.706599e-001)	0.0118 (7.395215e-002)	○	○	●	●	●	●	○	●	●
-0.0285*** (1.496840e-005)	0.0262*** (6.730955e-005)	●	●	●	●	●	●	○	●	○
-0.0256** (1.005141e-004)	-0.0247** (1.799813e-004)	●	●	●	●	●	●	○	○	●

0.0134 (4.248445e-002)	0.0147 (2.529325e-002)	●	●	●	●	●	○	○	●	●
-0.0305*** (3.571359e-006)	-0.0289*** (1.157296e-005)	●	●	●	●	○	●	○	●	●
0.0066 (3.191892e-001)	0.0098 (1.384279e-001)	●	●	○	●	●	●	○	●	●
-0.0040 (5.413504e-001)	-0.0006 (9.227972e-001)	●	○	●	●	●	●	○	●	●
0.0003 (9.695487e-001)	0.0034 (6.053117e-001)	○	●	●	●	●	●	○	●	●
0.0061 (3.556625e-001)	0.0091 (1.691375e-001)	●	●	●	●	●	●	○	●	●
0.0223** (7.155404e-004)	0.0070 (2.860453e-001)	●	●	●	●	●	●	○	●	●
***FD_x* and θ_{neu}**	***FD_x* and P_{neu}**									
Watson θ_{neu}	Watson P_{neu}	RR	GC	RD	SC	D_n	D_x	FD_n	FD_x	d_{neu}
-0.2124*** (0)	-0.0941*** (0)	○	○	○	●	○	○	○	○	○
-0.2233*** (0)	-0.0960*** (0)	●	○	○	●	○	○	○	○	○
-0.2172*** (0)	-0.1103*** (0)	○	●	○	●	○	○	○	○	○
-0.2264*** (0)	-0.1109*** (0)	○	○	●	●	○	○	○	○	○
-0.1553*** (0)	-0.0543*** (1.110223e-016)	○	○	○	●	●	○	○	○	○
-0.1951*** (0)	-0.0685*** (0)	○	○	○	●	○	●	○	○	○
-0.0983*** (0)	-0.0529*** (8.881784e-016)	○	○	○	●	○	○	●	○	○
-0.1053*** (0)	-0.1029*** (0)	○	○	○	●	○	○	○	○	●
-0.1515*** (0)	-0.0659*** (0)	●	●	○	●	○	○	○	○	○
-0.2141*** (0)	-0.0982*** (0)	●	○	●	●	○	○	○	○	○
-0.1415*** (0)	-0.0412*** (3.648929e-010)	●	○	○	●	●	○	○	○	○
-0.2100*** (0)	-0.0730*** (0)	●	○	○	●	○	●	○	○	○
-0.0766*** (0)	-0.0373*** (1.409639e-008)	●	○	○	●	○	○	●	○	○
-0.1336*** (0)	-0.1301*** (0)	●	○	○	●	○	○	○	○	●
-0.2243*** (0)	-0.1174*** (0)	○	●	●	●	○	○	○	○	○
-0.1651*** (0)	-0.0668*** (0)	○	●	○	●	●	○	○	○	○
-0.2019*** (0)	-0.0857*** (0)	○	●	○	●	○	●	○	○	○
-0.0985*** (0)	-0.0529*** (8.881784e-016)	○	●	○	●	○	○	●	○	○
-0.1205*** (0)	-0.1182*** (0)	○	●	○	●	○	○	○	○	●
-0.1663*** (0)	-0.0654*** (0)	○	○	●	●	●	○	○	○	○
-0.2097*** (0)	-0.0854*** (0)	○	○	●	●	○	●	○	○	○
-0.1040*** (0)	-0.0578*** (0)	○	○	●	●	○	○	●	○	○
-0.1203*** (0)	-0.1200*** (0)	○	○	●	●	○	○	○	○	●
-0.1452*** (0)	-0.0340*** (2.414689e-007)	○	○	○	●	●	●	○	○	○

-0.0907*** (0)	-0.0631*** (0)	○	○	○	●	●	○	●	○	○
-0.0656*** (0)	-0.0637*** (0)	○	○	○	●	●	○	○	○	●
-0.0868*** (0)	-0.0292*** (8.886206e-006)	○	○	○	●	○	●	●	○	○
-0.0774*** (0)	-0.0776*** (0)	○	○	○	●	○	●	○	○	●
-0.0590*** (0)	-0.0582*** (0)	○	○	○	●	○	○	●	○	●
-0.1522*** (0)	-0.0700*** (0)	●	●	●	●	○	○	○	○	○
-0.1148*** (0)	-0.0332*** (4.642993e-007)	●	●	○	●	●	○	○	○	○
-0.1387*** (0)	-0.0430*** (6.228418e-011)	●	●	○	●	○	●	○	○	○
-0.0732*** (0)	-0.0358*** (5.105222e-008)	●	●	○	●	○	○	●	○	○
-0.0958*** (0)	-0.0942*** (0)	●	●	○	●	○	○	○	○	●
-0.1396*** (0)	-0.0451*** (7.201351e-012)	●	○	●	●	●	○	○	○	○
-0.2021*** (0)	-0.0759*** (0)	●	○	●	●	○	●	○	○	○
-0.0766*** (0)	-0.0393*** (2.328287e-009)	●	○	●	●	○	○	●	○	○
-0.1311*** (0)	-0.1302*** (0)	●	○	●	●	○	○	○	○	●
-0.1363*** (0)	-0.0240** (2.660176e-004)	●	○	○	●	●	●	○	○	○
-0.0735*** (0)	-0.0506*** (1.509903e-014)	●	○	○	●	●	○	●	○	○
-0.0735*** (0)	-0.0713*** (0)	●	○	○	●	●	○	○	○	●
-0.0689*** (0)	-0.0160 (1.533679e-002)	●	○	○	●	○	●	●	○	○
-0.1107*** (0)	-0.1098*** (0)	●	○	○	●	○	●	○	○	●
-0.0513*** (6.439294e-015)	-0.0507*** (1.310063e-014)	●	○	○	●	○	○	●	○	●
-0.1712*** (0)	-0.0726*** (0)	○	●	●	●	●	○	○	○	○
-0.2086*** (0)	-0.0923*** (0)	○	●	●	●	○	●	○	○	○
-0.1031*** (0)	-0.0569*** (0)	○	●	●	●	○	○	●	○	○
-0.1271*** (0)	-0.1259*** (0)	○	●	●	●	○	○	○	○	●
-0.1566*** (0)	-0.0478*** (3.516076e-013)	○	●	○	●	●	●	○	○	○
-0.0912*** (0)	-0.0634*** (0)	○	●	○	●	●	○	●	○	○
-0.0776*** (0)	-0.0756*** (0)	○	●	○	●	●	○	○	○	●
-0.0885*** (0)	-0.0305*** (3.549334e-006)	○	●	○	●	○	●	●	○	○
-0.0936*** (0)	-0.0939*** (0)	○	●	○	●	○	●	○	○	●
-0.0593*** (0)	-0.0586*** (0)	○	●	○	●	○	○	●	○	●
-0.1564*** (0)	-0.0452*** (6.330714e-012)	○	○	●	●	●	●	○	○	○
-0.0965*** (0)	-0.0681*** (0)	○	○	●	●	●	○	●	○	○
-0.0763*** (0)	-0.0757*** (0)	○	○	●	●	●	○	○	○	●

-0.0922*** (0)	-0.0338*** (2.718598e-007)	○	○	●	●	○	●	●	○	○
-0.0927*** (0)	-0.0950*** (0)	○	○	●	●	○	●	○	○	●
-0.0640*** (0)	-0.0638*** (0)	○	○	●	●	○	○	●	○	●
-0.0766*** (0)	-0.0403*** (9.038958e-010)	○	○	○	●	●	●	●	○	○
-0.0432*** (5.089296e-011)	-0.0437*** (3.189959e-011)	○	○	○	●	●	●	○	○	●
-0.0665*** (0)	-0.0660*** (0)	○	○	○	●	●	○	●	○	●
-0.0335*** (3.611074e-007)	-0.0354*** (7.300875e-008)	○	○	○	●	○	●	●	○	●
-0.1162*** (0)	-0.0369*** (2.118730e-008)	●	●	●	●	●	○	○	○	○
-0.1397*** (0)	-0.0470*** (8.596457e-013)	●	●	●	●	○	●	○	○	○
-0.0744*** (0)	-0.0382*** (6.234279e-009)	●	●	●	●	○	○	●	○	○
-0.0977*** (0)	-0.0977*** (0)	●	●	●	●	○	○	○	○	●
-0.1069*** (0)	-0.0145 (2.714445e-002)	●	●	○	●	●	●	○	○	○
-0.0702*** (0)	-0.0492*** (7.838175e-014)	●	●	○	●	●	○	●	○	○
-0.0618*** (0)	-0.0603*** (0)	●	●	○	●	●	○	○	○	●
-0.0639*** (0)	-0.0138 (3.629322e-002)	●	●	○	●	○	●	●	○	○
-0.0737*** (0)	-0.0746*** (0)	●	●	○	●	○	●	○	○	●
-0.0499*** (3.419487e-014)	-0.0494*** (6.172840e-014)	●	●	○	●	○	○	●	○	●
-0.1346*** (0)	-0.0279*** (2.294810e-005)	●	○	●	●	●	●	○	○	○
-0.0735*** (0)	-0.0525*** (1.554312e-015)	●	○	●	●	●	○	●	○	○
-0.0744*** (0)	-0.0737*** (0)	●	○	●	●	●	○	○	○	●
-0.0690*** (0)	-0.0178* (6.717233e-003)	●	○	●	●	○	●	●	○	○
-0.1091*** (0)	-0.1106*** (0)	●	○	●	●	○	●	○	○	●
-0.0520*** (2.664535e-015)	-0.0523*** (1.887379e-015)	●	○	●	●	○	○	●	○	●
-0.0647*** (0)	-0.0311*** (2.241084e-006)	●	○	○	●	●	●	●	○	○
-0.0574*** (0)	-0.0574*** (0)	●	○	○	●	●	●	○	○	●
-0.0587*** (0)	-0.0584*** (0)	●	○	○	●	●	○	●	○	●
-0.0319*** (1.231150e-006)	-0.0339*** (2.507954e-007)	●	○	○	●	○	●	●	○	●
-0.1623*** (0)	-0.0532*** (5.551115e-016)	○	●	●	●	●	●	○	○	○
-0.0958*** (0)	-0.0675*** (0)	○	●	●	●	●	○	●	○	○
-0.0832*** (0)	-0.0822*** (0)	○	●	●	●	●	○	○	○	●
-0.0925*** (0)	-0.0340*** (2.356091e-007)	○	●	●	●	○	●	●	○	○
-0.0999*** (0)	-0.1013*** (0)	○	●	●	●	○	●	○	○	●
-0.0633*** (0)	-0.0632*** (0)	○	●	●	●	○	○	●	○	●

-0.0788*** (0)	-0.0421*** (1.500949e-010)	○	●	○	●	●	●	●	○	○
-0.0566*** (0)	-0.0571*** (0)	○	●	○	●	●	●	○	○	●
-0.0670*** (0)	-0.0666*** (0)	○	●	○	●	●	○	●	○	●
-0.0353*** (8.072111e-008)	-0.0372*** (1.519601e-008)	○	●	○	●	○	●	●	○	●
-0.0821*** (0)	-0.0451*** (6.909473e-012)	○	○	●	●	●	●	●	○	○
-0.0541*** (2.220446e-016)	-0.0558*** (0)	○	○	●	●	●	●	○	○	●
-0.0715*** (0)	-0.0716*** (0)	○	○	●	●	●	○	●	○	●
-0.0385*** (4.955137e-009)	-0.0410*** (4.666496e-010)	○	○	●	●	○	●	●	○	●
-0.0414*** (3.153103e-010)	-0.0439*** (2.415557e-011)	○	○	○	●	●	●	●	○	●
-0.1083*** (0)	-0.0181* (5.967599e-003)	●	●	●	●	●	●	○	○	○
-0.0715*** (0)	-0.0515*** (5.107026e-015)	●	●	●	●	●	○	●	○	○
-0.0639*** (0)	-0.0636*** (0)	●	●	●	●	●	○	○	○	●
-0.0651*** (0)	-0.0160 (1.538201e-002)	●	●	●	●	○	●	●	○	○
-0.0757*** (0)	-0.0780*** (0)	●	●	●	●	○	●	○	○	●
-0.0514*** (5.884182e-015)	-0.0517*** (4.107825e-015)	●	●	●	●	○	○	●	○	●
-0.0597*** (0)	-0.0290*** (1.051654e-005)	●	●	○	●	●	●	●	○	○
-0.0443*** (1.679223e-011)	-0.0452*** (6.419865e-012)	●	●	○	●	●	●	○	○	●
-0.0571*** (0)	-0.0569*** (0)	●	●	○	●	●	○	●	○	●
-0.0298*** (5.798786e-006)	-0.0320*** (1.169517e-006)	●	●	○	●	○	●	●	○	●
-0.0647*** (0)	-0.0329*** (5.653553e-007)	●	○	●	●	●	●	●	○	○
-0.0584*** (0)	-0.0599*** (0)	●	○	●	●	●	●	○	○	●
-0.0594*** (0)	-0.0600*** (0)	●	○	●	●	●	○	●	○	●
-0.0326*** (7.135294e-007)	-0.0354*** (7.381977e-008)	●	○	●	●	○	●	●	○	●
-0.0402*** (1.047718e-009)	-0.0428*** (8.005774e-011)	●	○	○	●	●	●	●	○	●
-0.0829*** (0)	-0.0457*** (3.841261e-012)	○	●	●	●	●	●	●	○	○
-0.0620*** (0)	-0.0633*** (0)	○	●	●	●	●	●	○	○	●
-0.0709*** (0)	-0.0711*** (0)	○	●	●	●	●	○	●	○	●
-0.0389*** (3.284590e-009)	-0.0414*** (3.087270e-010)	○	●	●	●	○	●	●	○	●
-0.0436*** (3.425116e-011)	-0.0461*** (2.332579e-012)	○	●	○	●	●	●	●	○	●
-0.0463*** (1.864064e-012)	-0.0495*** (5.573320e-014)	○	○	●	●	●	●	●	○	●
-0.0609*** (0)	-0.0311*** (2.349920e-006)	●	●	●	●	●	●	●	○	○
-0.0463*** (1.953104e-012)	-0.0483*** (2.108314e-013)	●	●	●	●	●	●	○	○	●
-0.0586*** (0)	-0.0592*** (0)	●	●	●	●	●	○	●	○	●

-0.0312*** (2.125144e-006)	-0.0340*** (2.302750e-007)	●	●	●	●	○	●	●	○	●
-0.0378*** (9.620777e-009)	-0.0405*** (7.276389e-010)	●	●	○	●	●	●	●	○	●
-0.0408*** (5.393536e-010)	-0.0442*** (1.795986e-011)	●	○	●	●	●	●	●	○	●
-0.0471*** (8.185674e-013)	-0.0502*** (2.431388e-014)	○	●	●	●	●	●	●	○	●
-0.0391*** (2.892226e-009)	-0.0425*** (1.031711e-010)	●	●	●	●	●	●	●	○	●
-0.0553*** (4.113460e-017)	-0.0433*** (4.453422e-011)	●	●	●	●	●	●	●	○	●

*** $P<0.0001$; ** $0.0001 \le P<0.001$; * $0.001 \le P<0.01$, Spearman test
